# Six Undescribed Capnosane-Type Macrocyclic Diterpenoids from South China Sea Soft Coral *Sarcophyton crassocaule*: Structural Determination and Biological Evaluation

**DOI:** 10.3390/md21120645

**Published:** 2023-12-18

**Authors:** Hanyang Peng, Yanbo Zeng, Hao Wang, Wenjun Chang, Huiqin Chen, Fengjuan Zhou, Haofu Dai, Xiachang Wang

**Affiliations:** 1 Hainan Provincial Key Laboratory for Functional Components Research and Utilization of Marine Bio-Resources, Institute of Tropical Bioscience and Biotechnology, Chinese Academy of Tropical Agricultural Sciences & Key Laboratory for Biology and Genetic Resources of Tropical Crops of Hainan Province, Hainan Institute for Tropical Agricultural Resources, Haikou 571101, China; 17379935106@163.com (H.P.); wanghao@itbb.org.cn (H.W.); changwenjun@itbb.org.cn (W.C.); chenhuiqin@itbb.org.cn (H.C.); 2 Jiangsu Key Laboratory for Functional Substances of Chinese Medicine, Nanjing University of Chinese Medicine, Nanjing 210023, China; 20210662@njucm.edu.cn; 3 Zhanjiang Experimental Station of Chinese Academy of Tropical Agricultural Sciences, Zhanjiang 524013, China

**Keywords:** soft coral, *Sarcophyton crassocaule*, cembranoids, capnosane-type macrocyclic diterpenes, absolute configuration, anti-inflammation

## Abstract

Six undescribed capnosane-type macrocyclic diterpenes sarcocrassolins A–F (**1**–**6**) and one related known analog pavidolide D (**7**) were isolated from *Sarcophyton crassocaule*, a soft coral collected off the Nansha Islands, in the South China Sea. Their complete structures, relative configurations and absolute configurations were established through comprehensive spectroscopic analysis, quantum mechanical nuclear magnetic resonance (QM-NMR) and single-crystal X-ray diffraction. Sarcocrassolins D (**4**) and E (**5**) showed inhibitory activity against lipopolysaccharide (LPS)-stimulated inflammatory responses in RAW264.7 cells with IC_50_ values of 76.8 ± 8.0 μM and 93.0 ± 3.8 μM, respectively.

## 1. Introduction

Cembranoids are a large family of diterpenoids having a highly flexible 14-membered macrocyclic skeleton, which is generated from geranylgeranyl diphosphate (GGPP) by head-to-tail cyclization [1]. The first cembranoid, named cembrene, was isolated from the oleoresin of the white bark pine in 1962 [2]. Afterward, plenty of cembranoids have been isolated from plants, insects or marine organisms. The capnosane-type macrocyclic diterpenes, which have a 5/11-fused bicyclic carbon skeleton, are thought to be biogenetically produced from cembranoids via transannular cyclization between the C-3 and C-7 positions or ring rearrangement [3,4,5]. Capnosane-type macrocyclic diterpenes are rarely encountered. Hitherto, only 48 capnosane-type macrocyclic diterpenes have been reported from 14 species of soft corals (including *Sarcophyton boettgeri* [3], *Sarcophyton trocheliophorum* [6,7,8,9], *Klyxum flaccidum* [10], *Sinularia rigida* [11], *Sinularia polydactyla* [12], *Sarcophyton mililatensis* [13], *Sinularia pavida* [14], *Sarcophyton elegans* [15], *Lobophytum* sp. [16], *Sarcophyton solidun* [17], *Cespitularia* sp. [18], *Alcyonium coralloides* [19], *Lobophytum pauciflorum* [20], and *Sinularia humilis* [21]). Moreover, these capnosane-type macrocyclic diterpenes have been proved to possess a wide variety of biological activities, including anticancer [10,15], anti-inflammatory [3,20] and inhibition of tyrosine protein phosphatase 1B [8,12].

**Figure 1:**
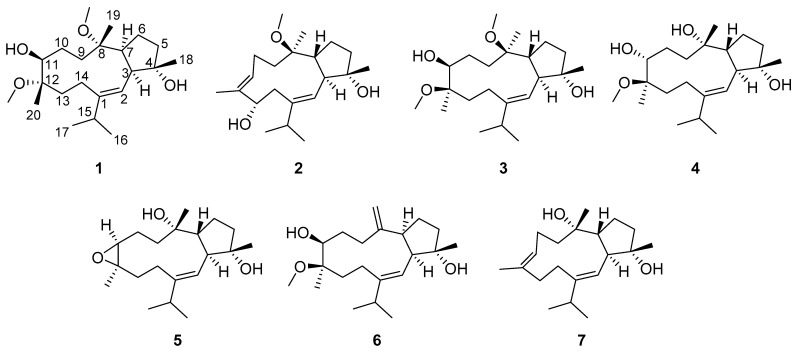
Structures of compounds **1**–**7** from the soft coral *Sarcophyton crassocaule*.

Soft corals of genus *Sarcophyton* are widely distributed in shallow waters of the Mediterranean Sea, Red Sea, Arctic and Indo-Pacific region [22]. Besides, the genus *Sarcophyton*, which includes many species, is a rich source of bioactive natural metabolites. Most of these species of the genus *Sarcophyton* have undergone thorough chemical investigations [23], including the species *Sarcophyton crassocaule*. Despite the fact that many diterpenoids were isolated from *S. crassocaule*, capnosane-type macrocyclic diterpenes have not been encountered. In our continuous search for pharmaceutical leads from structurally diverse secondary metabolites of soft corals [24], six undescribed capnosane-type macrocyclic diterpenes named sarcocrassolins A–F (**1**–**6**) and one related known analogue, pavidolide D (**7**), (Figure 1) were isolated from *S. crassocaule*, a soft coral collected off the Nansha Islands. This is the first report of capnosane-type macrocyclic diterpenes in the soft coral *S. crassocaule*. Herein, we discuss the isolation, structural elucidation, biological activity and plausible biosynthetic pathway of these capnosane-type macrocyclic diterpenes.

## 2. Results and Discussion

### 2.1. Structure Elucidation of New Compounds **1**–**6**

**Figure 2:**
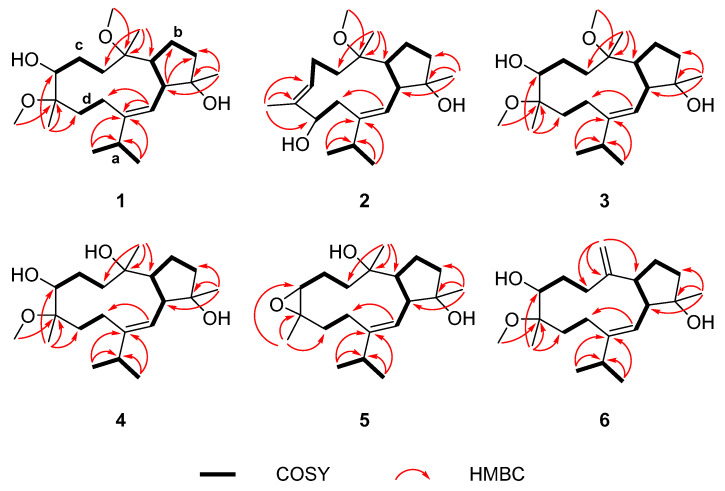
^1^H–^1^H COSY and key HMBC correlations of compounds **1**–**6**.

Sarcocrassolin A (**1**) was isolated as a white powder. The HRESIMS data at *m*/*z* 391.2820 (calculated as 391.2819 for C_22_H_40_O_4_Na, [M + Na]^+^) revealed the molecular formula of **1** to be C_22_H_40_O_4_ with three indexes of hydrogen deficit, which was validated by the ^13^C NMR and DEPT data. The presence of hydroxy and olefinic groups was suggested by the IR absorption bands at 3418 and 1638 cm^−1^, respectively. Five methyl groups (*δ*_H_ 0.99, 1.07, 1.08, 1.10, 1.26), two methoxy groups (*δ*_H_ 3.19, 3.22), one oxymethine (*δ*_H_ 3.59) and one olefinic proton (*δ*_H_ 5.02) were detected in the ^1^H NMR spectrum of **1** (Table 1). The ^13^C NMR spectrum revealed twenty-two carbon signals, including four quaternary carbons (*δ*_C_ 146.7, 83.2, 79.4, 79.3), five methines (*δ*_C_ 121.0, 78.7, 52.0, 50.0, 32.8), six methylenes (*δ*_C_ 37.5, 23.1, 35.7, 25.1, 33.2, 21.3), five methyls (*δ*_C_ 21.6, 23.1, 26.5, 19.2, 19.1) and two methoxy groups (*δ*_C_ 48.1, 49.5) (Table 2). A double bond in **1** accounted for one degree of unsaturation and indicated that the structure had two rings as a result of the remaining two indexes of hydrogen deficit. Because **1** and pavidolide D (**7**) were co-isolated secondary metabolites, their similar NMR data suggested that **1** was most likely a diterpene of the capnosane-type. Following that, the planar structure of **1** was ascertained by the ^1^H-^1^H COSY and HMBC spectra. First of all, four fragments **a**–**d** (Figure 2) were established by comprehensive analysis of the ^1^H-^1^H COSY correlations of H_3_-16 (*δ*_H_ 1.08)/H-15 (*δ*_H_ 2.23)/H_3_-17 (*δ*_H_ 0.99) (**a**), H-2 (*δ*_H_ 5.02)/H-3 (*δ*_H_ 2.49)/H-7 (*δ*_H_ 2.88)/H_2_-6 (*δ*_H_ 1.86, 1.75)/H_2_-5 (*δ*_H_ 1.77, 1.66) (**b**), H_2_-9 (*δ*_H_ 1.97, 1.62)/H_2_-10 (*δ*_H_ 1.56, 1.32)/H-11 (*δ*_H_ 3.59) (**c**) and H_2_-13 (*δ*_H_ 2.15, 1.19)/H_2_-14 (*δ*_H_ 2.09, 1.97) (**d**). The HMBC cross peaks from H-2 (*δ*_H_ 5.02) to C-1 (*δ*_C_ 146.7)/C-14 (*δ*_C_ 21.3), and from H_3_-16 (*δ*_H_ 1.08), H_3_-17 (*δ*_H_ 0.99) to C-1 (*δ*_C_ 146.7)/C-15 (*δ*_C_ 32.8) determined that fragments **a**, **b** and **d** were linked via C-1. The presence of a cyclopentane was demonstrated by the HMBC cross peaks from H_3_-18 (*δ*_H_ 1.10) to C-3 (*δ*_C_ 52.0)/C-4 (*δ*_C_ 83.2)/C-5 (*δ*_C_ 37.5) as well as from H-3 (*δ*_H_ 2.49) to C-5 (*δ*_C_ 37.5)/C-6 (*δ*_C_ 23.1). The fragments **b** and **c** were found to be linked via C-8, whereas fragments **c** and **d** via C-12, according to the HMBC cross peaks from H_3_-19 (*δ*_H_ 1.07) to C-7 (*δ*_C_ 50.0)/C-8 (*δ*_C_ 79.3)/C-9 (*δ*_C_ 35.7) and from H_3_-20 (*δ*_H_ 1.26) to C-11 (*δ*_C_ 78.7)/C-12 (*δ*_C_ 79.4)/C-13 (*δ*_C_ 33.2). Finally, the HMBC cross peaks from 8-OMe (*δ*_H_ 3.19) to C-8 (*δ*_C_ 79.3) and from 12-OMe (*δ*_H_ 3.22) to C-12 (*δ*_C_ 79.4) were utilized to infer the position of two methoxy groups, one at C-8 and the other at C-12. Thus, as can be seen in Figure 1, the complete planar structure of **1** was determined.

**Figure 3:**
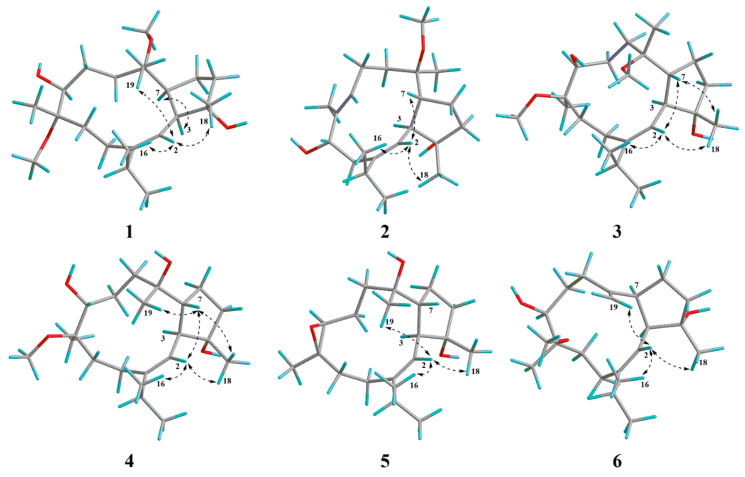
Key ROESY correlations of compounds **1**–**6**.

**Figure 4:**
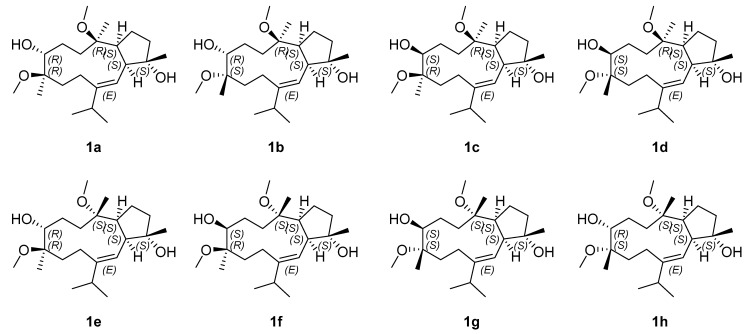
Structures of isomers of compound **1**.

The significant ROESY cross peaks between H-2 (*δ*_H_ 5.02) and H_3_-16 (*δ*_H_ 1.08) indicated the double-bond *Δ*^1^ in **1** in *E*-geometry. The ROESY cross peaks between H-3 (*δ*_H_ 2.49)/H-7 (*δ*_H_ 2.88), H-2 (*δ*_H_ 5.02)/H_3_-18 (*δ*_H_ 1.10), H-2 (*δ*_H_ 5.02)/H_3_-19 (*δ*_H_ 1.07) and the absence of ROESY correlation between H-3 (*δ*_H_ 2.49)/H_3_-18 (*δ*_H_ 1.10), H-2 (*δ*_H_ 5.02)/H-7 (*δ*_H_ 2.88), H-2 (*δ*_H_ 5.02)/H-3 (*δ*_H_ 2.49) indicated that H-3 and H-7 were at the same side and arbitrarily assigned as being in *α*-configuration, whereas H_3_-18 and H_3_-19 were assigned as being in *β*-configuration (Figure 3). The ROESY experiment did not allow for determining the relative configuration of C-8, C-11 and C-12 due to the presence of a highly flexible macrocycle. As a result, QM-NMR employing DP4+ probability analysis was carried out. This is a potent tool whose application for capnosane-type diterpenes has been proven to be reliable [3]. QM-NMR calculations on eight potential isomers **1a**–**1h** (Figure 4) were then performed. According to the experimental results, the NMR data of compound **1** offered the highest degree of matching for **1g** (3S*, 4S*, 7S*, 8S*, 11S*, 12S*) (DP4+ probability 99.81%). Therefore, the 8S* configuration was consistent with the correlations observed in the ROESY spectrum. Finally, the structure of compound **1** was determined by the analysis presented above and represented in Figure 1.

**Figure 5:**
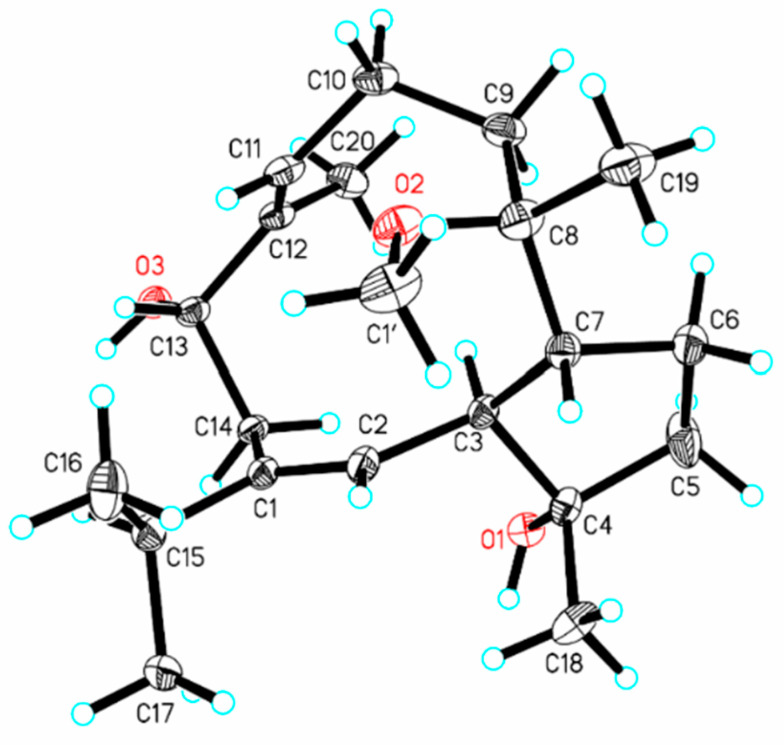
Single-crystal X-ray structure of **2** (the ellipsoids are shown at a 30% probability level).

Sarcocrassolin B (**2**), isolated as a colorless crystal, had a molecular formula of C_21_H_36_O_3_ according to its HRESIMS data at *m*/*z* 695.5211 (calculated as 695.5221 for C_42_H_72_O_6_Na, [2M + Na]^+^), with four degrees of unsaturation. Additionally, the characteristic infrared absorption bands at 3421 and 1602 cm^−1^ provided evidence for the presence of hydroxyl and olefinic groups. The ^1^H NMR data revealed the signals of five methyl groups (*δ*_H_ 1.06, 1.07, 1.08, 1.15, 1.69), one methoxy group (*δ*_H_ 3.12) and two olefinic protons (*δ*_H_ 5.00, 5.38). The ^13^C NMR data showed the presence of 21 signals, which comprised two pairs of olefinic carbons, five methyl signals, five methylene signals, four sp^3^ methines, two sp^3^ quaternary carbons and one methoxy carbon. Based on a comprehensive analysis of its NMR spectra, compound **2** almost resembled trocheliophol G, a capnosane-type macrocyclic diterpene from *S. trocheliophorum* [6]. The major difference was observed at the C-13 and C-14 positions, where a hydroxy at the C-13 position in **2** shifted to the C-14 position in trocheliophol G. The significant HMBC cross peaks from H_3_-20 (*δ*_H_ 1.69) to C-11 (*δ*_C_ 127.9)/C-12 (*δ*_C_ 134.3)/C-13 (*δ*_C_ 76.2), and from H-2 (*δ*_H_ 5.00) to C-14 (*δ*_C_ 38.3) as well as the ^1^H-^1^H COSY cross peaks of H-13 (*δ*_H_ 3.97)/H-14a (*δ*_H_ 1.97) and H-13 (*δ*_H_ 3.97)/H-14b (*δ*_H_ 2.80), further supported this conclusion. The ROESY spectrum verified the *E* configurations of the double-bonds *Δ*^1^ and *Δ*^11^, with the essential cross peaks of H-2 (*δ*_H_ 5.00)/H_3_-16 (*δ*_H_ 1.08) and the lack of H-11 (*δ*_H_ 5.38)/H_3_-20 (*δ*_H_ 1.69). In addition, the key ROESY cross peaks of H-2 (*δ*_H_ 5.00)/H_3_-18 (*δ*_H_ 1.06) and H-2 (*δ*_H_ 5.00)/H-7 (*δ*_H_ 2.15), along with the lack of cross peak of H-2 (*δ*_H_ 5.00)/H-3 (*δ*_H_ 2.34) indicated that H-7 and H_3_-18 were co-facial, while H-3 was on the opposite side of the molecule (Figure 3). The relative configuration of C-8 and C-13 could not be established from the ROESY experiment either. However, an appropriate single crystal of compound **2** was obtained in a solution of MeOH/H_2_O (20:1). Ultimately, as shown in Figure 5, the absolute configuration of **2** was obtained by analyzing the X-ray crystallographic data (Flack parameter: −0.3(4)) and was unambiguously established as 3*S*, 4*S*, 7*R*, 8*R*, 13*S*.

Sarcocrassolin C (**3**) was isolated as a colorless oil and assigned the molecular formula C_22_H_40_O_4_, identical to that of **1**, according to the HRESIMS ion peak at *m*/*z* 391.2819 (calculated as 391.2819 for C_22_H_40_O_4_Na, [M + Na]^+^), with three degrees of unsaturation. The characteristic infrared absorption bands at 3431 and 1632 cm^−1^ indicated the presence of hydroxy and olefinic groups, respectively. By comparing the ^1^H NMR and ^13^C NMR data of compounds **3** and **1**, we found that compound **3** was highly similar to **1**. Following that, the analysis of NMR data of compound **3** implied that compounds **3** and **1** shared the same planar structure. The main difference was that H-7 upfield-shifted significantly from *δ*_H_ 2.88 in **1** to *δ*_H_ 1.97 in **3**. Meanwhile, the higher value of *J*_H-3/H-7_ (11.5 Hz) in **3** compared to *J*_H-3/H-7_ (5.3 Hz) in **1** indicated that **3** and **1** exhibited different configurations at C-7. The ROESY cross peak between H-7 (*δ*_H_ 1.97) and H_3_-18 (*δ*_H_ 1.06) together with the lack of ROESY correlation between H-3 (*δ*_H_ 2.36) and H-7 (*δ*_H_ 1.97) also validated this hypothesis. The double-bond *Δ*^1^ in **3** possessed *E*-geometry, according to the ROESY cross peaks between H-2 (*δ*_H_ 5.16) and H_3_-16 (*δ*_H_ 1.10). The correlations of H-2 (*δ*_H_ 5.16)/H_3_-18 (*δ*_H_ 1.06) and H-2 (*δ*_H_ 5.16)/H-7 (*δ*_H_ 1.97) showed in ROESY spectrum, whereas no cross peaks of H-3 (*δ*_H_ 2.36)/H_3_-18 (*δ*_H_ 1.06) and H-2 (*δ*_H_ 5.16)/H-3 (*δ*_H_ 2.36) were observed (Figure 3). This indicated that H_3_-18 and H-7 were co-facial, whereas H-3 was on the opposite side of the molecule. The identical key ROESY correlations between compounds **3** and **2** indicated that the relative configuration of C-3, C-4 and C-7 in compounds **3** and **2** could be the same and established as 3S*, 4S*, 7R*, which was unambiguously defined by single-crystal X-ray diffraction in compound **2**. The ROESY experiment also failed to identify the relative configuration of C-8, C-11 and C-12. Therefore, eight potential isomers **3a**–**3h** (Figure S57) were calculated. The correlation coefficient R^2^= 0.9981 (DP4+ probability, 100%) indicated that the experimental NMR results of **3** were more consistent with **3c** (3S*, 4S*, 7R*, 8R*, 11S*, 12R*) than other isomers. Consequently, the relative configuration of **3** ended up being established as illustrated in Figure 1.

Sarcocrassolin D (**4**) was obtained as a colorless oil. Its molecular formula was established as C_21_H_38_O_4_ by HRESIMS data at *m*/*z* 731.5432 (calculated as 731.5432 for C_42_H_76_O_8_Na, [2M + Na]^+^). The analysis of the NMR data of **4** showed that **4** mostly resembled **3**. The major difference between **4** and **3** was at the C-8 position, where the methoxy group in **3** was substituted by a hydroxy group in **4**. This alteration was further validated by their 14-mass unit difference. The double-bond *Δ*^1^ in **4** was proposed to have *E*-geometry, in accordance with the ROESY cross peak between H-2 (*δ*_H_ 5.21) and H_3_-16 (*δ*_H_ 1.11). Furthermore, the relative configuration of C-3, C-4 and C-7 could be tentatively established as 3S*, 4S* and 7R*, respectively, based on the similar crucial ROESY correlation between **3** and **4** (Figure 3). Finally, eight potential isomers **4a**–**4h** (Figure S58) were calculated to explicate the spatial arrangement of C-11, C-12 and C-8. The correlation coefficient R^2^ = 0.9835 (DP4+ probability, 100%) suggested that **4e** (3S*, 4S*, 7R*, 8S*, 11R*, 12R*) was more compatible with the experimental NMR results. Consequently, the relative configuration of **4** was defined as shown in Figure 1.

The HRESIMS ion peak at *m*/*z* 345.2398 (calculated as 345.2400 for C_20_H_34_O_3_Na, [M + Na]^+^) revealed the molecular formula of sarcocrassolin E (**5**) to be C_20_H_34_O_3_ with four indexes of hydrogen deficit. The existence of hydroxy and olefinic groups could be determined by the characteristic infrared absorption bands at 3393 and 1650 cm^−1^, respectively. The ^13^C NMR spectrum revealed twenty carbon signals, which were classified by DEPT and HSQC spectra and then attributed to four quaternary carbons, five methines, six methylenes and five methyls. One double bond and one epoxy ring were composed of two methines, C-2 (*δ*_C_ 120.4, *δ*_H_ 5.31) and C-11 (*δ*_C_ 61.7, *δ*_H_ 3.16), as well as two quaternary carbons, C-1 (*δ*_C_ 146.5) and C-12 (*δ*_C_ 60.1), which contributed to the two indexes of hydrogen deficit. The remaining two indexes of hydrogen deficit thus revealed that the molecule contained two rings. Following that, the entire planar structure of **5** could be constructed using the 2D NMR data as depicted in Figure 2. When the NMR data were further analyzed, it became clear that compound **5** and sarcophytrol B [7], a capnosane-type macrocyclic diterpene that had previously been identified, were highly similar. The major difference was the configuration of double-bond *Δ*^1^. In fact, that had an *E* configuration in **5** and *Z* configuration in sarcophytrol B, which was established by the ROESY cross peaks between H-2 and H_3_-16 in compound **5**. It was also possible to establish the relative configurations of C-3, C-4 and C-7 as 3S*, 4S* and 7R*, respectively, by the similar key ROESY cross peaks observed in compounds **4** and **5**. Consequently, four possible isomers **5a**–**5d** (Figure S59) were calculated. Based on the experimental results, the NMR data of compound **5** provided the highest degree of matching for **5d** (3S*, 4S*, 7R*, 8S*, 11S*, 12R*), with 100% probabilities. Consequently, the relative configuration of **5** was defined as 3S*, 4S*, 7R*, 8S*, 11S* and 12R* (Figure 1).

Sarcocrassolin F (**6**), isolated as a colorless oil, had a molecular formula C_21_H_36_O_3_ based on the HRESIMS data at *m*/*z* 359.2557 (calculated as 359.2557 for C_21_H_36_O_3_Na, [M + Na]^+^). The analysis of its ^1^H and ^13^C NMR spectral characteristics data showed that **6** displayed extensive resemblance to **1**, except for the existence of an exocyclic double bond at C-8, C-19 in **6** instead of a methoxy and a methyl at C-8 in **1**, which was validated by key HMBC cross peaks from H-19 to C-7/C-8/C-9. According to the noticeable ROESY cross peak of H-2/H_3_-16, the double-bond *Δ*^1^ in **6** was proven to be of the *E*-geometry. The ROESY cross peak between H-2 and H_3_-18, along with the lack of cross peaks of H-2/H-3 and H-3/H_3_-18, indicated that H-3 and H_3_-18 were oriented on the opposite side of the molecule. Due to the insufficient signals, ROESY correlations were unable to ascertain the relative configuration of C-7. However, the nearly identical coupling constant of *J*_H-3/H-7_ (5.9 Hz) in **6** and *J*_H-3/H-7_ (5.3 Hz) in **1** revealed that H-3 and H-7 had the same orientation in both molecules. Finally, the QM-NMR using DP4+ probability analysis was carried out for the four potential isomers **6a**–**6d** (Figure S60) to explicate the spatial arrangement of C-11 and C-12. The experimental data indicated that **6d** (3S*, 4S*, 7S*, 11S*, 12R*) was more consistent with the correlation coefficient R^2^ = 0.9975 (DP4 + probability, 100%). Consequently, the relative configuration of **6** was depicted as drawn in Figure 1.

Compound **7** was identified as pavidolide D on the basis of the MS and NMR data analyses and comparison with the NMR data from a previously reported article [14].

**Scheme 1:**
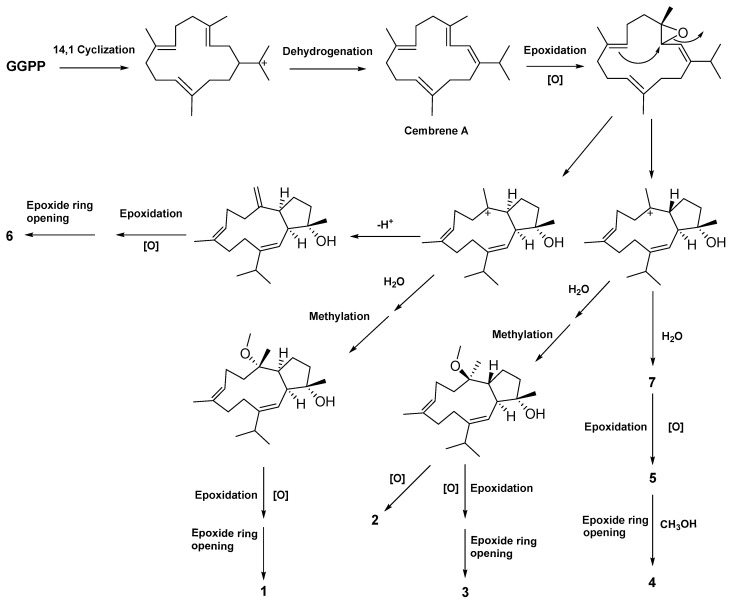
Postulated biosynthetic pathway of compounds **1**–**7**.

Because all compounds **1**–**7** possessed the same 5/11-fused bicyclic carbon skeleton and were obtained from the same animal material, it was reasonable to assume that they originated from the same biosynthetic pathway. According to their structural characteristics, a potential biosynthetic pathway of compounds **1**–**7** has been postulated (Scheme 1). The precursor geranylgeranyl diphosphate (GGPP) via 14,1 cyclization and dehydrogenation generates intermediate cembrene A. Subsequently, the 5/11-fused bicyclic carbon skeleton was generated by epoxidation and transannular cyclization from cembrene A. Finally, all compounds **1**–**7** were obtained after undergoing a series of reactions (such as methylation, epoxidation and dehydrogenation).

### 2.2. In Vitro Biological Assay

In biological assays, all the abovementioned compounds were tested for their cytotoxic and anti-inflammatory activities. In cytotoxicity screening assay, all compounds were assayed against five cell lines: SGC-7901 (human gastric cells), K562 (human myeloid leukemia cells), BEL-7402 (human hepatocellular carcinoma cells), HeLa (human cervical carcinoma cells) and A549 (human lung cancer cells). Unfortunately, none of these compounds displayed obvious cytotoxic activity at a concentration of 20.0 μg/mL. In anti-inflammatory testing, compounds **4** and **5** exhibited weak inhibitory activity of NO release in LPS-stimulated RAW264.7 cells, with the IC_50_ values of 76.8 ± 8.0 μM and 93.0 ± 3.8 μM, respectively (Table 3). The positive control used was quercetin, which had an IC_50_ value of 12.7 ± 2.4 μM. The cell viability of RAW264.7 cells upon treatment with the compounds was also determined by the MTT method, and the result indicated that compounds **3** and **4** had no cytotoxicity against RAW264.7 cells at a concentration of 100.0 μM.

## 3. Materials and Methods

### 3.1. General Experimental Procedures

Optical rotation was performed on a Modular Circular Polarimeter (Anton Paar, Graz, Austria). The IR spectra were recorded on a Nicolet 380 infrared spectrometer (Thermo Electron Corporation, Madison, WI, USA). UV and CD data were collected on a MOS-500 spectrometer (Biologic, Seyssinet-Pariset, France). NMR spectra were obtained on a Bruker AV-500 NMR spectrometer (Bruker, Bremen, Germany), using the TMS as an internal standard. HRESIMS spectra were measured on an API QSTAR Pulsar mass spectrometer (Bruker, Bremen, Germany). Analytical HPLC was recorded on an Agilent Technologies 1260 Infinity II with an Agilent DAD G1315D detector (Agilent, Palo Alto, CA, USA). Semi-preparative HPLC was performed on an ODS column (COSMOSIL-packed C_18_, 5 mm, 10 mm × 250 mm). Column chromatography was performed on a Sephadex LH-20 (Merck, Darmstadt, Germany) and Silica gel (60–80, 200–300 and 300–400 mesh, Qingdao Marine Chemical Co., Ltd., Qingdao, China). Analytical TLC was conducted on precoated silica gel GF254 plates (Qingdao Marine Chemical Co., Ltd., Qingdao, China), and the spots were detected by spraying with 10% H_2_SO_4_ in EtOH and subsequent heating.

### 3.2. Animal Materials

The soft coral *S. crassocaule* was collected in October 2018 at a depth of −20 m off the Nansha Islands, Hainan Province, China. The fresh sample was immediately frozen. The animal material was identified by Prof. X.-B. Li (Hainan University). A voucher specimen (No. 18-NS-10) was deposited at the Institute of Tropical Bioscience and Biotechnology, Chinese Academy of Tropical Agricultural Sciences.

### 3.3. Extraction and Isolation

The frozen soft coral *S. crassocaule* (84.0 g, dry weight) was chopped into pieces and extracted with EtOH after sonication at room temperature (5 × 1.5 L). A brown residue was yielded after the organic extract was evaporated in vacuo and then partitioned between EtOAc and water. The EtOAc phase was evaporated in vacuo to yield a deep brown residue (8.0 g), which was then fractionated using gradient silica gel column chromatography (silica gel CC) (300–400 mesh) with 0 → 100% EtOAc in petroleum ether (PE), resulting in twenty-one fractions (A–U). Fraction U (814.0 mg) was separated into seven subfractions, U1–U7, using Sephadex LH-20 column chromatography (Sephadex LH-20 CC) eluted with 100% MeOH. Fraction U4 (242.0 mg) was separated using silica gel CC eluted with CH_2_Cl_2_/MeOH (60:1, 40:1) and subsequently purified using semi-preparative HPLC eluted with 75% aqueous MeOH (flow rate, 3.0 mL/min) to obtain compound **1** (1.4 mg, t*_R_* = 15.0 min). Fractions S (1.8 g), T (120.0 mg), FG (384.0 mg), HI (405.0 mg) and M (707.0 mg) were all subjected to the same separation process of Sephadex LH-20 CC or silica gel CC as aforementioned to give a series of subfractions. Subfractions S5F (18.2 mg) and S7F2 (8.0 mg) were purified by semi-preparative HPLC eluted with 70% and 75% aqueous MeOH, respectively, to obtain compounds **3** (7.4 mg, t*_R_* = 17.7 min) and **2** (3.7 mg t*_R_* = 15.9 min). Subfraction T2 (48.2 mg) was separated by silica gel CC using the ratios of CH_2_Cl_2_/MeOH (150:1, 90:1, 60:1) to yield compound **4** (2.7 mg). Subfractions T3B (11.5 mg), HI5E1 (9.4 mg) and M4D2 (5.6 mg) were purified by semi-preparative HPLC eluted with 70% (MeOH/H_2_O), 70% (MeCN/H_2_O) and 75% (MeOH/H_2_O), respectively, to obtain compounds **5** (1.5 mg, t*_R_* = 9.2 min), **6** (3.9 mg, t*_R_* = 9.8 min) and **7** (3.2 mg, t*_R_* = 13.7 min).

Sarcocrassolin A (**1**): White powder. ${\left[\alpha \right]}_{D}^{25}$= −78 (*c* 0.10, MeOH); UV (MeOH) *λ*_max_ (log*ε*): 207 (3.22) nm; IR (KBr) *v*_max_ (cm^−1^): 3418, 2958, 1638, 1462, 1377, 1071, 919. ^1^H and ^13^C NMR data, see Table 1 and Table 2; HRESIMS [M + Na]^+^ *m/z* 391.2820 (calcd. for C_22_H_40_O_4_Na, 391.2819).

Sarcocrassolin B (**2**): Colorless crystals. M.p. 80–81 °C. ${\left[\alpha \right]}_{D}^{25}$= −94 (*c* 0.10, MeOH); UV (MeOH) *λ*_max_ (log*ε*): 212 (3.34) nm; IR (KBr) *v*_max_ (cm^−1^): 3421, 2960, 2929, 1602, 1457, 1377, 1267, 1126, 1086. ^1^H NMR and ^13^C NMR data, see Table 1 and Table 2; HRESIMS *m/z* 695.5211 [2M + Na]^+^ (calcd. for C_42_H_72_O_6_Na, 695.5221).

Sarcocrassolin C (**3**): Colorless oil. ${\left[\alpha \right]}_{D}^{25}$= −82 (*c* 0.10, MeOH); UV (MeOH) λmax (logε): 208 (3.25) nm; IR (KBr) *v*_max_ (cm^−1^): 3431, 2958, 1632, 1461, 1376, 1085. ^1^H and ^13^C NMR data, see Table 1 and Table 2; HRESIMS [M + Na] ^+^ *m/z* 391.2819 (calcd. for C_22_H_40_O_4_Na, 391.2819).

Sarcocrassolin D (**4**): Colorless oil. ${\left[\alpha \right]}_{D}^{25}$= −38 (*c* 0.10, MeOH); UV (MeOH) λmax (logε): 207 (3.15) nm; IR (KBr) *v*_max_ (cm^−1^): 3430, 2960, 1649, 1461, 1375, 1080. ^1^H and ^13^C NMR data, see Table 1 and Table 2; HRESIMS [2M + Na] ^+^ *m/z* 731.5432 (calcd. for C_42_H_76_O_8_Na, 731.5432).

Sarcocrassolin E (**5**): Colorless oil. ${\left[\alpha \right]}_{D}^{25}$= +6 (*c* 0.10, MeOH); UV (MeOH) λmax (logε): 209 (3.12) nm; IR (KBr) *v*_max_ (cm^−1^): 3393, 2962, 1650, 1589, 1461, 1377, 1087. ^1^H and ^13^C NMR data, see Table 1 and Table 2; HRESIMS [M + Na] ^+^ *m/z* 345.2398 (calcd. for C_20_H_34_O_3_Na, 345.2400).

Sarcocrassolin F (**6**): Colorless oil. ${\left[\alpha \right]}_{D}^{25}$= −75 (*c* 0.10, MeOH); UV (MeOH) λmax (logε): 213 (3.20) nm; IR (KBr) *v*_max_ (cm^−1^): 3444, 2958, 1633, 1461, 1094. ^1^H and ^13^C NMR data, see Table 1 and Table 2; HRESIMS [M + Na] ^+^ *m/z* 359.2557 (calcd. for C_21_H_36_O_3_Na, 359.2557).

### 3.4. X-ray Crystallographic Analysis

The solvent methanol/H_2_O (20:1) was used to create an acceptable crystal of compound **2** (0.15 × 0.12 × 0.1 mm^3^) at ambient temperature. The crystallographic data were collected at 170 K on a diffractometer Rigaku Oxford Diffraction Supernova Dual Source, Cu at Zero, equipped with an AtlasS2 CCD using Cu Kα radiation (λ = 1.54184 Å) by using a w scan mode. CrysAlisPro (version: 1.171.38.41) was used to process the data, and the structures were solved using direct methods with the SHELXT structure solution program via intrinsic phasing algorithm using Olex2 software (version: 1.5). The non-hydrogen atoms in the trial structure were detected and then refined anisotropically with SHELXL-2018 using a full-matrix least-squares program based on *F^2^*; *F^2^* was used to obtain the weighted *R* factor, *wR* and goodness-of-fit *S* values. The placements of the hydrogen atoms were fixed geometrically at the calculated distances and permitted to ride on the parent atoms. Detailed crystallographic data are provided in the Supplementary Materials (Table S11) and have been deposited at the Cambridge Crystallographic Data Centre for inspection (deposition numbers: CCDC 2291468 for **2**).

Crystal data for compound **2**: C_21_H_37_O_3.5_, *M* = 345.50, orthorhombic system, space group P2_1_2_1_2_1_/c (no. 19), *a* = 9.5531(6) Å, *b* = 15.2772(15) Å, *c* = 28.949(5) Å, *β* = 90°, *V* = 4224.9(8) Å^3^, *Z* = 8, *T* = 169.99(10) K, μ(Cu Kα) = 0.564 mm^−1^, *Dcalc* = 1.086 g/cm^3^, 16,223 reflections measured (8.416° ≤ 2Θ ≤ 147.908°), 8320 unique (*R*_int_ = 0.1057, *R*_sigma_ = 0.2540) which were used in all calculations. The final *R*_1_ was 0.0784 (I > 2σ(I)), and *wR*_2_ was 0.2254 (all data).

### 3.5. Computational Details

The torsional sampling (MCMM) method and the OPLS_2005 force field were used for carrying out a conformational search inside a 21 kJ/mol energy window. The conformers were reoptimized using the IEFPCM solvent model for chloroform at the B3LYP/6-31G(d) level for conformers over 1% Boltzmann populations. The reoptimized geometries’ position at the energy minima was further verified by frequency analysis. Then, as advised for DP4+, NMR calculations were carried out at the PCM/mPW1PW91/6-311+G(d,p) level. The GIAO method was used to compute NMR shielding constants. After obtaining a Boltzmann distribution for each stereoisomer, shielding constants were averaged and correlated with the experimental results.

### 3.6. Cytotoxic Detection

The cytotoxic activity bioassay of all the abovementioned compounds was carried out as described in our previous paper [25]. Cisplatin was used as a positive control.

### 3.7. Anti-Inflammatory Assay

In the bioassay for anti-inflammation, all the abovementioned compounds were assayed for the inhibition of NO released in lipopolysaccharide-stimulated RAW264.7 cells (bought from the Stem Cell Bank of the Chinese Academy of Sciences) by the Griess method [26]. The mouse macrophage cells were cultured in 96-well plates at a concentration of 5 × 10^4^ cells per mL (100 μL each well) in a humidified incubator of 5% CO_2_ at 37 °C for 24 h. The transfected cells were then stimulated using LPS (50 μL, 500 ng/mL) for 24 h after being treated with 50 μL solution of the tested compound at different concentrations (100.0, 50.0, 25.0, 12.5 μM) for 1 h. Then, 100 μL of supernatants were taken from each well, and 100 μL of Griess reagent (40 mg/mL) was added into a new 96-well plate. Finally, using a microplate reader, the absorbance of each well was measured at the wavelength of 540 nm. The IC_50_ was calculated using GraphPad Prism 9.0. The positive control employed was quercetin. The cytotoxicity of compounds against RAW264.7 cells was determined by the MTT method.

## 4. Conclusions

In conclusion, six uncommon capnosane-type macrocyclic diterpenes, sarcocrassolins A–F (**1**–**6**), and one related known analog pavidolide D (**7**) were isolated from the soft coral *S. crassocaule* collected off the Nansha Islands. Due to the presence of a highly flexible macrocycle, the configuration assignments of capnosane-type macrocyclic diterpenes are still confronted with huge challenges. In our continuing efforts, the absolute configuration of compound **2** was assigned based on an X-ray crystallography study, and the relative configurations of the remaining compounds were established through QM-NMR using DP4+ probability analysis. Despite the fact that the soft coral *S. crassocaule* has been acknowledged as a major source of cembrane-type diterpenes, the capnosane-type macrocyclic diterpenes have not been previously reported. This is the first time that capnosane-type macrocyclic diterpenes were described in the soft coral *S. crassocaule*. Consequently, the soft coral *S. crassocaule* could be a potential source of structurally diverse capnosane-type macrocyclic diterpenes. In anti-inflammatory testing, all isolated compounds were assayed for the inhibitory activity on NO release in LPS-induced RAW264.7 cells. Sarcocrassolin D (**4**) and sarcocrassolin E (**5**) exhibited weak inhibitory effects. Furthermore, all compounds were tested for cytotoxicity. Unfortunately, none of the compounds demonstrated notable activity.

## Figures and Tables

**Table 1:** ^1^H NMR data for compounds **1**–**6** (500 MHz, CDCl_3_).

No.	1	2	3	4	5	6
2	5.02 d (11.7)	5.00 d (9.3)	5.16 d (10.3)	5.21 d (10.4)	5.31 d (12.0)	4.88 d (10.7)
3	2.49 dd (11.7, 5.3)	2.34 t (9.3)	2.36 dd (11.5, 10.3)	2.41 t (10.9)	2.97 t (11.5)	2.41 dd (10.7, 5.9)
5	1.77 m 1.66 m	1.58–1.66 m	1.74 m 1.69 m	1.74 m	1.79 m	1.79 m 1.69 m
6	1.86 m 1.75 m	1.59 m 1.48 m	1.68 m 1.48 m	1.72 m 1.50 m	1.97 m 1.72 m	1.85–1.95 m
7	2.88 m	2.15 m	1.97 m	1.93 m	2.73 m	2.17 m
9	1.97 m 1.62 m	2.00 m 1.55 m	1.74 m 1.58 ddd (15.5, 6.7, 2.2)	1.69 m	1.99 m 1.73 m	2.27 m 2.16 m
10	1.56 m 1.32 m	2.08–2.19 m	1.90 m 1.30 m	1.67 m 1.41 m	2.14 m 1.22 m	1.58 m 1.45 m
11	3.59 t (11.0)	5.38 t (7.7)	3.66 t (10.1)	3.68 t (9.5)	3.16 dd (10.0, 4.3)	3.60 t (9.3)
13	2.15 m 1.19 m	3.97 dd (11.0, 2.2)	2.10 m 1.21 m	2.14 m 1.24 m	2.07 m 1.72 m	2.09 m 1.15 m
14	2.09 m 1.97 m	2.81 t (11.9) 1.98 m	2.13 m 1.94 m	2.18 m 2.00 m	2.60 td (13.3, 6.3) 1.97 m	1.99 m 1.89 m
15	2.23 m	2.26 sept (6.8)	2.22 m	2.28 m	2.37 sept (6.8)	2.22 m
16	1.08 d (6.8)	1.08 d (6.8)	1.10 d (6.8)	1.11 d (6.8)	1.14 d (6.8)	1.06 d (6.8)
17	0.99 d (6.8)	1.07 d (6.8)	1.02 d (6.8)	1.05 d (6.8)	1.05 d (6.8)	0.98 d (6.8)
18	1.10 s	1.06 s	1.06 s	1.08 s	1.16 s	1.14 s
19	1.07 s	1.15 s	1.08 s	1.16 s	1.24 s	5.12 br s 4.89 br s
20	1.26 s	1.69 s	1.25 s	1.27 s	1.17 s	1.24 s
8-OMe	3.19 s	3.12 s	3.10 s			
12-OMe	3.22 s		3.20 s	3.21 s		3.21 s

**Table 2:** ^13^C NMR data for compounds **1**–**6** (125 MHz, CDCl_3_).

No.	1	2	3	4	5	6
1	146.7, C	141.6, C	146.8, C	151.0, C	146.5, C	146.6, C
2	121.0, CH	127.2, CH	122.7, CH	121.4, CH	120.4, CH	123.0, CH
3	52.0, CH	50.0, CH	51.6, CH	51.7, CH	50.0, CH	56.9, CH
4	83.2, C	82.4, C	81.4, C	81.6, C	82.2, C	82.6, C
5	37.5, CH_2_	39.5, CH_2_	37.7, CH_2_	38.4, CH_2_	39.6, CH_2_	40.7, CH_2_
6	23.1, CH_2_	24.8, CH_2_	24.7, CH_2_	25.0, CH_2_	25.3, CH_2_	31.7, CH_2_
7	50.0, CH	51.0, CH	52.7, CH	55.7, CH	48.5, CH	51.5, CH
8	79.3, C	78.0, C	80.0, C	75.8, C	74.5, C	154.3, C
9	35.7, CH_2_	36.2, CH_2_	30.4, CH_2_	33.4, CH_2_	39.2, CH_2_	34.2, CH_2_
10	25.1, CH_2_	23.0, CH_2_	24.9, CH_2_	25.6, CH_2_	23.4, CH_2_	28.8, CH_2_
11	78.7, CH	127.9, CH	78.9, CH	78.4, CH	61.7, CH	74.3, CH
12	79.4, C	134.3, C	79.4, C	79.3, C	60.1, C	79.7, C
13	33.2, CH_2_	76.2, CH	33.7, CH_2_	33.7, CH_2_	33.9, CH_2_	33.5, CH_2_
14	21.3, CH_2_	38.3, CH_2_	21.6, CH_2_	21.7, CH_2_	25.0, CH_2_	21.9, CH_2_
15	32.8, CH	34.5, CH	32.9, CH	33.0, CH	31.3, CH	32.1, CH
16	21.6, CH_3_	22.2, CH_3_	22.1, CH_3_	22.1, CH_3_	20.2, CH_3_	21.8, CH_3_
17	23.1, CH_3_	24.3, CH_3_	24.0, CH_3_	23.8, CH_3_	24.4, CH_3_	23.4, CH_3_
18	26.5, CH_3_	24.9, CH_3_	24.9, CH_3_	24.9, CH_3_	24.3, CH_3_	25.1, CH_3_
19	19.2, CH_3_	24.9, CH_3_	24.1, CH_3_	30.2, CH_3_	25.9, CH_3_	111.5, CH_2_
20	19.1, CH_3_	11.7, CH_3_	19.0, CH_3_	19.0, CH_3_	20.9, CH_3_	19.0, CH_3_
8-OMe	48.1, CH_3_	48.8, CH_3_	49.4, CH_3_			
12-OMe	49.5, CH_3_		49.5, CH_3_	49.5, CH_3_		49.5, CH_3_

**Table 3:** Anti-inflammatory effect of compounds **1**–**7**.

Compounds	IC_50_ ± SD (μM)	Compounds	IC_50_ ± SD (μM)
**1**	>100	**5**	93.0 ± 3.8
**2**	>100	**6**	>100
**3**	>100	**7**	>100
4	76.8 ± 8.0	Quercetin	12.7 ± 2.4

## Data Availability

The authors declare that all relevant data supporting the results of this study are available within the article and its Supplementary Materials file, or from the corresponding authors upon request.

## Supplementary Materials

The following supporting information can be downloaded at: https://www.mdpi.com/article/10.3390/md21120645/s1. Figures S1–S56: 1D, 2D NMR, MS, UV and IR spectra of compounds **1**–**7**, Figures S57–S60: Structures of isomers of compounds **3**–**6**, Tables S1–S10 and Figure S61: QM-NMR data, Table S11: Crystal data and structure refinement for compound **2**.
