# Supplementary material for: Six Undescribed Capnosane-Type Macrocyclic Diterpenoids from South China Sea Soft Coral Sarcophyton crassocaule: Structural Determination and Biological Evaluation
Source: Mar Drugs. 2023 Dec 18;21(12):645. doi: 10.3390/md21120645 (PMC10744964; doi:10.3390/md21120645)
Supplement: Supplementary file 1 [file marinedrugs-21-00645-s001.zip › marinedrugs-2749828-supplementary.pdf]

Table of contents

Figure S1. ^{1}H NMR spectrum (500 MHz) of compound **1** in $CDCl_3$

Figure S2. ^{13}C NMR spectrum (125 MHz) of compound **1** in $CDCl_3$

Figure S3. HSQC spectrum (500 MHz) of compound **1** in $CDCl_3$

Figure S4. ^{1}H-^{1}H COSY spectrum (500 MHz) of compound **1** in $CDCl_3$

Figure S5. HMBC spectrum (500 MHz) of compound **1** in $CDCl_3$

Figure S6. ROESY spectrum (500 MHz) of compound **1** in $CDCl_3$

Figure S7. IR spectrum of compound **1**

Figure S8. HR-ESIMS spectrum of compound **1**

Figure S9. UV spectrum of compound **1**

Figure S10. ^{1}H NMR spectrum (500 MHz) of compound **2** in $CDCl_3$

Figure S11. ^{13}C NMR spectrum (125 MHz) of compound **2** in $CDCl_3$

Figure S12. HSQC spectrum (500 MHz) of compound **2** in $CDCl_3$

Figure S13. ^{1}H-^{1}H COSY spectrum (500 MHz) of compound **2** in $CDCl_3$

Figure S14. HMBC spectrum (500 MHz) of compound **2** in $CDCl_3$

Figure S15. ROESY spectrum (500 MHz) of compound **2** in $CDCl_3$

Figure S16. IR spectrum of compound **2**

Figure S17. HR-ESIMS spectrum of compound **2**

Figure S18. UV spectrum of compound **2**

Figure S19. ^{1}H NMR spectrum (500 MHz) of compound **3** in $CDCl_3$

Figure S20. ^{13}C NMR spectrum (125 MHz) of compound **3** in $CDCl_3$

Figure S21. HSQC spectrum (500 MHz) of compound **3** in $CDCl_3$

Figure S22. ^{1}H-^{1}H COSY spectrum (500 MHz) of compound **3** in $CDCl_3$

Figure S23. HMBC spectrum (500 MHz) of compound **3** in $CDCl_3$

Figure S24. ROESY spectrum (500 MHz) of compound **3** in $CDCl_3$

Figure S25. IR spectrum of compound **3**

Figure S26. HR-ESIMS spectrum of compound **3**

Figure S27. UV spectrum of compound **3**

Figure S28. ^{1}H NMR spectrum (500 MHz) of compound **4** in $CDCl_3$

Figure S29. ^{13}C NMR spectrum (125 MHz) of compound **4** in $CDCl_3$

Figure S30. HSQC spectrum (500 MHz) of compound **4** in $CDCl_3$

Figure S31. ^{1}H-^{1}H COSY spectrum (500 MHz) of compound **4** in $CDCl_3$

Figure S32. HMBC spectrum (500 MHz) of compound **4** in $CDCl_3$

Figure S33. ROESY spectrum (500 MHz) of compound **4** in $CDCl_3$

Figure S34. IR spectrum of compound **4**

Figure S35. HR-ESIMS spectrum of compound **4**

Figure S36. UV spectrum of compound **4**

Figure S37. ^{1}H NMR spectrum (500 MHz) of compound **5** in $CDCl_3$

Figure S38. ^{13}C NMR spectrum (125 MHz) of compound **5** in $CDCl_3$

Figure S39. HSQC spectrum (500 MHz) of compound **5** in $CDCl_3$

Figure S40. ^{1}H-^{1}H COSY spectrum (500 MHz) of compound **5** in $CDCl_3$

Figure S41. HMBC spectrum (500 MHz) of compound **5** in $CDCl_3$

Figure S42. ROESY spectrum (500 MHz) of compound **5** in $CDCl_3$

**Figure S43.**	IR spectrum of compound **5**
**Figure S44.**	HR-ESIMS spectrum of compound **5**
**Figure S45.**	UV spectrum of compound **5**
**Figure S46.**	^{1}H NMR spectrum (500 MHz) of compound **6** in $CDCl_3$
**Figure S47.**	^{13}C NMR spectrum (125 MHz) of compound **6** in $CDCl_3$
**Figure S48.**	HSQC spectrum (500 MHz) of compound **6** in $CDCl_3$
**Figure S49.**	^{1}H-^{1}H COSY spectrum (500 MHz) of compound **6** in $CDCl_3$
**Figure S50.**	HMBC spectrum (500 MHz) of compound **6** in $CDCl_3$
**Figure S51.**	ROESY spectrum (500 MHz) of compound **6** in $CDCl_3$
**Figure S52.**	IR spectrum of compound **6**
**Figure S53.**	HR-ESIMS spectrum of compound **6**
**Figure S54.**	UV spectrum of compound **6**
**Figure S55.**	^{1}H NMR spectrum (500 MHz) of compound **7** in $CDCl_3$
**Figure S56.**	^{13}C NMR spectrum (125 MHz) of compound **7** in $CDCl_3$
**Figure S57.**	Structures of isomers of compounds **3**
**Figure S58.**	Structures of isomers of compounds **4**
**Figure S59.**	Structures of isomers of compounds **5**
**Figure S60.**	Structures of isomers of compounds **6**
**Table S1.**	Boltzmann averaged GIAO isotropic magnetic shielding constants (σ) of compound **1** calculated at the PCM/mPW1PW91/6-311G(d,p) //B3LYP/6-31G (d) level of theory
**Table S2.**	DP4+ results obtained using experimental data of compound **1** versus isomers **1a**—**1h**
**Table S3.**	Boltzmann averaged GIAO isotropic magnetic shielding constants (σ) of compound **3** calculated at the PCM/mPW1PW91/6-311G(d,p) //B3LYP/6-31G (d) level of theory
**Table S4.**	DP4+ results obtained using experimental data of compound **3** *versus* isomers **3a**—**3h**
**Table S5.**	Boltzmann averaged GIAO isotropic magnetic shielding constants (σ) of compound **4** calculated at the PCM/mPW1PW91/6-311G(d,p) //B3LYP/6-31G (d) level of theory
**Table S6.**	DP4+ results obtained using experimental data of compound **4** *versus* isomers **4a**—**4h**
**Table S7.**	Boltzmann averaged GIAO isotropic magnetic shielding constants (σ) of compound **5** calculated at the PCM/mPW1PW91/6-311G(d,p) //B3LYP/6-31G (d) level of theory
**Table S8.**	DP4+ results obtained using experimental data of compound **5** *versus* isomers **5a**—**5d**
**Table S9.**	Boltzmann averaged GIAO isotropic magnetic shielding constants (σ) of compound **6** calculated at the PCM/mPW1PW91/6-31G(d,p) //B3LYP/6-31G (d) level of theory
**Table S10.**	DP4+ results obtained using experimental data of compound **6** *versus* isomers **6a**—**6d**
**Figure S61.**	Correlation plots of compounds **1**, **3**—**6**
**Table S11.**	Crystal data and structure refinement for **2**

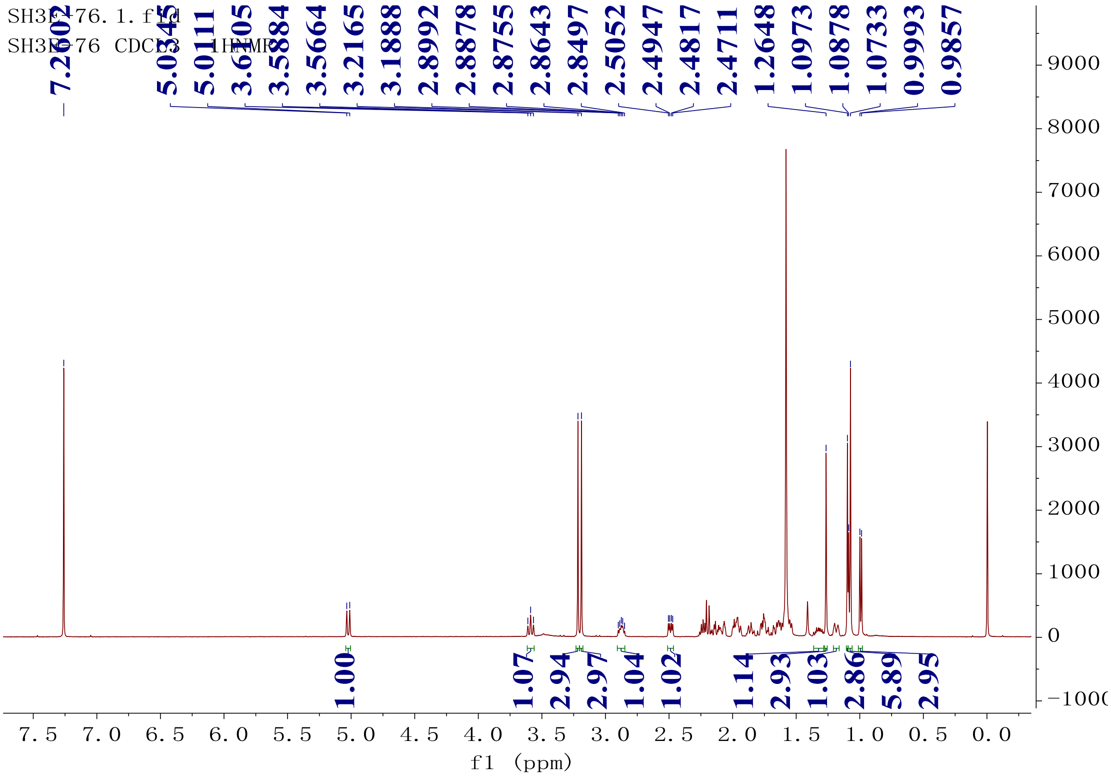

Figure S1. ^{1}H NMR spectrum (500 MHz) of compound **1** in $CDCl_3$

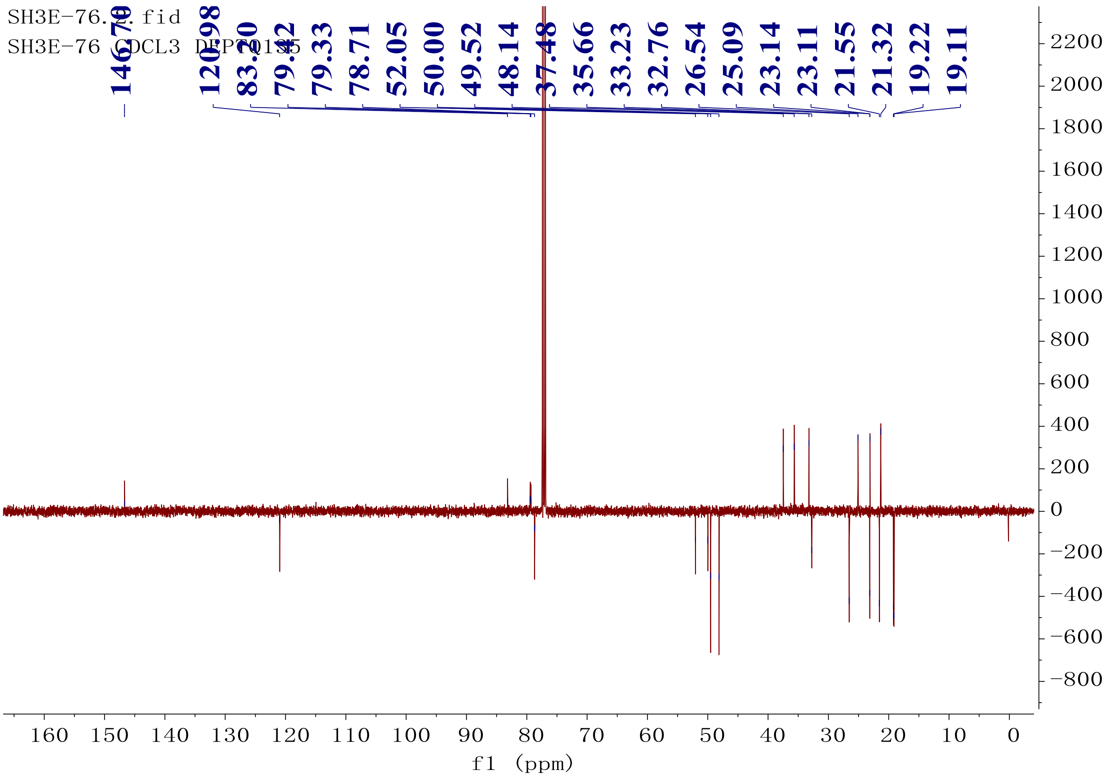

Figure S2. ^{13}C NMR spectrum (125 MHz) of compound **1** in CDCl3

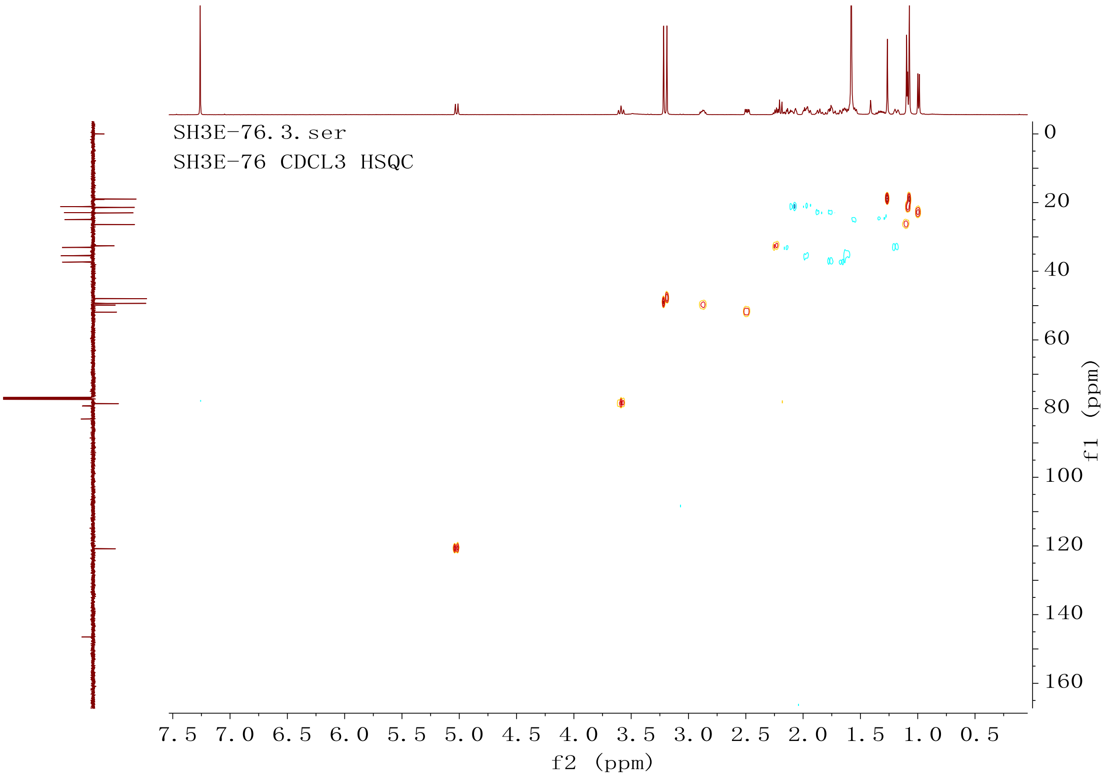

Figure S3. HSQC spectrum (500 MHz) of compound **1** in $CDCl_3$

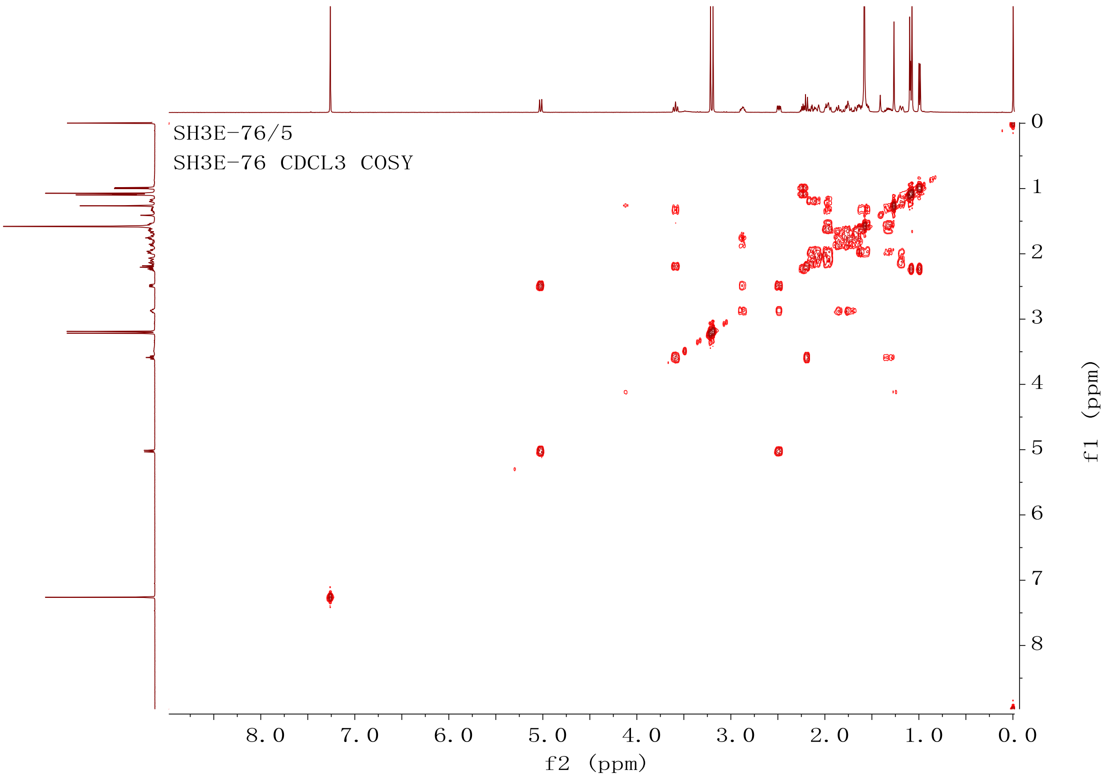

Figure S4. ^1H-^1H COSY spectrum (500 MHz) of compound **1** in $CDCl_3$

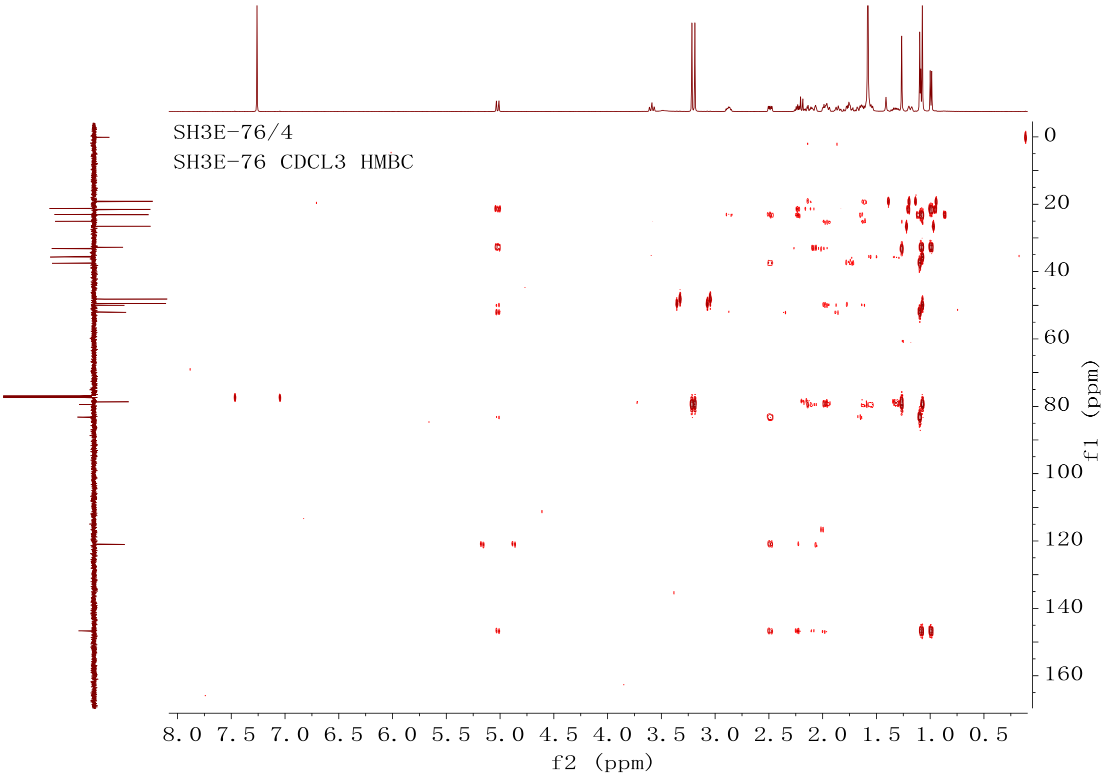

Figure S5. HMBC spectrum (500 MHz) of compound **1** in $CDCl_3$

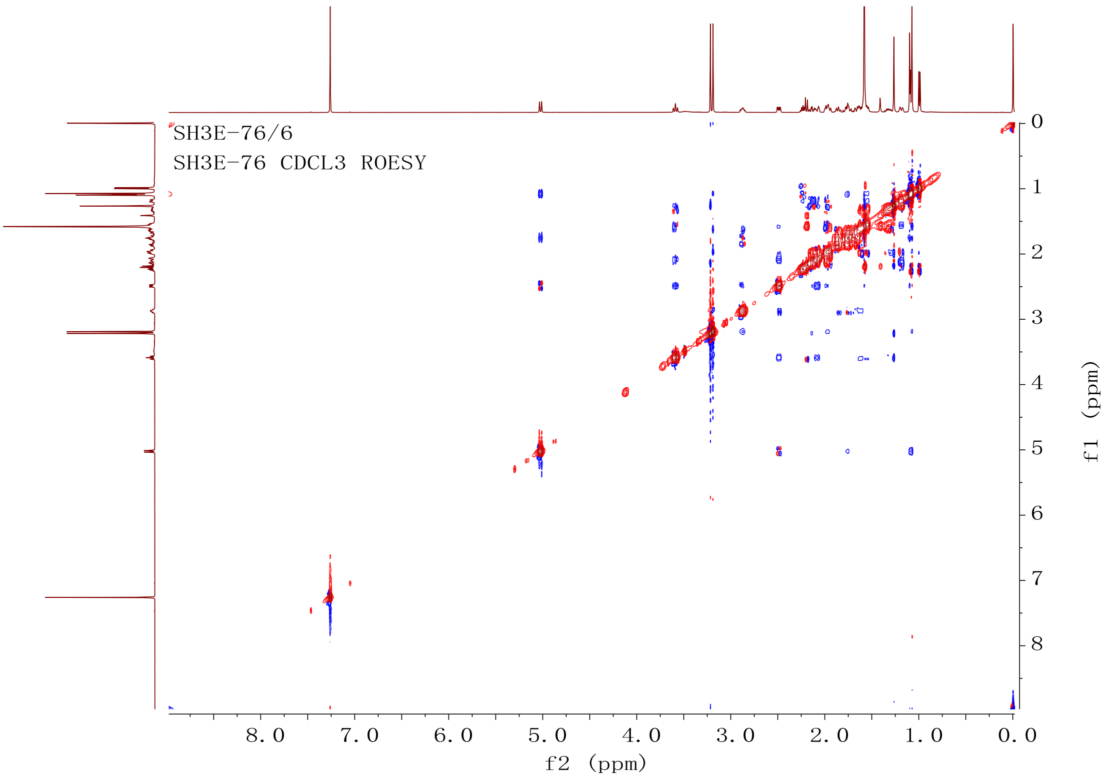

Figure S6. ROESY spectrum (500 MHz) of compound **1** in $CDCl_3$

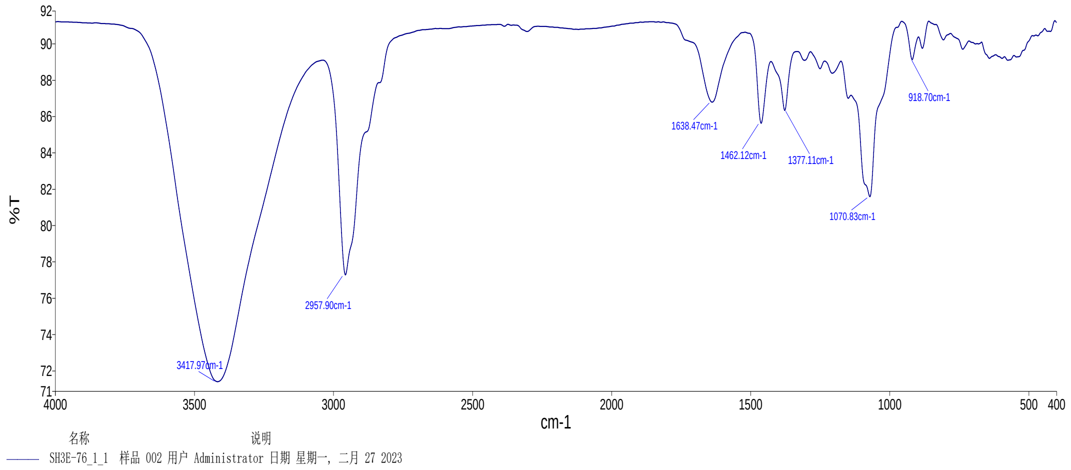

Figure S7. IR spectrum of compound **1**

Mass Spectrum SmartFormula Report

Analysis Info

Analysis Name	D:\2023\B502\20230920\SH3E-76_BA3_01_8716.d	Acquisition Date	2023-09-20 11:47:50
Method	lc-ms_as_ms-1min-20230519.m	Operator	Demo User
Sample Name	SH3E-76	Instrument	compact 8255754.20156
Comment			

Acquisition Parameter

Source Type	ESI	Ion Polarity	Positive	Set Nebulizer	1.8 Bar
Focus	Not active	Set Capillary	3500 V	Set Dry Heater	220 °C
Scan Begin	50 m/z	Set End Plate Offset	-500 V	Set Dry Gas	4.0 l/min
Scan End	2500 m/z	Set Charging Voltage	2000 V	Set Divert Valve	Waste
		Set Corona	0 nA	Set APCI Heater	0 °C

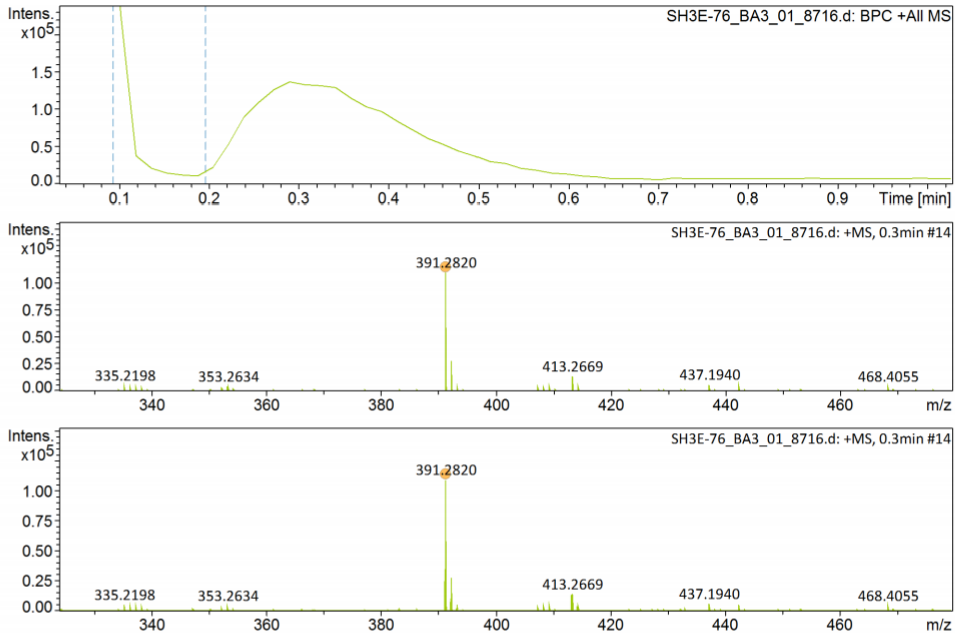

Meas. m/z	#	Ion Formula	m/z	err [ppm]	mSigma	# mSigma	Score	rdb	e⁻ Conf	N-Rule	Adduct
391.2820	1	C22H40NaO4	391.2819	-0.3	7.7	1	100.00	3.0	even	ok	M+Na

Figure S8. HR-ESIMS spectrum of compound **1**

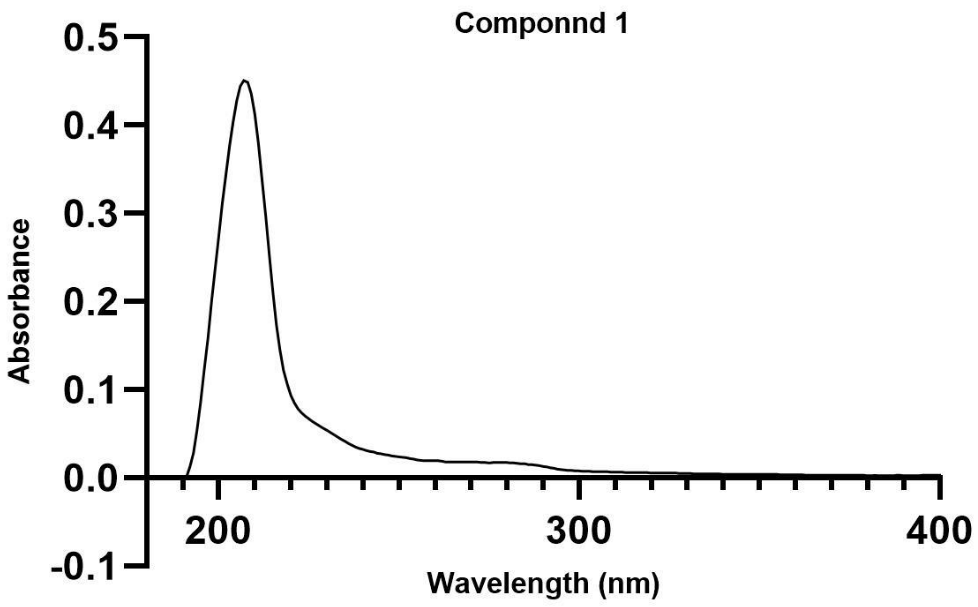

Figure S9. UV spectrum of compound **1**

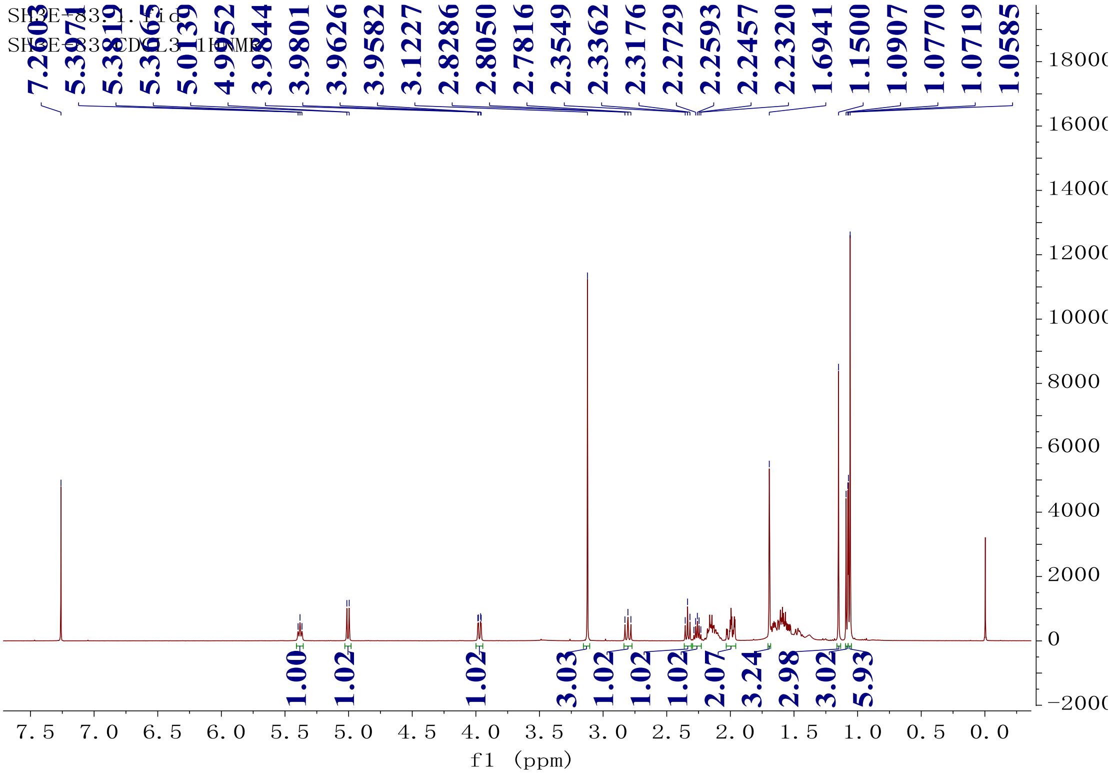

Figure S10. ^{1}H NMR spectrum (500 MHz) of compound **2** in $CDCl_3$

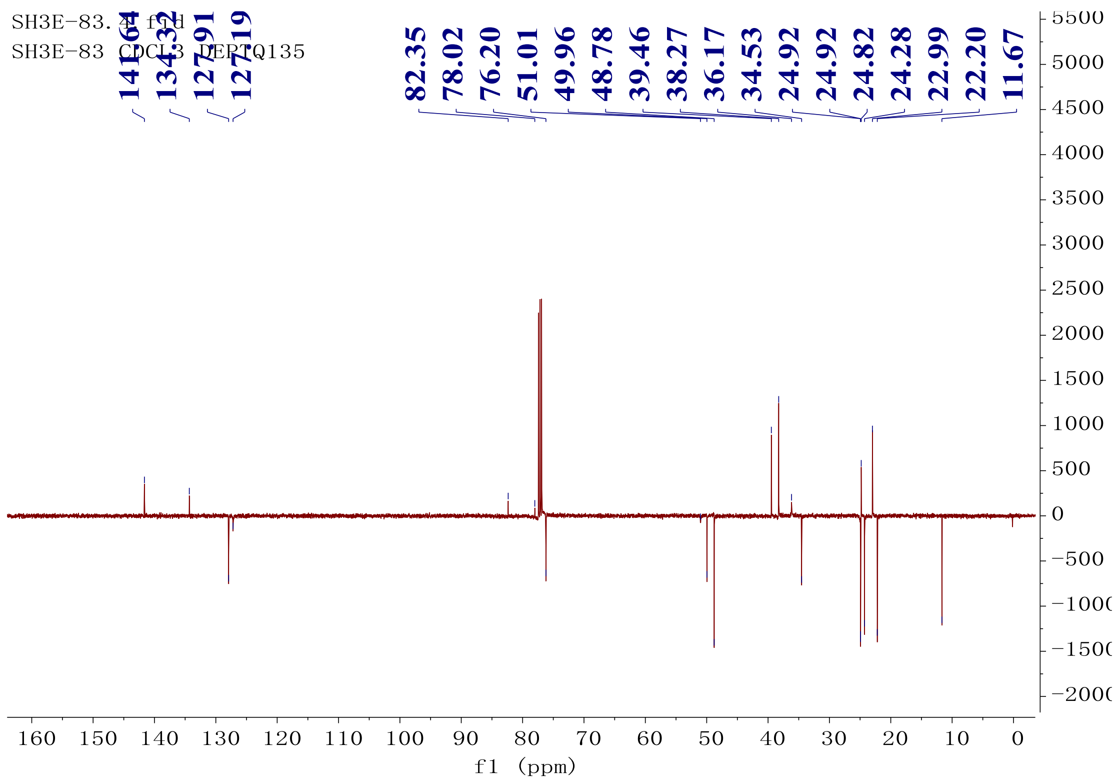

Figure S11. ^{13}C NMR spectrum (125 MHz) of compound **2** in $CDCl_3$

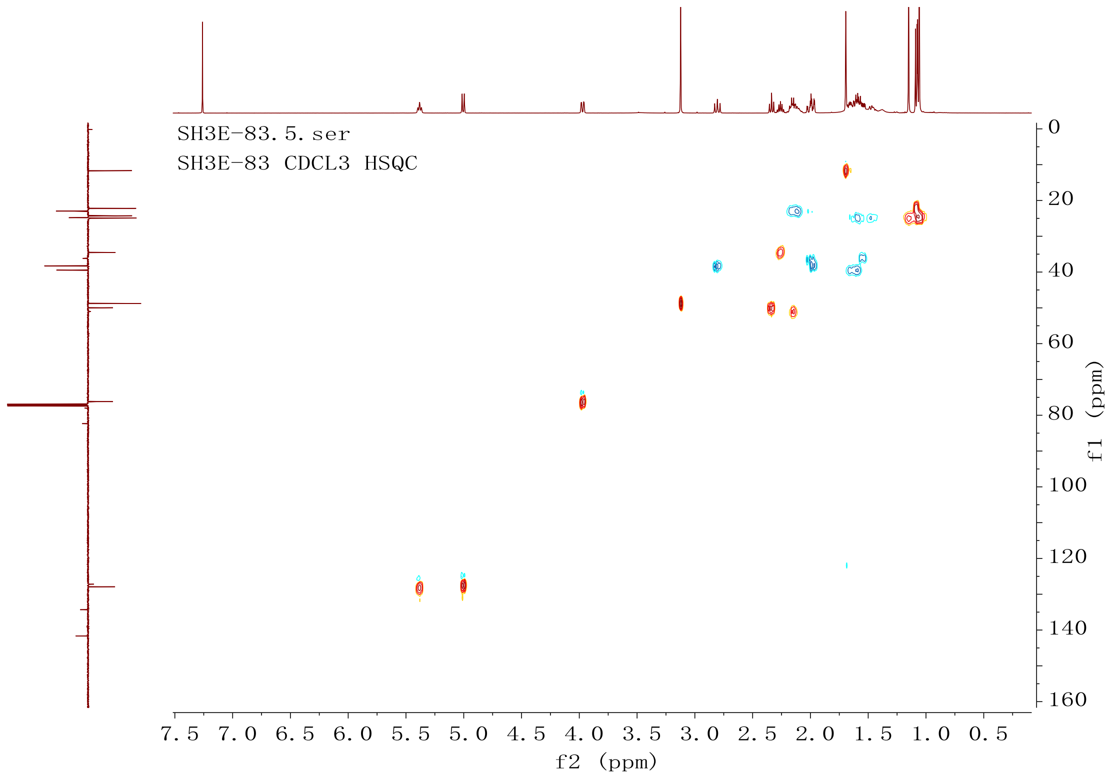

Figure S12. HSQC spectrum (500 MHz) of compound **2** in $CDCl_3$

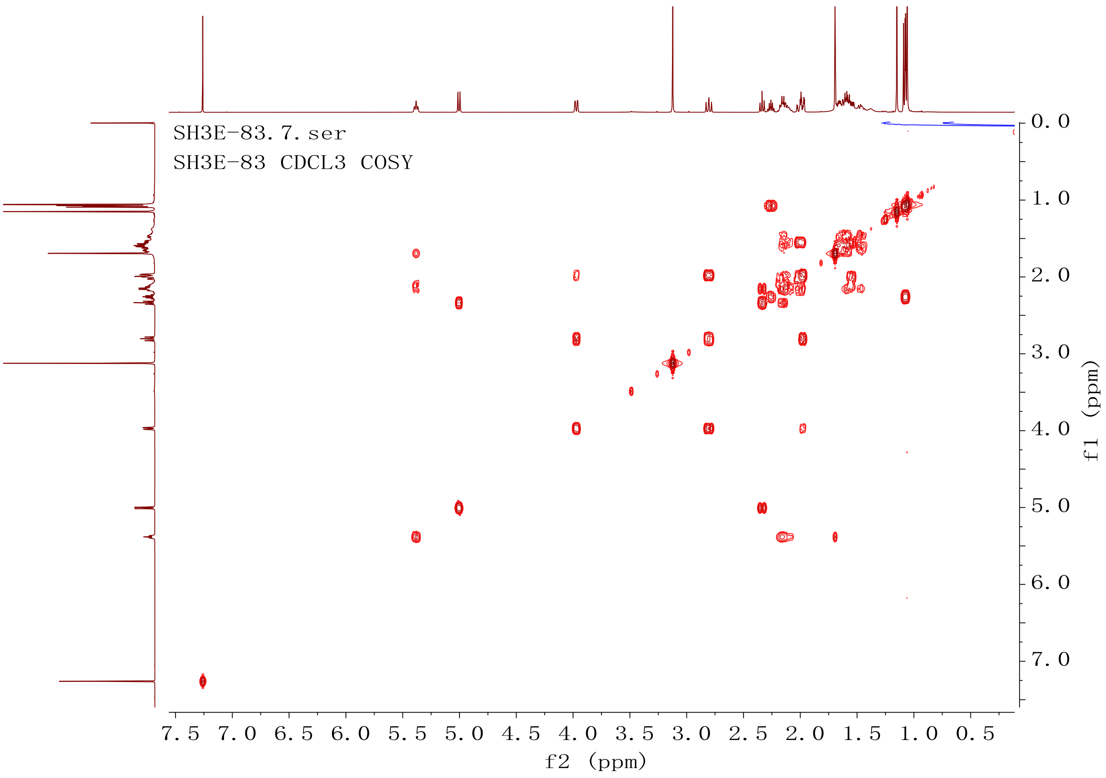

Figure S13. ^{1}H-^{1}H COSY spectrum (500 MHz) of compound **2** in $CDCl_3$

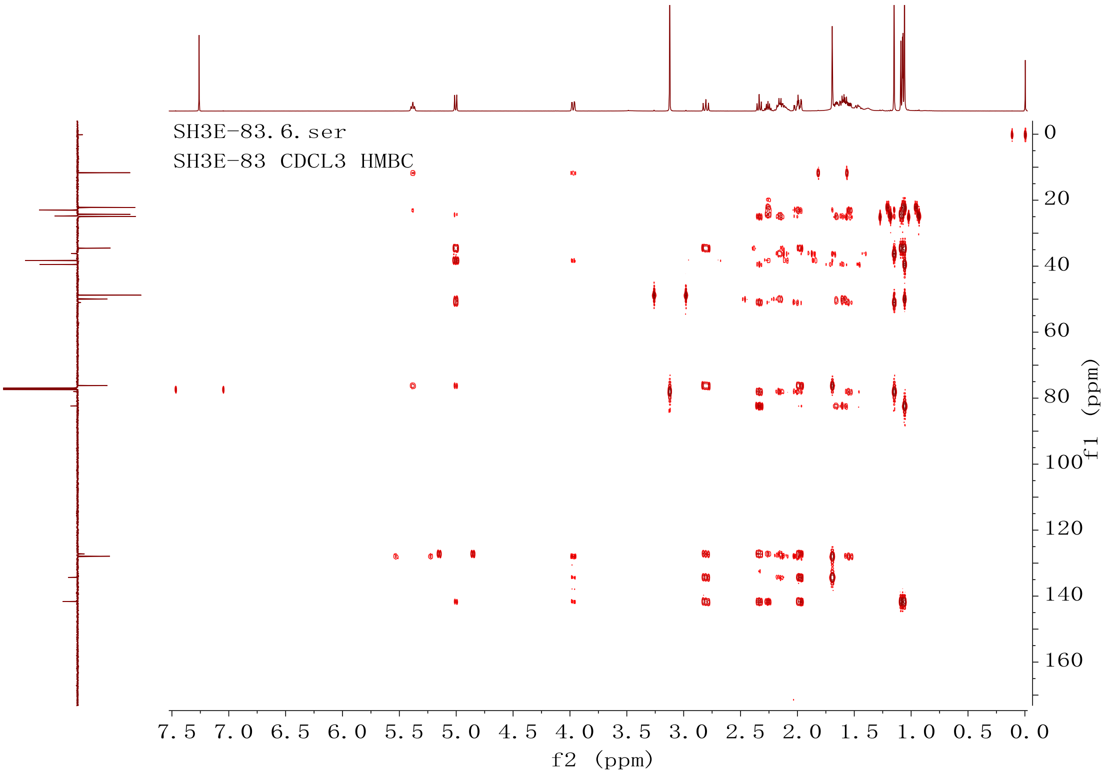

Figure S14. HMBC spectrum (500 MHz) of compound **2** in $CDCl_3$

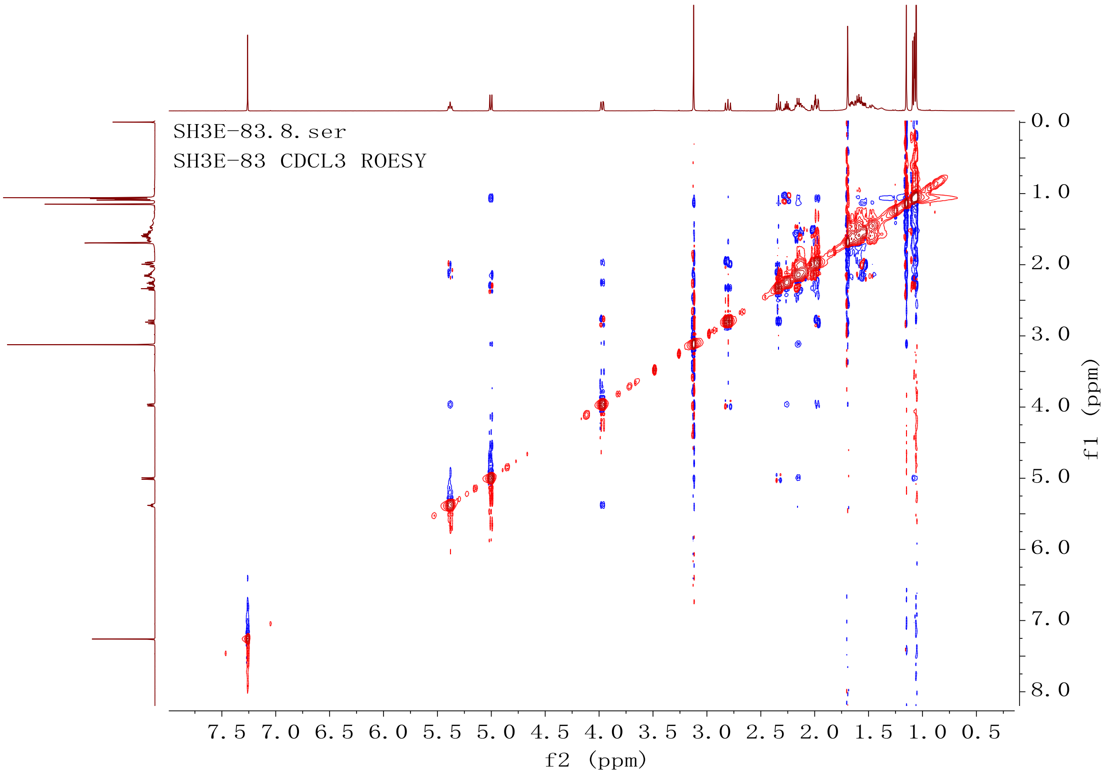

Figure S15. ROESY spectrum (500 MHz) of compound **2** in $CDCl_3$

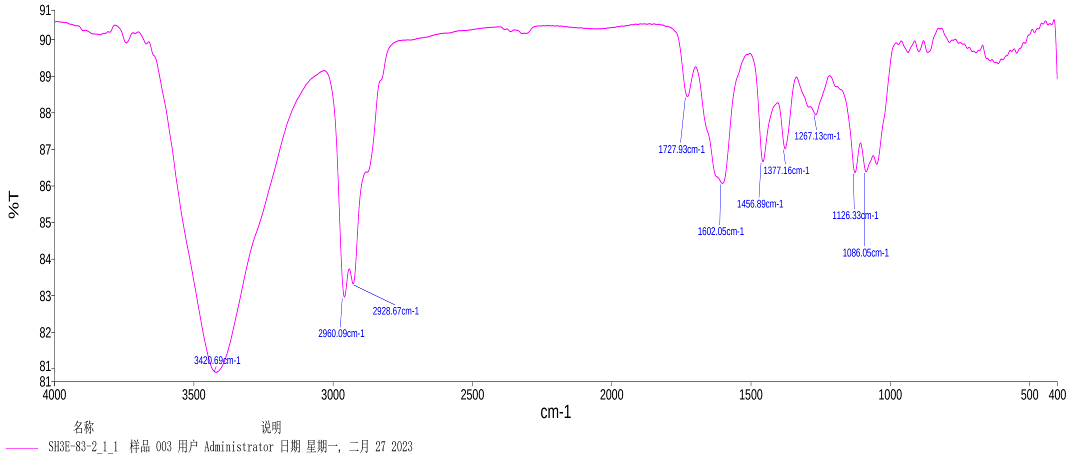

Figure S16. IR spectrum of compound **2**

Mass Spectrum SmartFormula Report

Analysis Info

Analysis Name	D:\2023\A501\WYL\20230106\B502-SH3E-83_BE1_01_6439.d	Acquisition Date	2023-01-06 17:12:43
Method	lc-ms_as_ms-0.5MIN-20221111.m	Operator	Demo User
Sample Name	B502-SH3E-83	Instrument	compact 8255754.20156
Comment			

Acquisition Parameter

Source Type	ESI	Ion Polarity	Positive	Set Nebulizer	1.8 Bar
Focus	Not active	Set Capillary	3500 V	Set Dry Heater	220 °C
Scan Begin	50 m/z	Set End Plate Offset	-500 V	Set Dry Gas	4.0 l/min
Scan End	2500 m/z	Set Charging Voltage	2000 V	Set Divert Valve	Waste
		Set Corona	0 nA	Set APCI Heater	0 °C

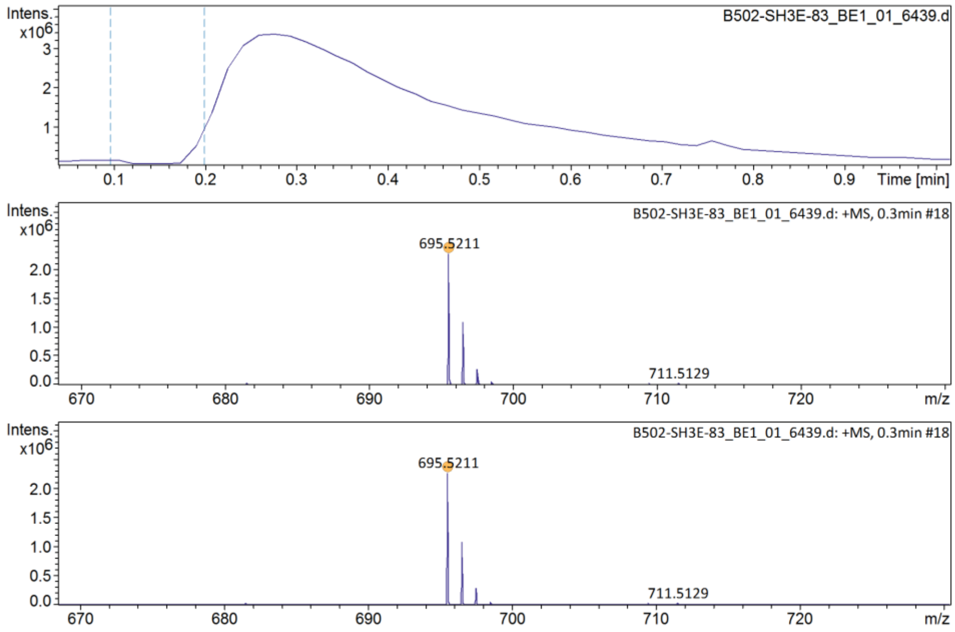

Meas. m/z	#	Ion Formula	m/z	err [ppm]	mSigma	# mSigma	Score	rdb	e⁻ Conf	N-Rule	Adduct
695.5211	1	C42H72NaO6	695.5221	1.5	6.1	1	100.00	7.0	even	ok	2M+Na

Figure S17. HR-ESIMS spectrum of compound **2**

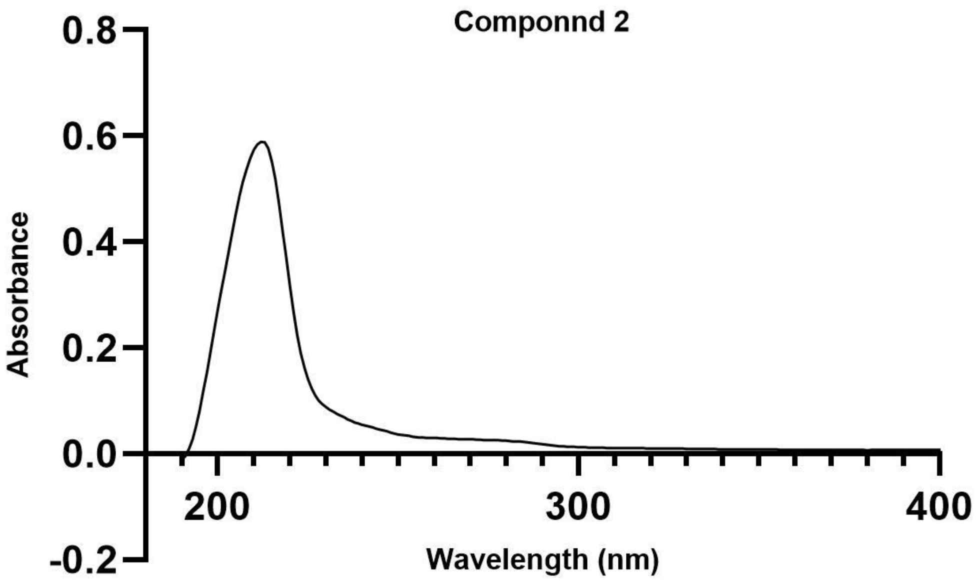

Figure S18. UV spectrum of compound **2**

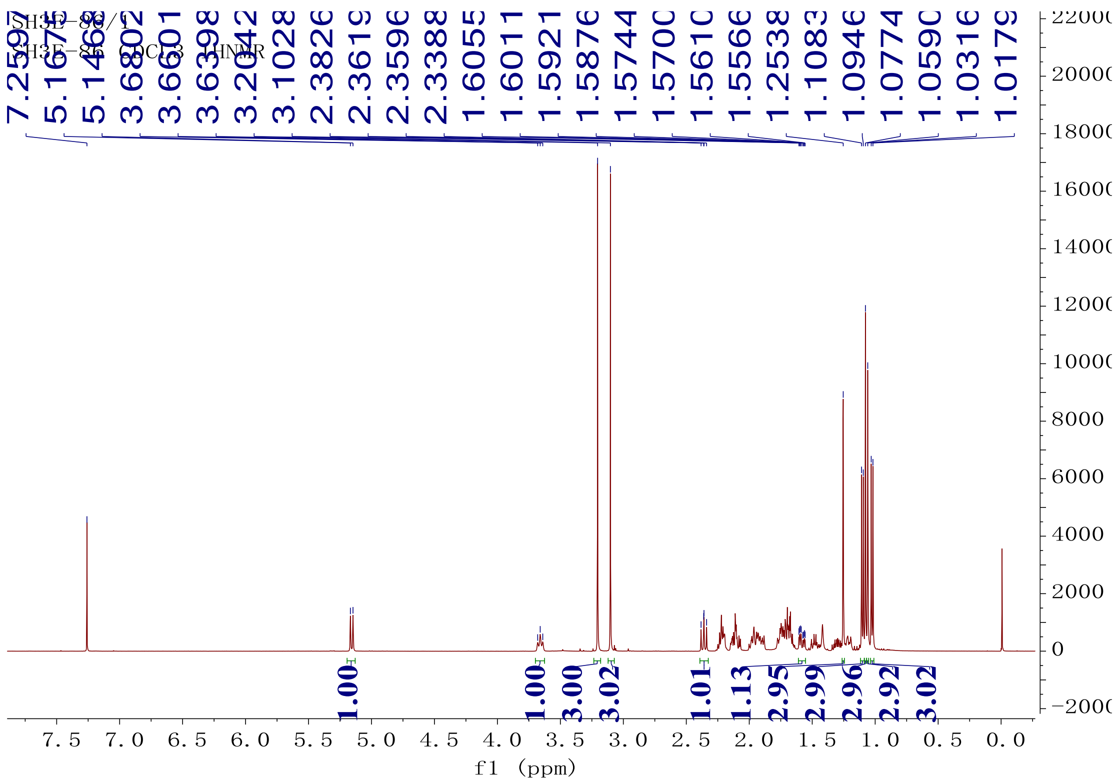

Figure S19. ^{1}H NMR spectrum (500 MHz) of compound **3** in $CDCl_3$

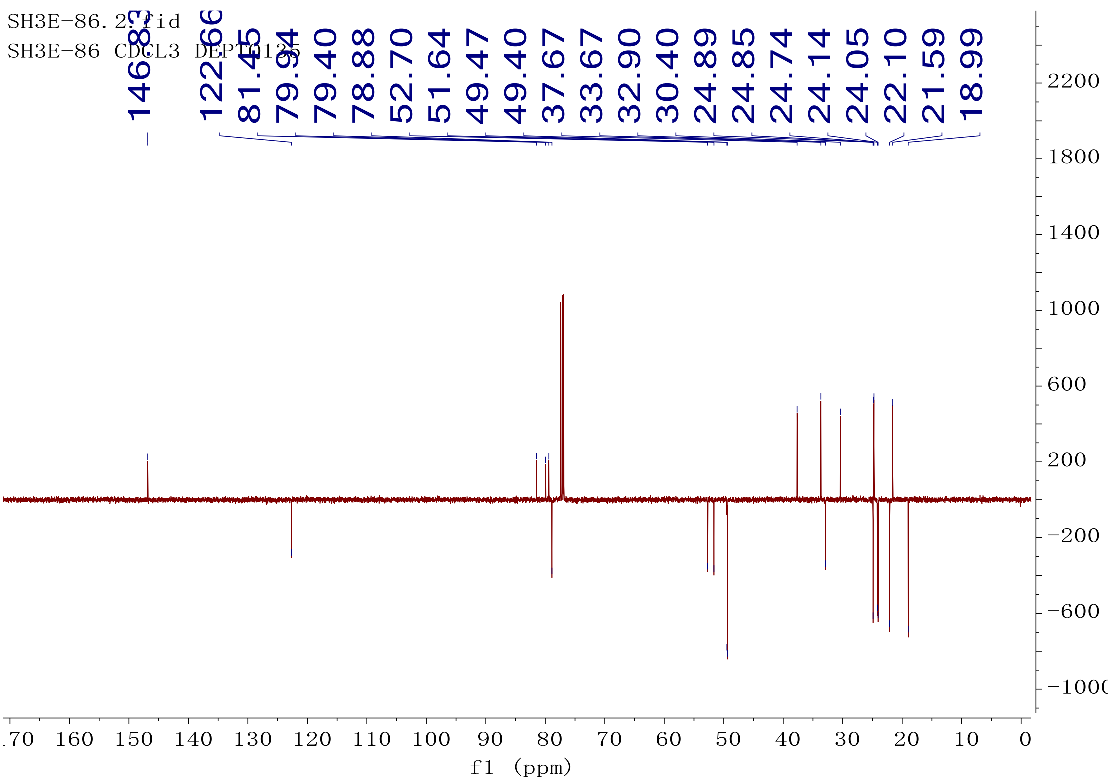

Figure S20. ^{13}C NMR spectrum (125 MHz) of compound **3** in $CDCl_3$

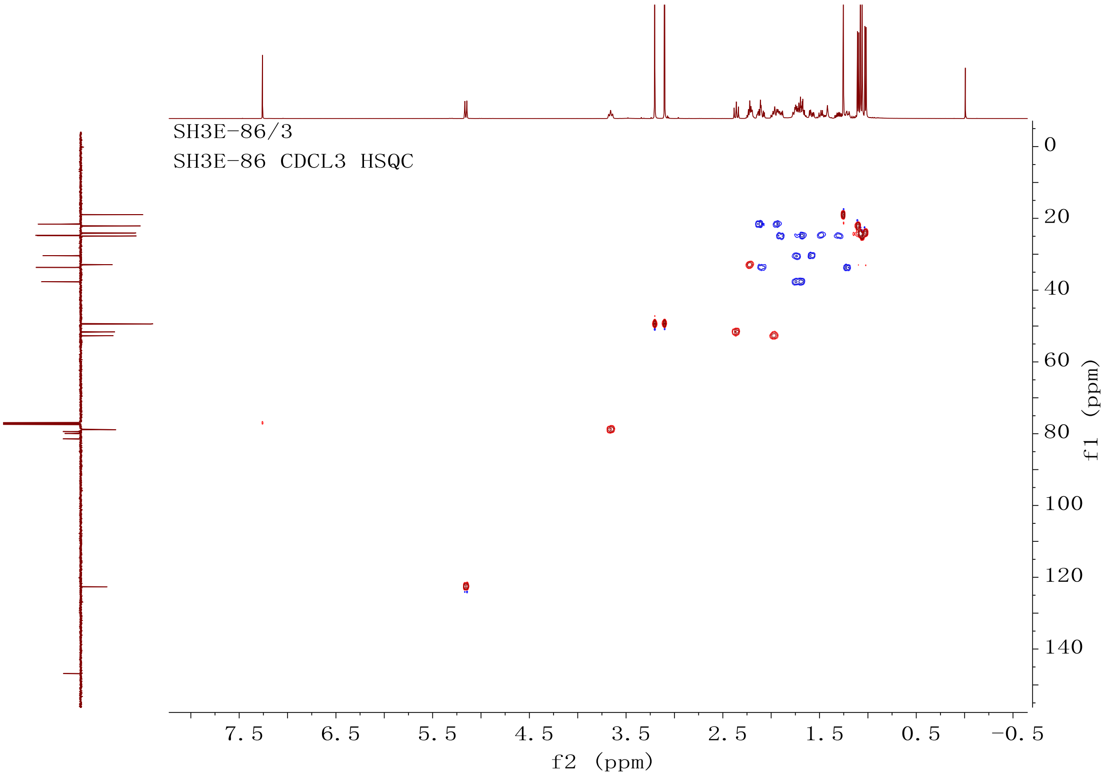

Figure S21. HSQC spectrum (500 MHz) of compound **3** in $CDCl_3$

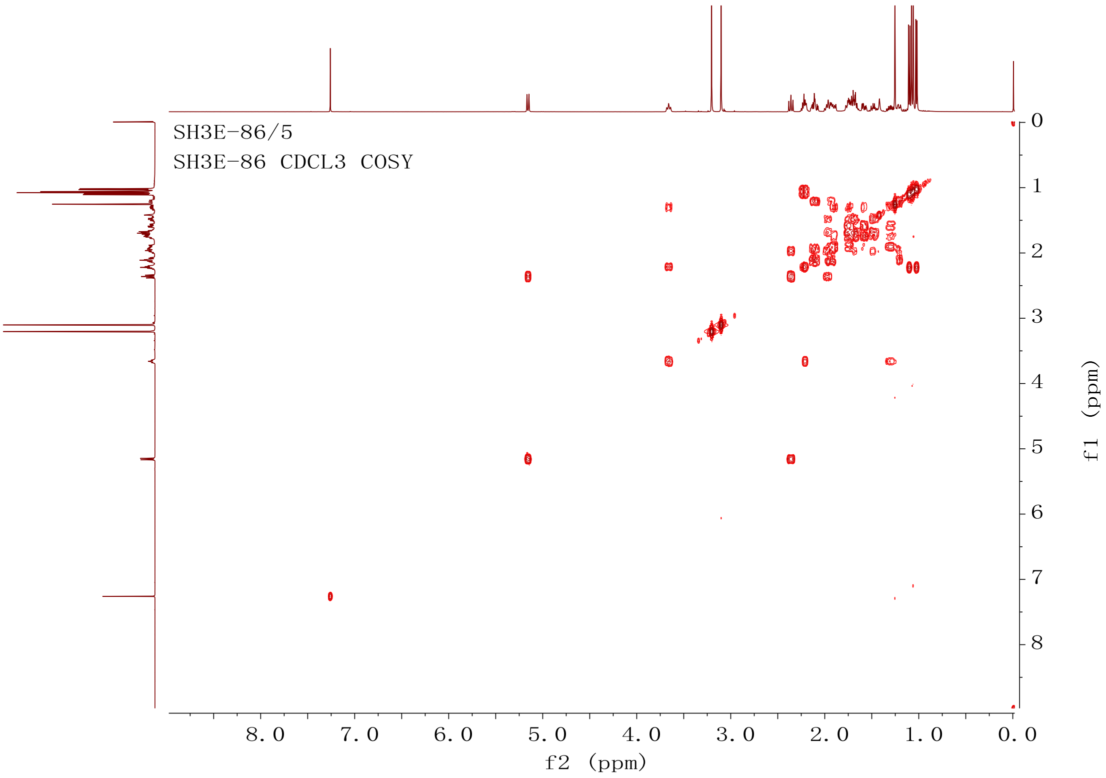

Figure S22. ^1H-^1H COSY spectrum (500 MHz) of compound **3** in $CDCl_3$

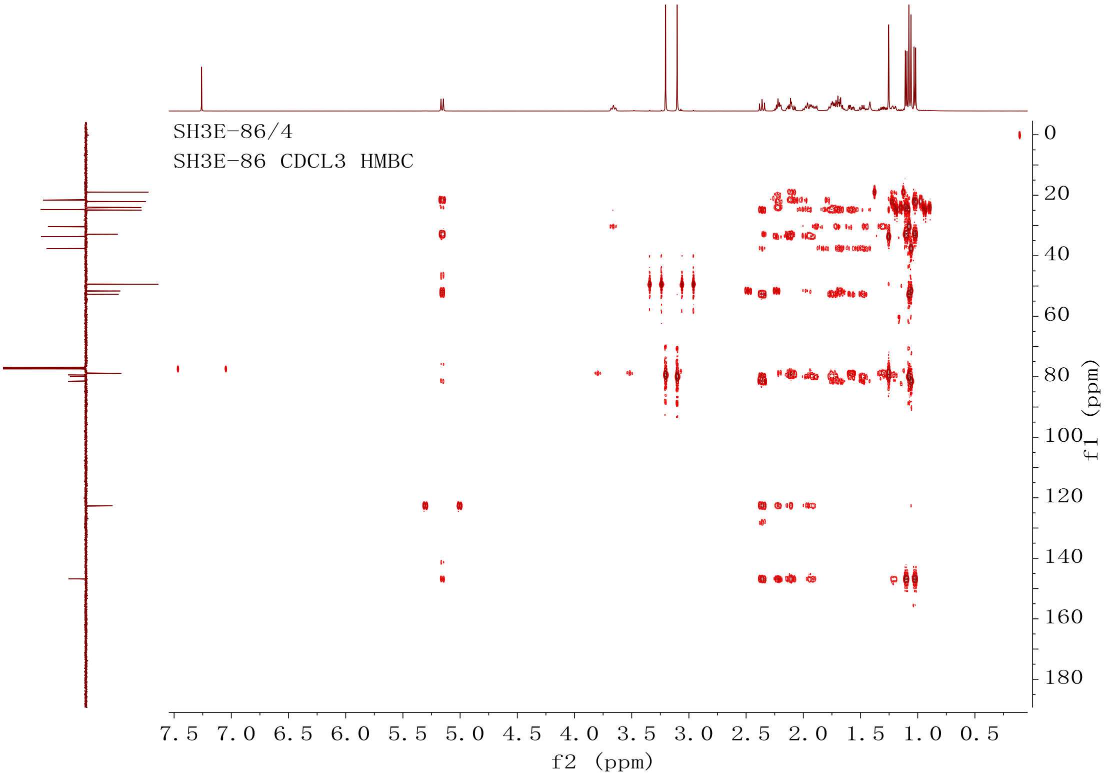

Figure S23. HMBC spectrum (500 MHz) of compound **3** in $CDCl_3$

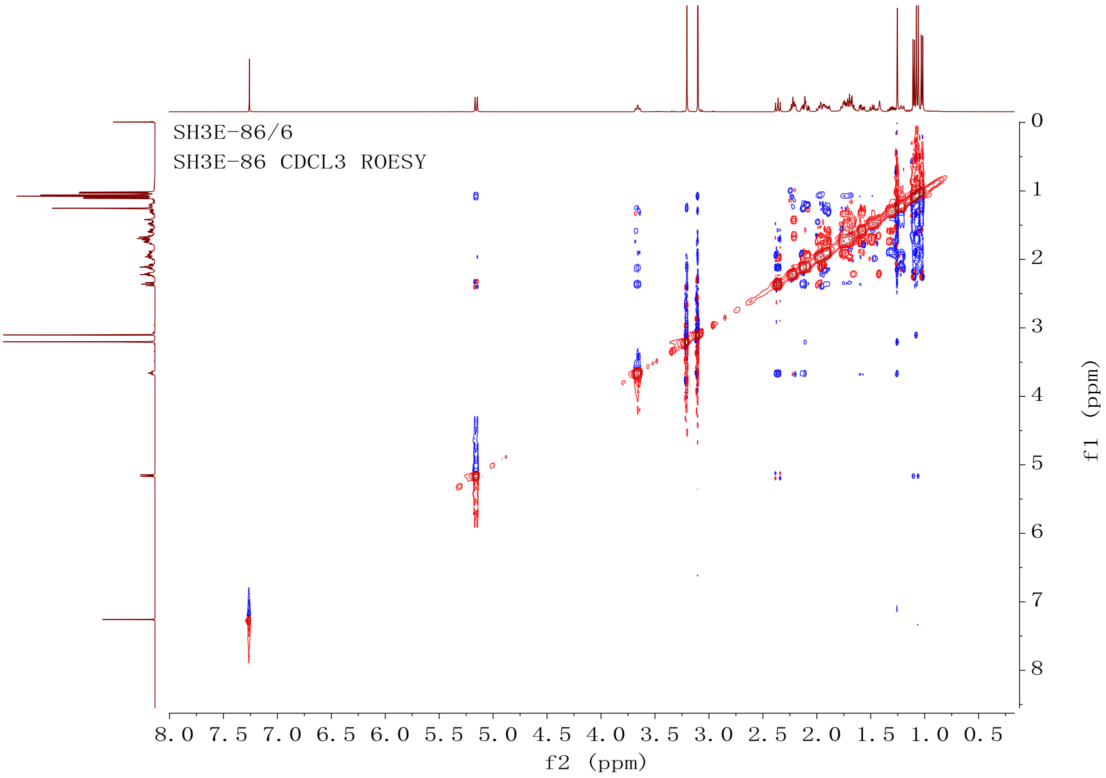

Figure S24. ROESY spectrum (500 MHz) of compound **3** in $CDCl_3$

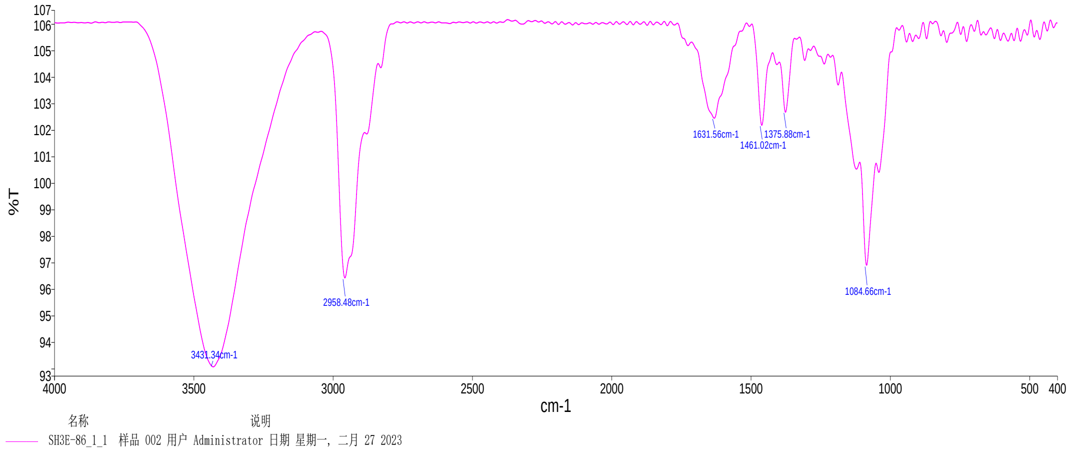

Figure S25. IR spectrum of compound **3**

Mass Spectrum SmartFormula Report

Analysis Info

Analysis Name	D:\2023\B502\20230517\SH3E-86_BA2_01_7483.d	Acquisition Date	2023-05-17 09:19:50
Method	lc-ms_as_ms-0.5MIN-20221111.m	Operator	Demo User
Sample Name	SH3E-86	Instrument	compact 8255754.20156
Comment			

Acquisition Parameter

Source Type	ESI	Ion Polarity	Positive	Set Nebulizer	1.8 Bar
Focus	Not active	Set Capillary	3500 V	Set Dry Heater	220 °C
Scan Begin	50 m/z	Set End Plate Offset	-500 V	Set Dry Gas	4.0 l/min
Scan End	2500 m/z	Set Charging Voltage	2000 V	Set Divert Valve	Waste
		Set Corona	0 nA	Set APCI Heater	0 °C

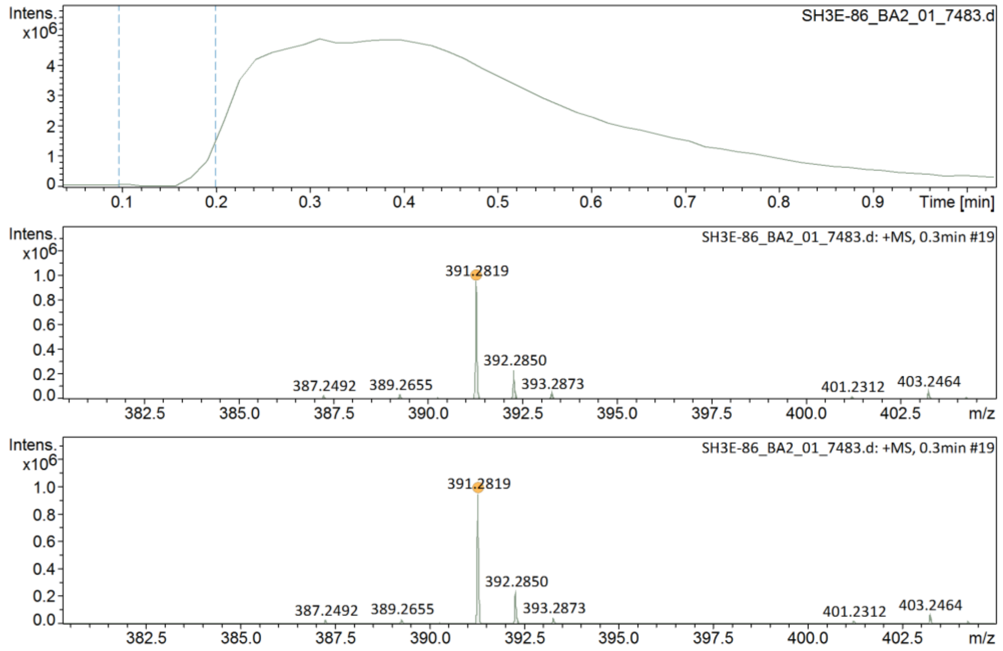

Meas. m/z	#	Ion Formula	m/z	err [ppm]	mSigma	# mSigma	Score	rdb	e⁻ Conf	N-Rule	Adduct
391.2819	1	C22H40NaO4	391.2819	-0.2	3.1	1	100.00	3.0	even	ok	M+Na

Figure S26. HR-ESIMS spectrum of compound **3**

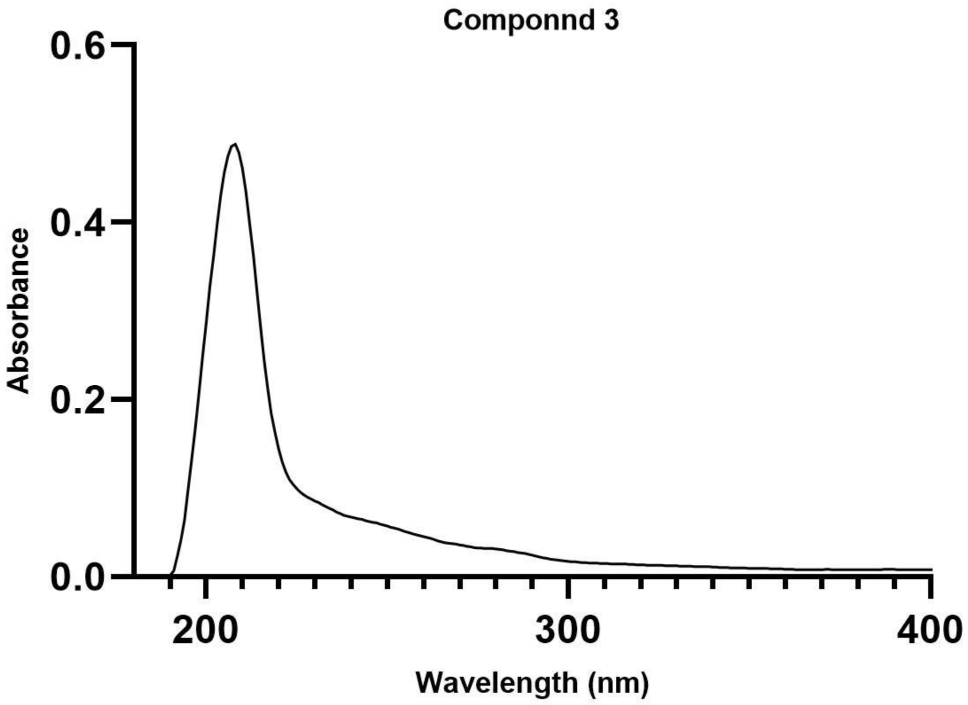

Figure S27. UV spectrum of compound **3**

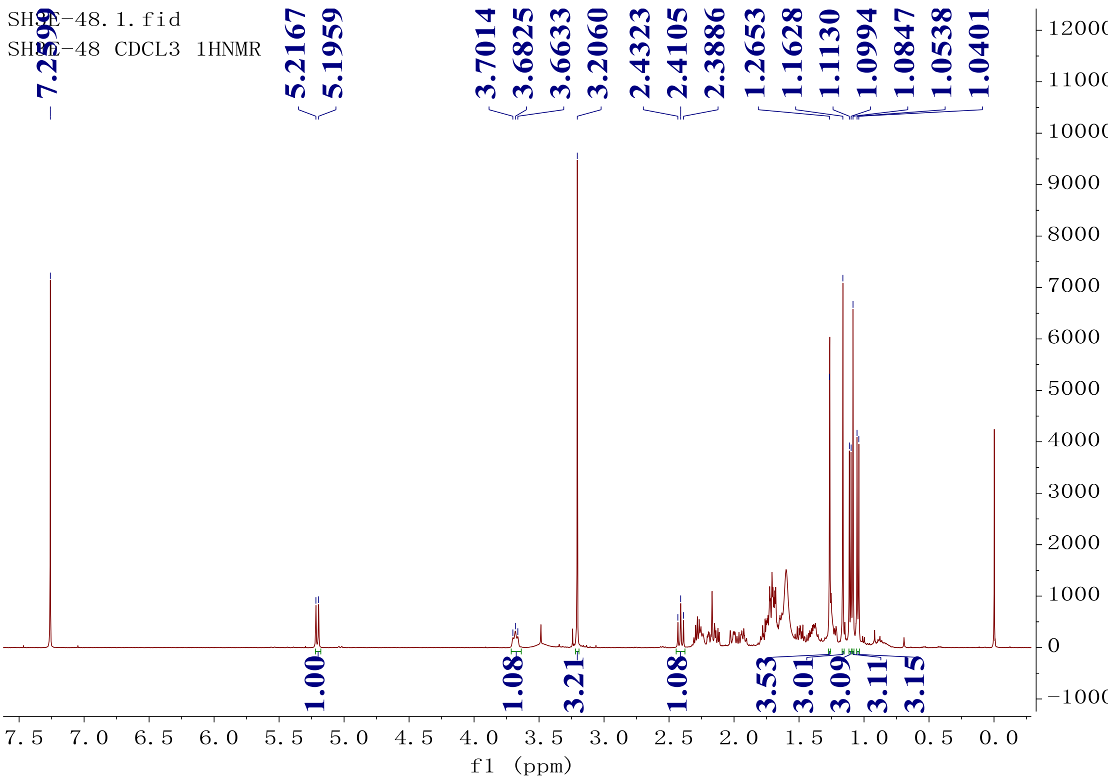

Figure S28. ^{1}H NMR spectrum (500 MHz) of compound **4** in $CDCl_3$

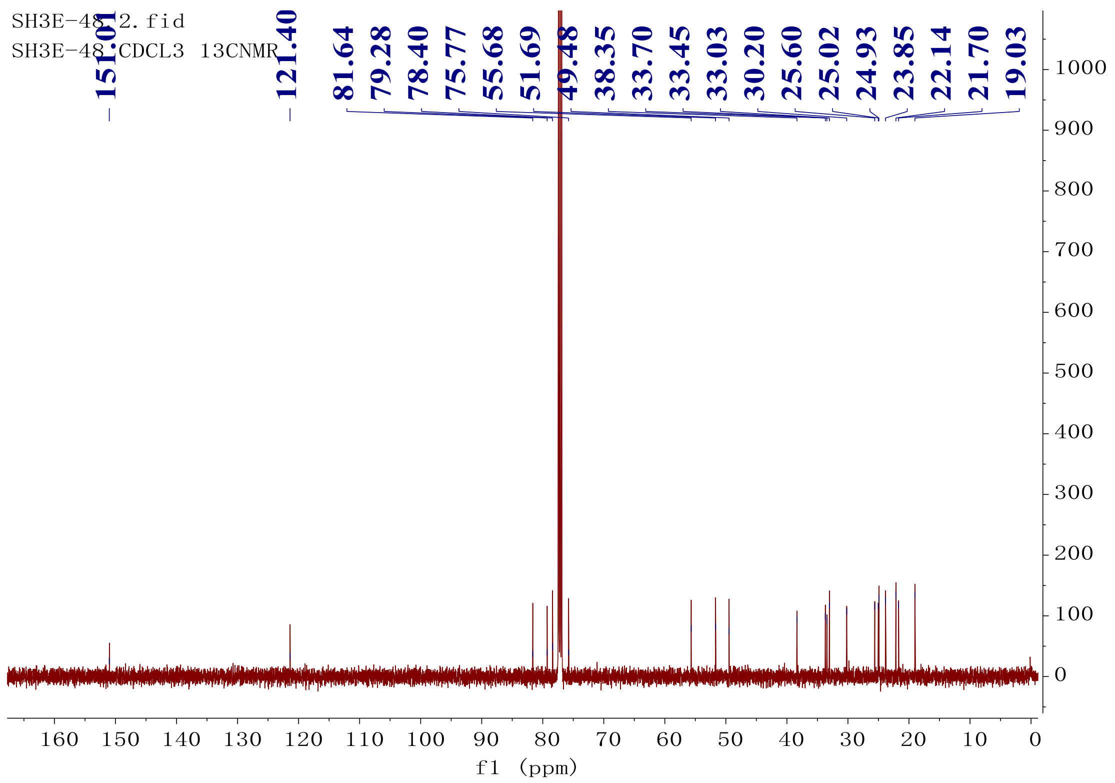

Figure S29. ^{13}C NMR spectrum (125 MHz) of compound **4** in $CDCl_3$

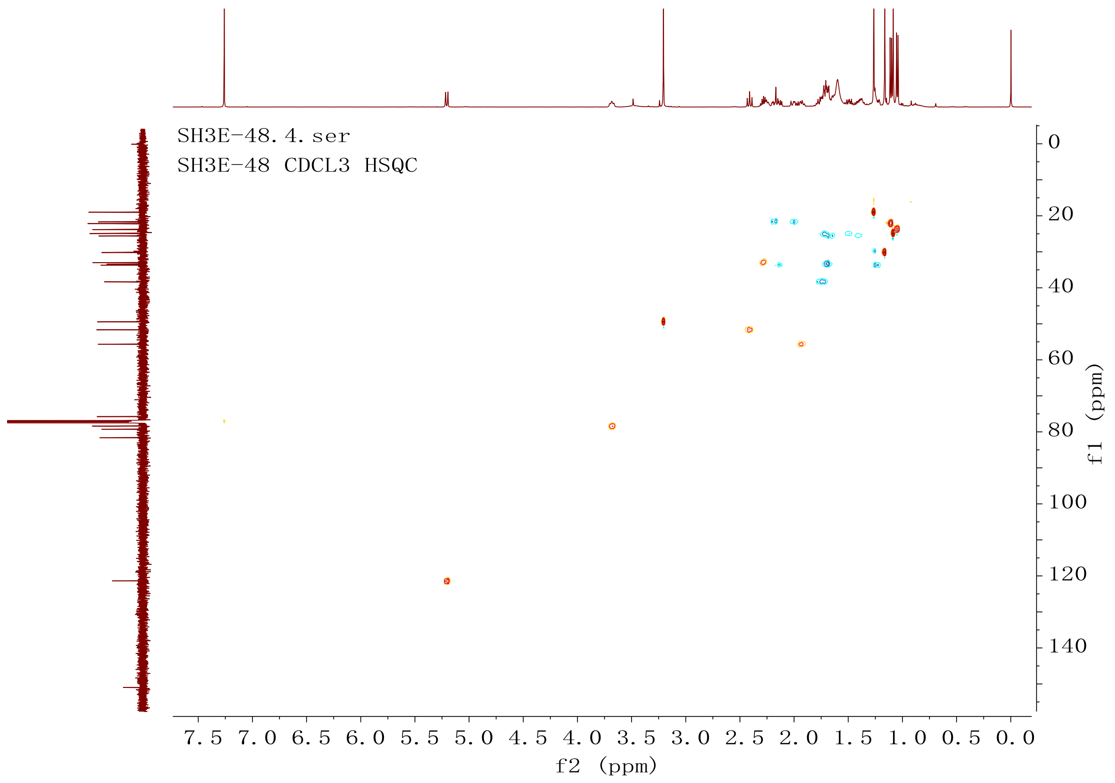

Figure S30. HSQC spectrum (500 MHz) of compound **4** in $CDCl_3$

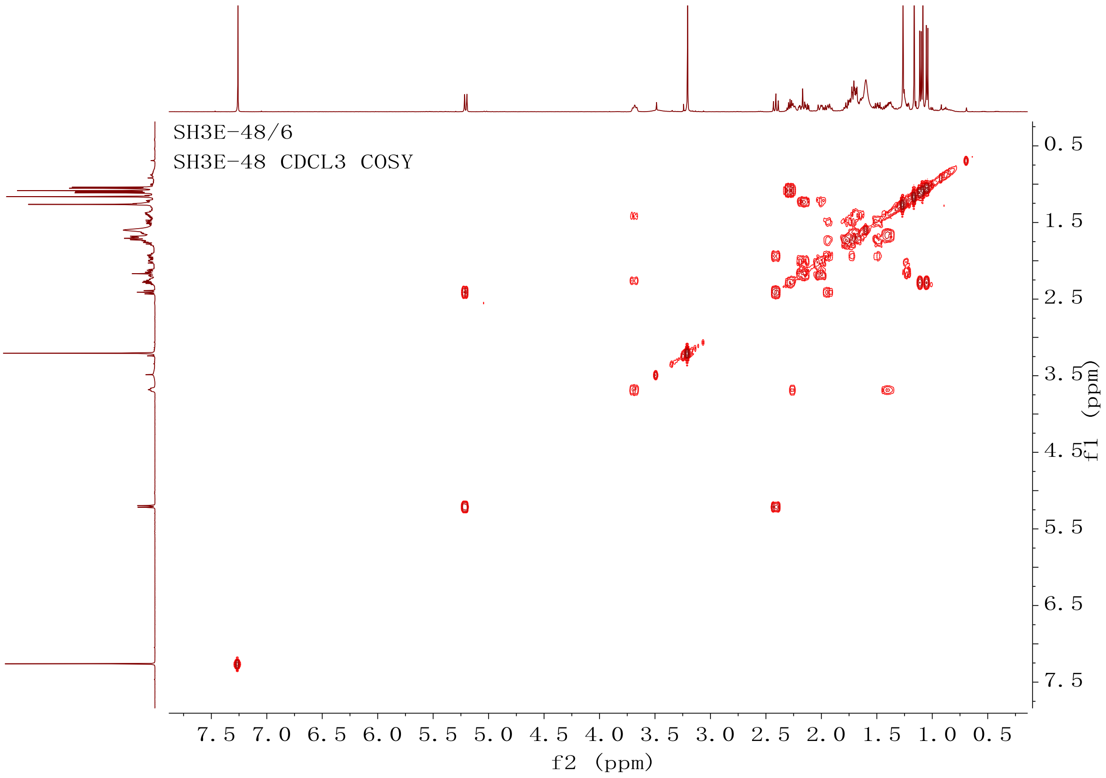

Figure S31. ^{1}H-^{1}H COSY spectrum (500 MHz) of compound **4** in $CDCl_3$

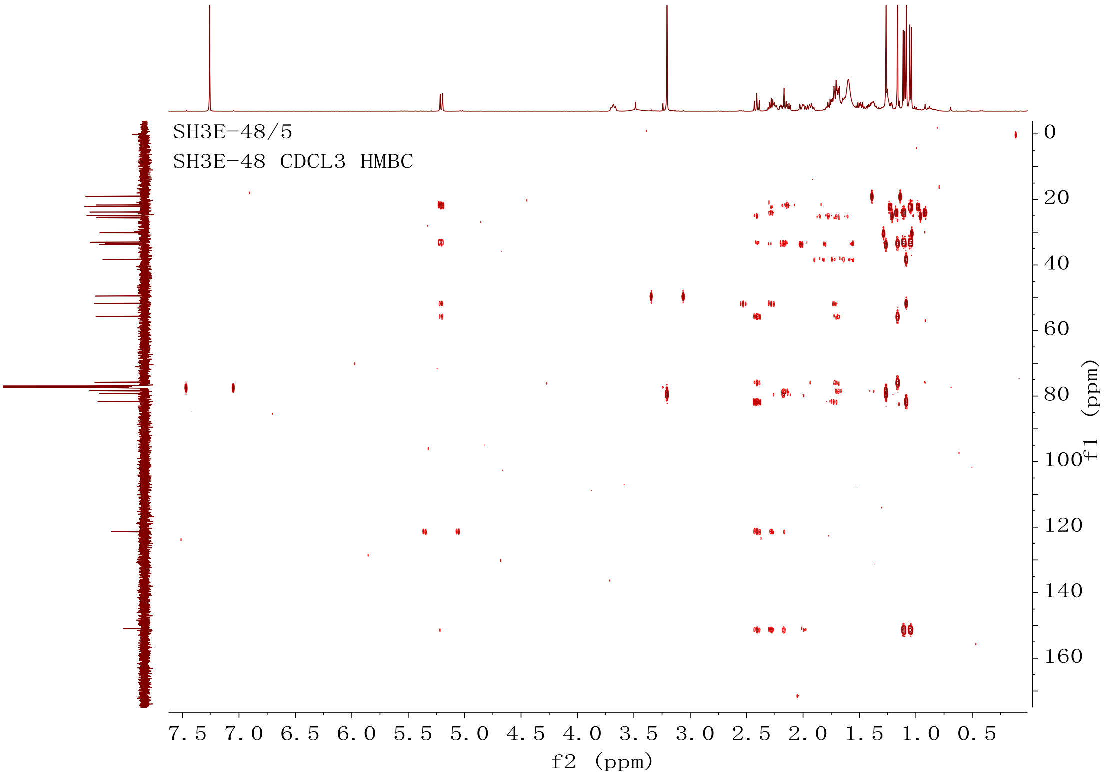

Figure S32. HMBC spectrum (500 MHz) of compound **4** in $CDCl_3$

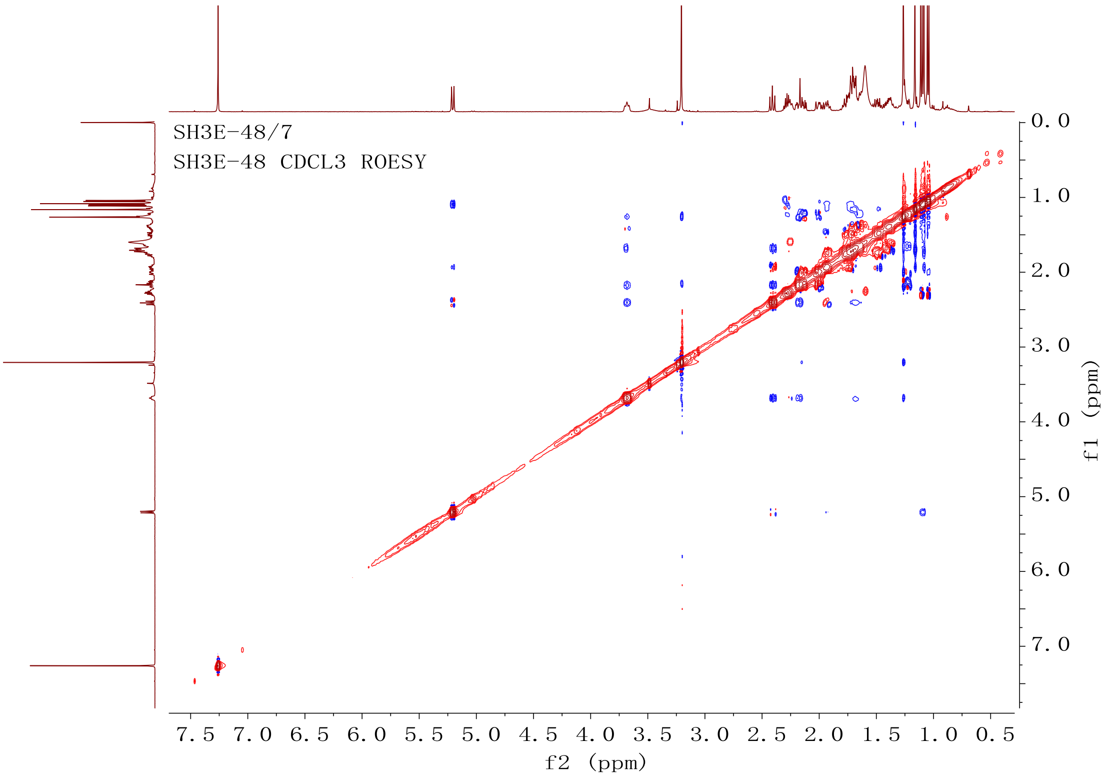

Figure S33. ROESY spectrum (500 MHz) of compound **4** in $CDCl_3$

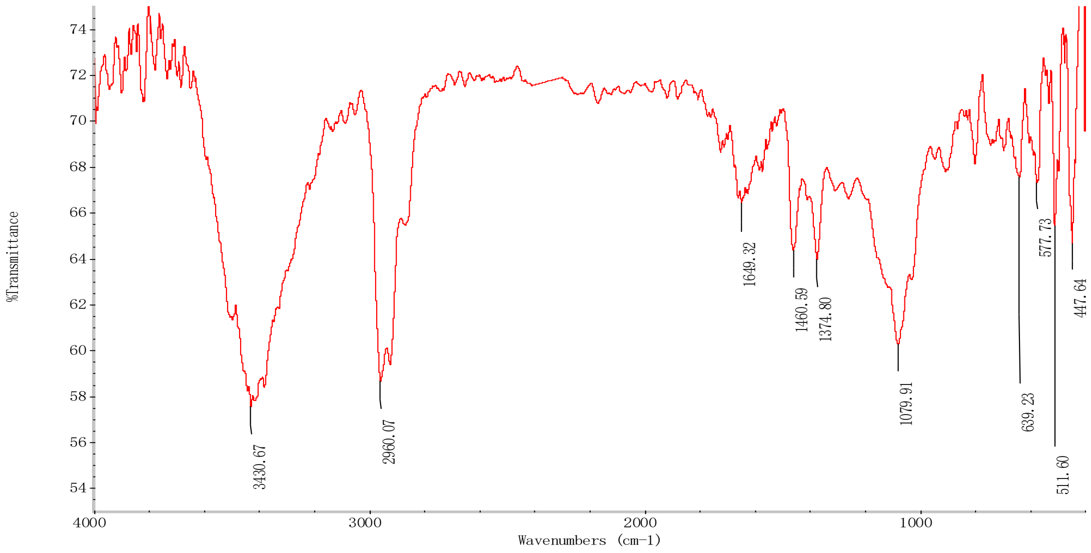

Figure S34. IR spectrum of compound **4**

Mass Spectrum SmartFormula Report

Analysis Info

Analysis Name	D:\2023\B502\20230919\SH3E-48_BB3_01_8667.d	Acquisition Date	2023-09-19 09:26:14
Method	lc-ms_as_ms-1min-20230519.m	Operator	Demo User
Sample Name	SH3E-48	Instrument	compact 8255754.20156
Comment			

Acquisition Parameter

Source Type	ESI	Ion Polarity	Positive	Set Nebulizer	1.8 Bar
Focus	Not active	Set Capillary	3500 V	Set Dry Heater	220 °C
Scan Begin	50 m/z	Set End Plate Offset	-500 V	Set Dry Gas	4.0 l/min
Scan End	2500 m/z	Set Charging Voltage	2000 V	Set Divert Valve	Waste
		Set Corona	0 nA	Set APCI Heater	0 °C

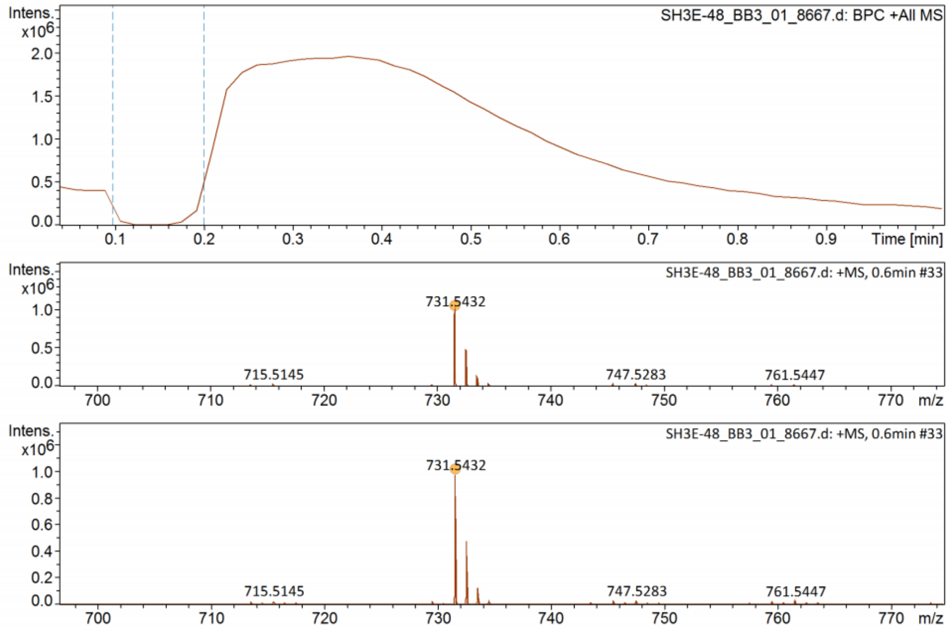

Meas. m/z	#	Ion Formula	m/z	err [ppm]	mSigma	# mSigma	Score	rdb	e⁻ Conf	N-Rule	Adduct
731.5432	1	C42H76NaO8	731.5432	0.0	10.4	2	100.00	5.0	even	ok	2M+Na

Figure S35. HR-ESIMS spectrum of compound **4**

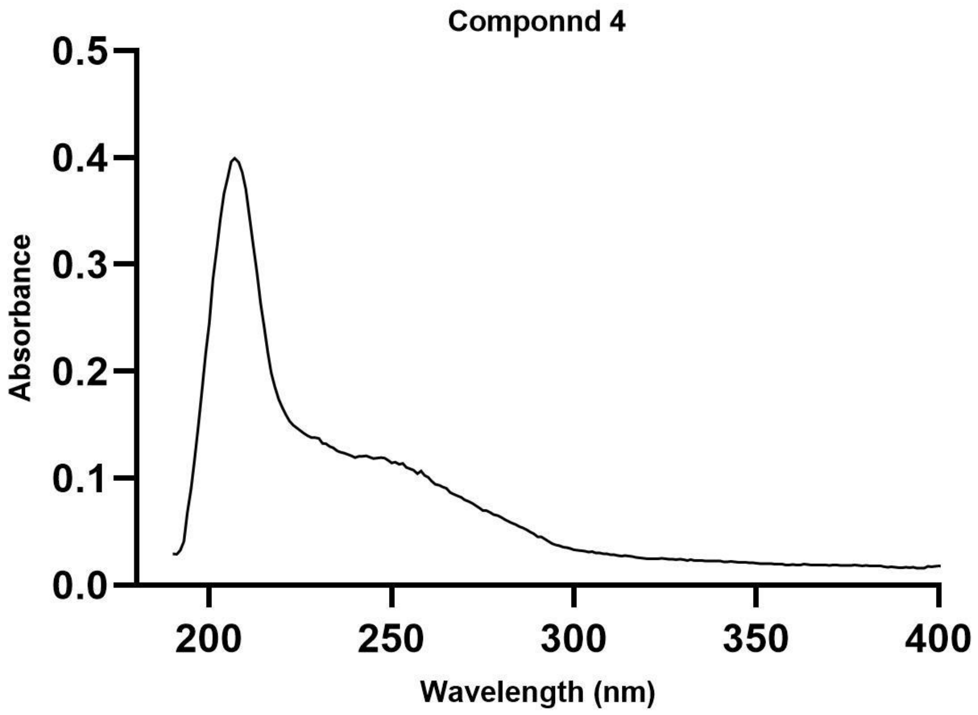

Figure S36. UV spectrum of compound **4**

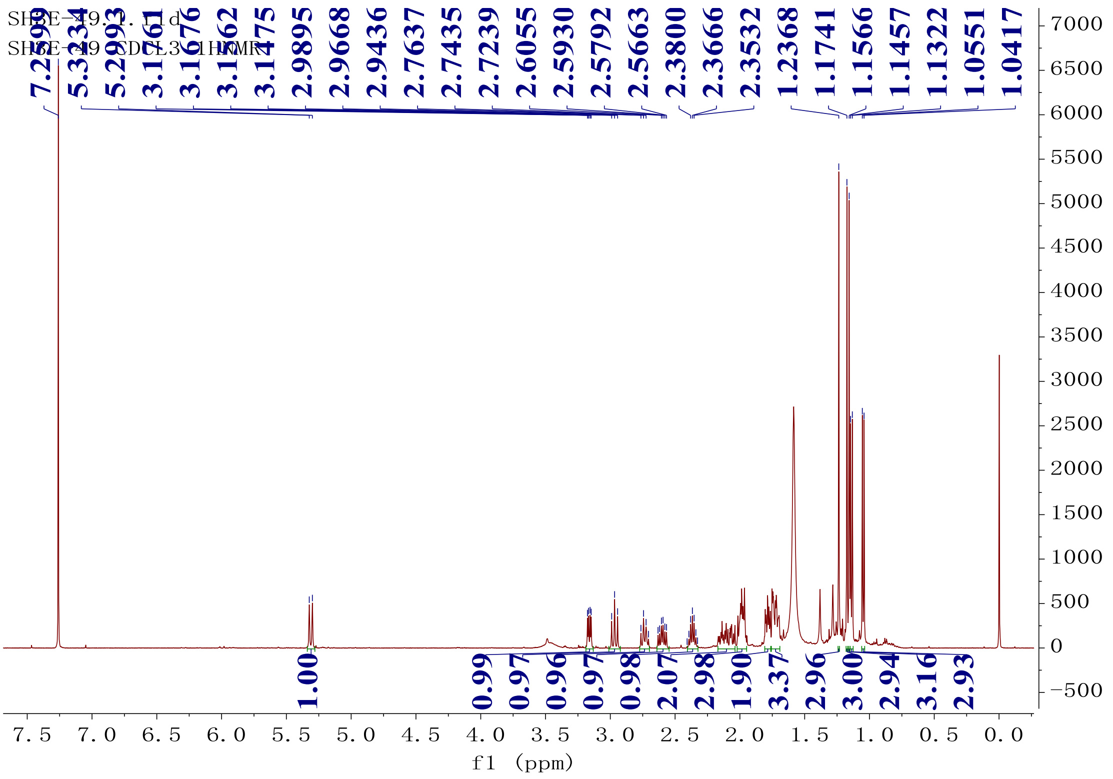

Figure S37. ^{1}H NMR spectrum (500 MHz) of compound **5** in $CDCl_3$

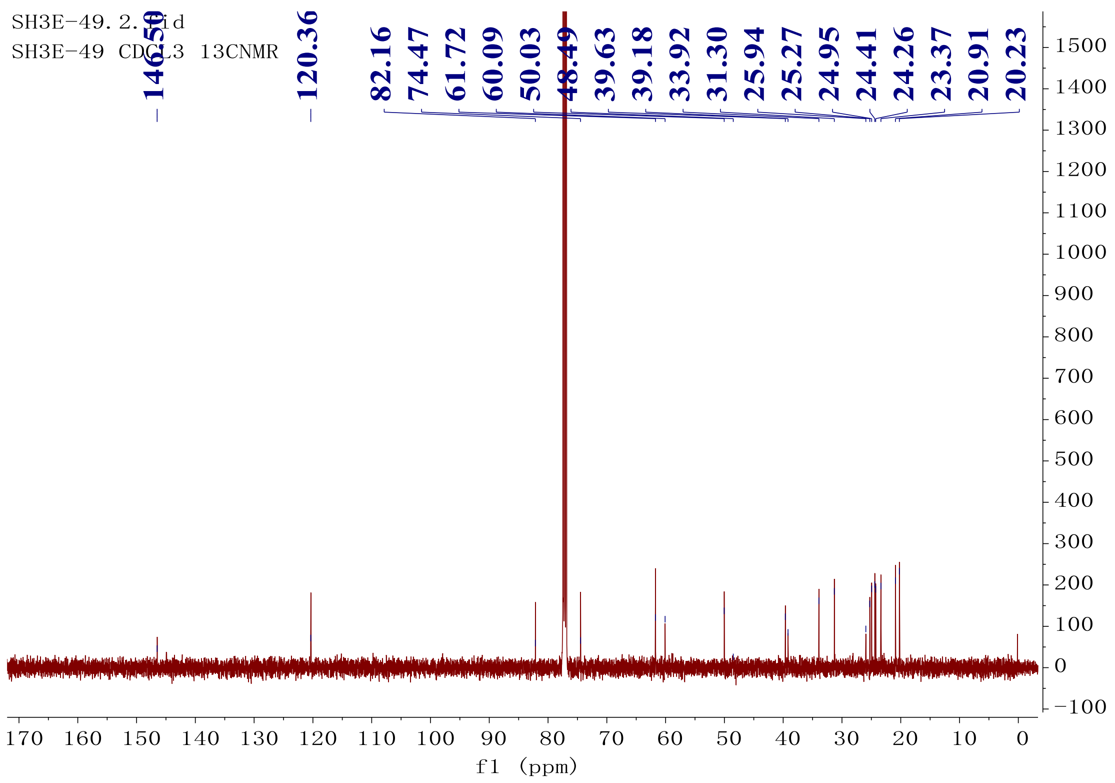

Figure S38. ^{13}C NMR spectrum (125 MHz) of compound **5** in $CDCl_3$

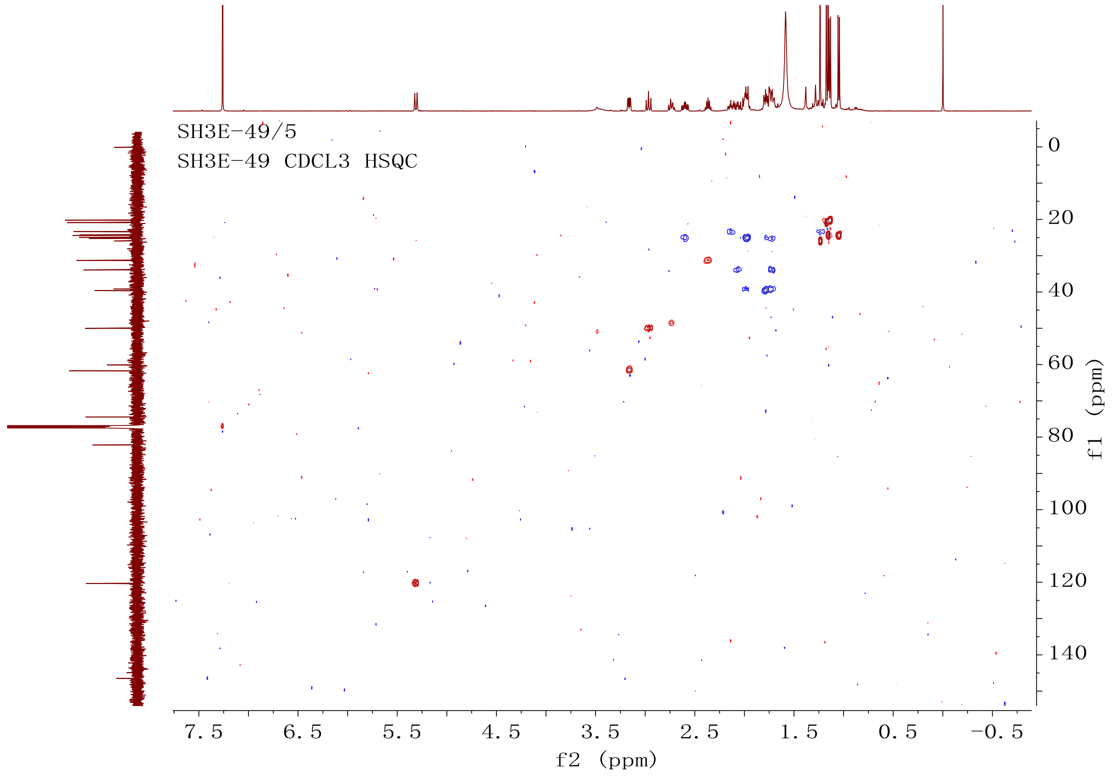

Figure S39. HSQC spectrum (500 MHz) of compound **5** in $CDCl_3$

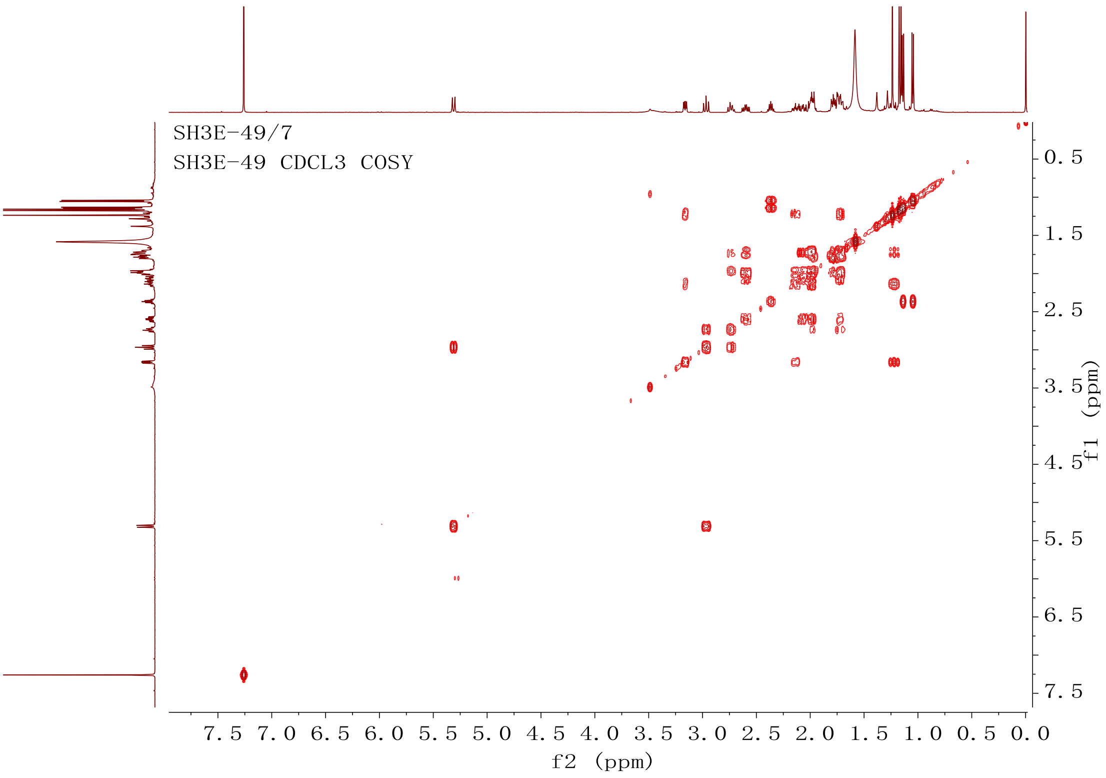

Figure S40. ^1H-^1H COSY spectrum (500 MHz) of compound **5** in $CDCl_3$

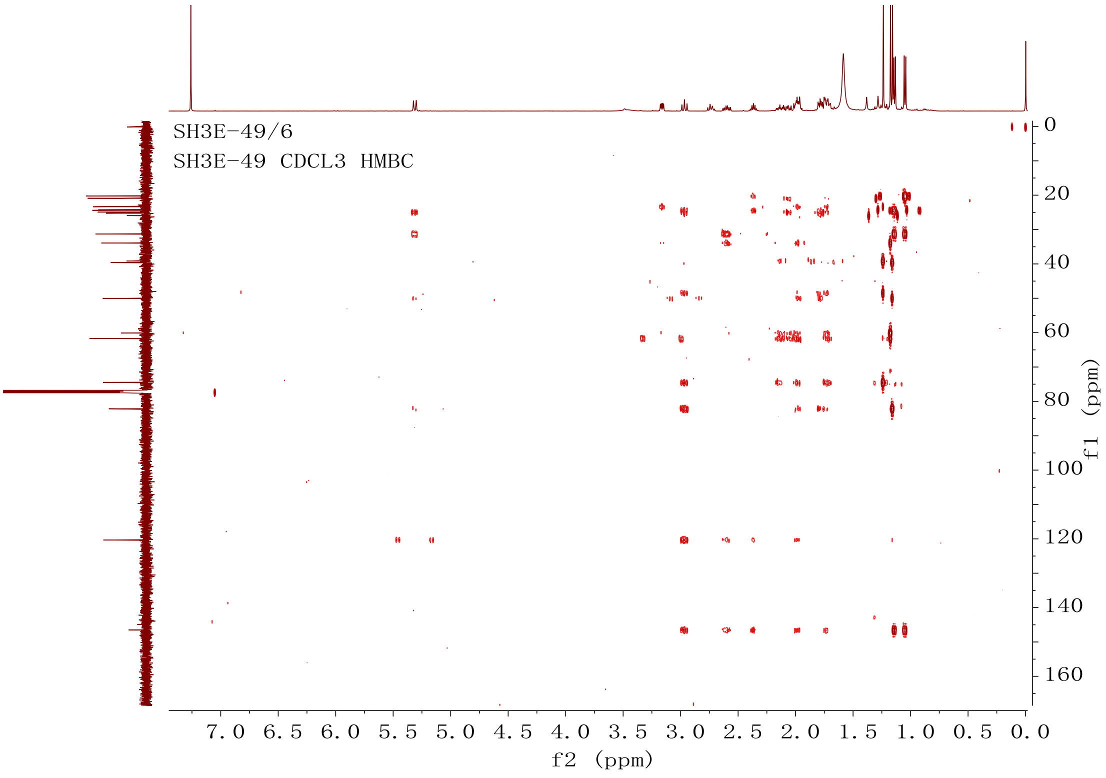

Figure S41. HMBC spectrum (500 MHz) of compound **5** in $CDCl_3$

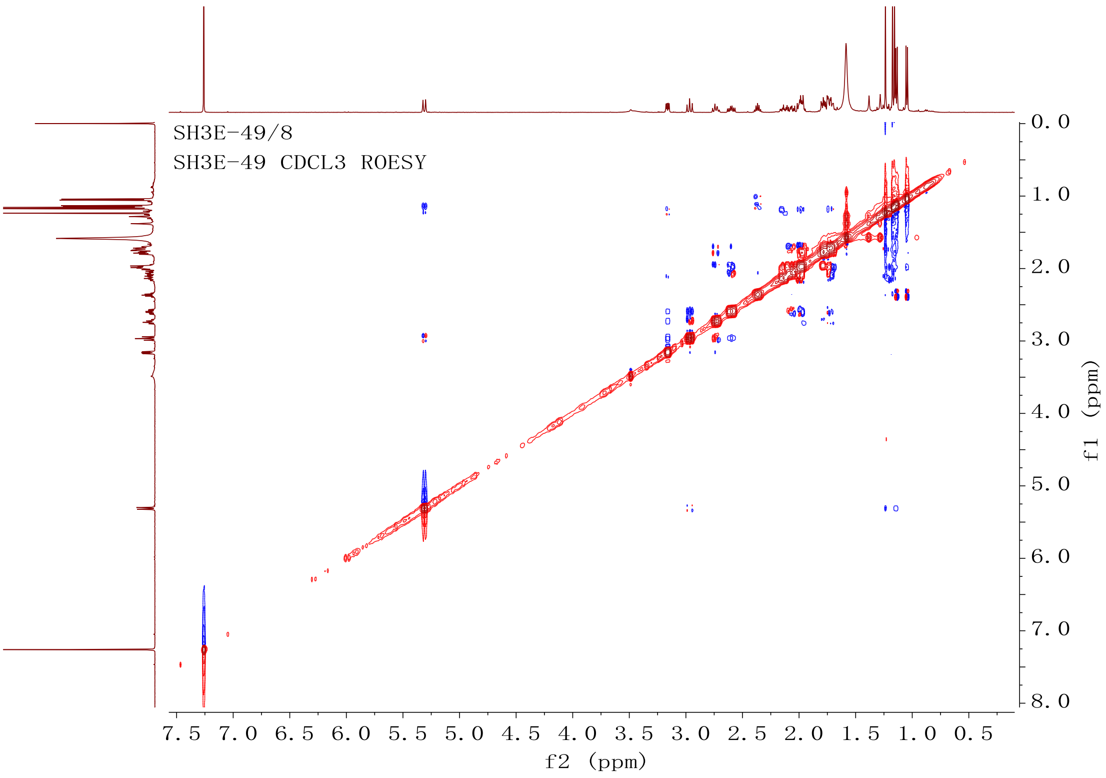

Figure S42. ROESY spectrum (500 MHz) of compound **5** in $CDCl_3$

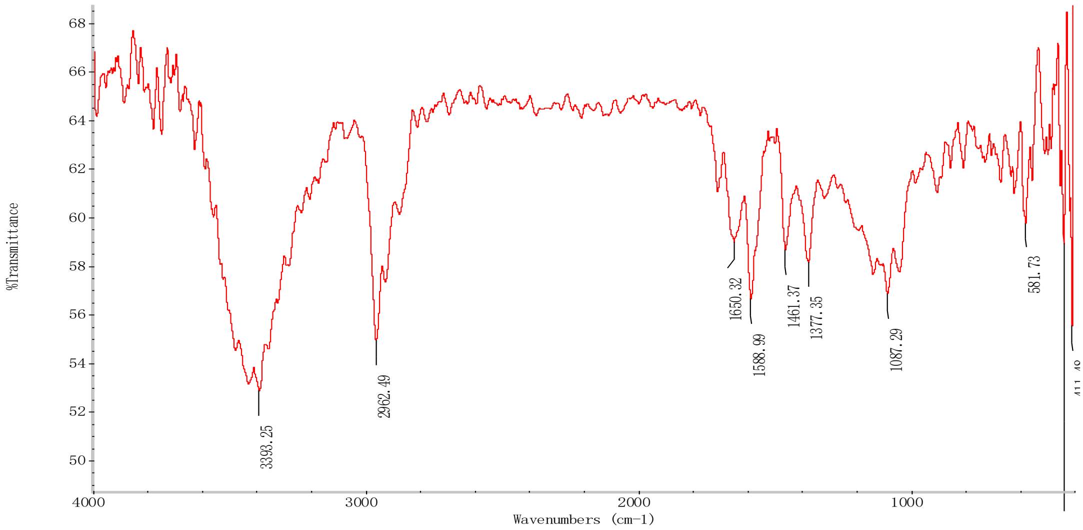

Figure S43. IR spectrum of compound **5**

Mass Spectrum SmartFormula Report

Analysis Info

Analysis Name	D:\Data\A501\WYL\2022\20221122\B502-SH3E-49_GA1_01_5680.d	Acquisition Date	2022-11-22 16:38:41
Method	lc-ms_as_ms-0.5MIN-20221111.m	Operator	Demo User
Sample Name	B502-SH3E-49	Instrument	compact 8255754.20156
Comment			

Acquisition Parameter

Source Type	ESI	Ion Polarity	Positive	Set Nebulizer	1.8 Bar
Focus	Not active	Set Capillary	3500 V	Set Dry Heater	220 °C
Scan Begin	50 m/z	Set End Plate Offset	-500 V	Set Dry Gas	4.0 l/min
Scan End	2500 m/z	Set Charging Voltage	2000 V	Set Divert Valve	Waste
		Set Corona	0 nA	Set APCI Heater	0 °C

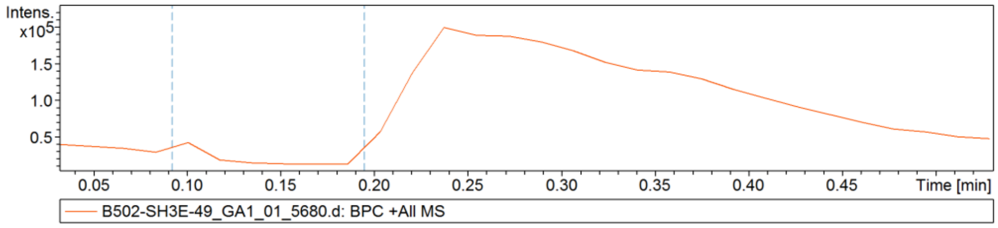

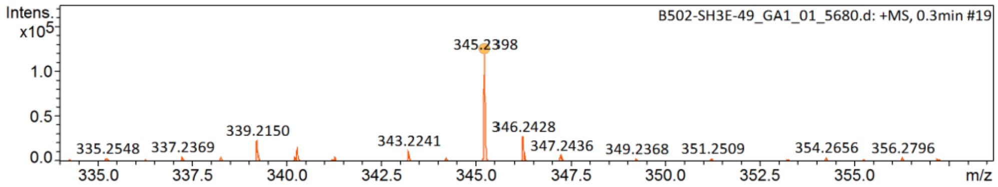

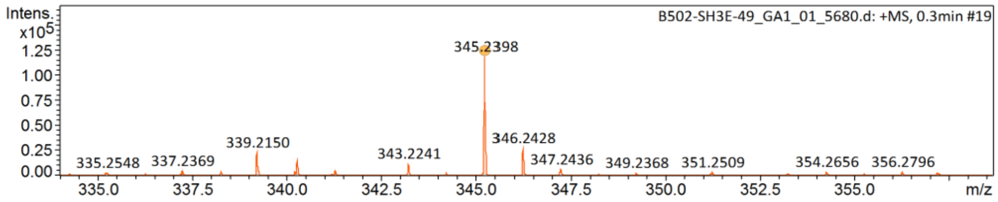

Meas. m/z	#	Ion Formula	m/z	err [ppm]	mSigma	# mSigma	Score	rdb	e⁻ Conf	N-Rule	Adduct
345.2398	1	C20H34NaO3	345.2400	0.6	13.9	1	100.00	4.0	even	ok	M+Na

Figure S44. HR-ESIMS spectrum of compound **5**

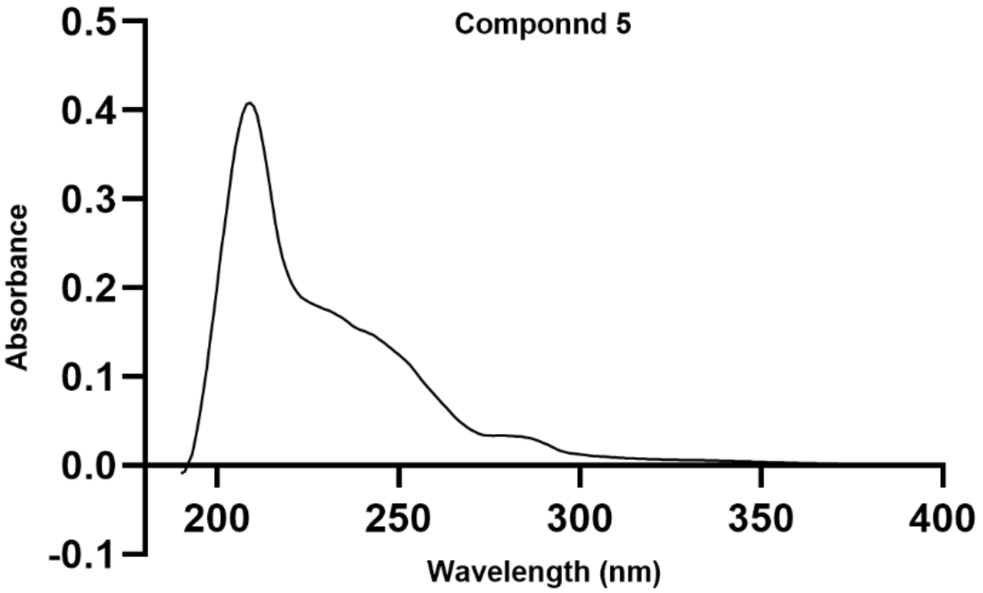

Figure S45. UV spectrum of compound **5**

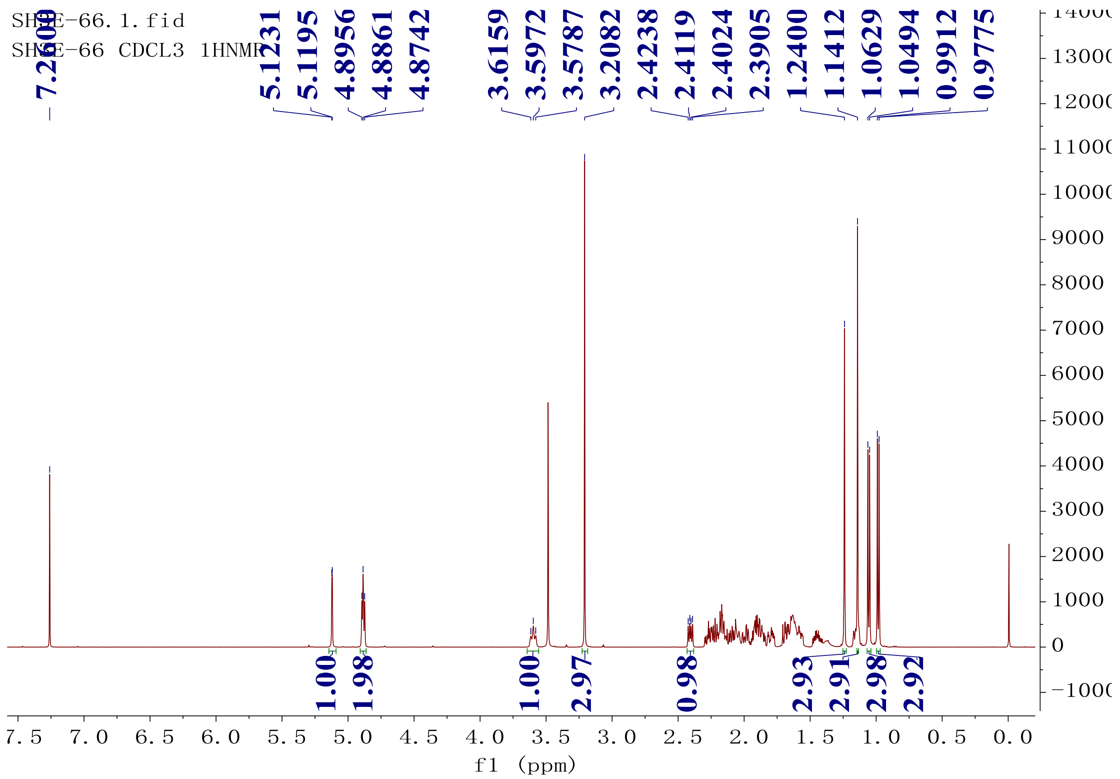

Figure S46. ^{1}H NMR spectrum (500 MHz) of compound **6** in $CDCl_3$

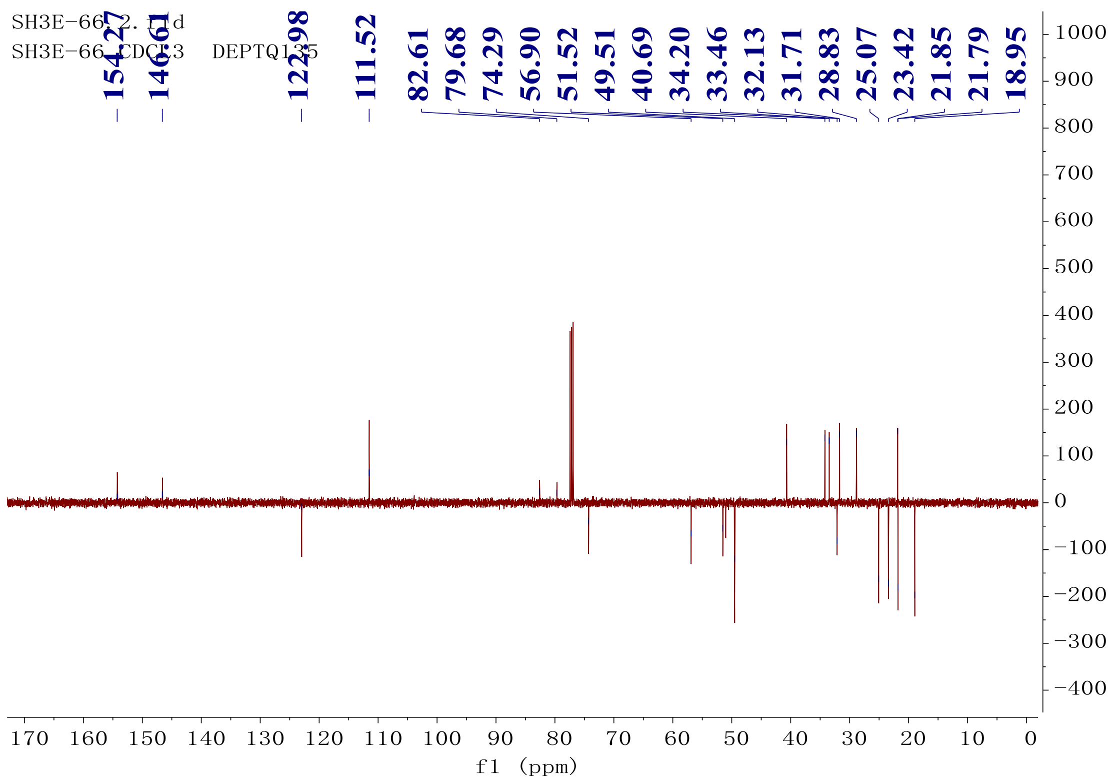

Figure S47. ^{13}C NMR spectrum (125 MHz) of compound **6** in $CDCl_3$

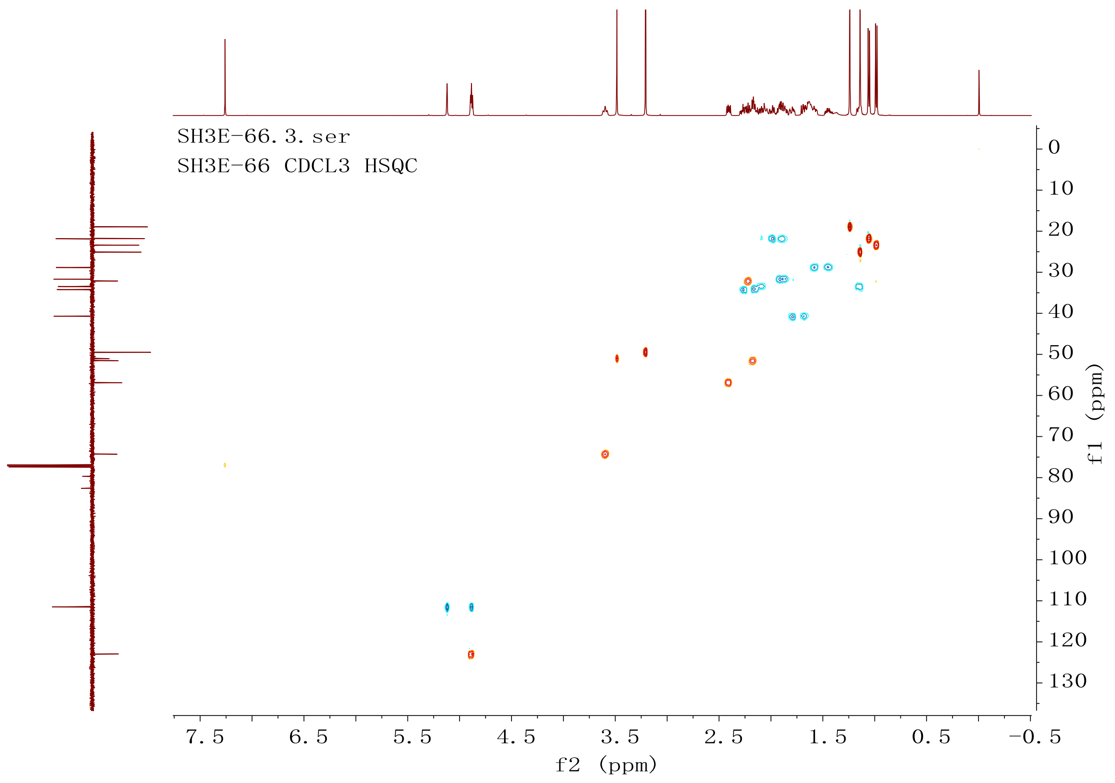

Figure S48. HSQC spectrum (500 MHz) of compound **6** in $CDCl_3$

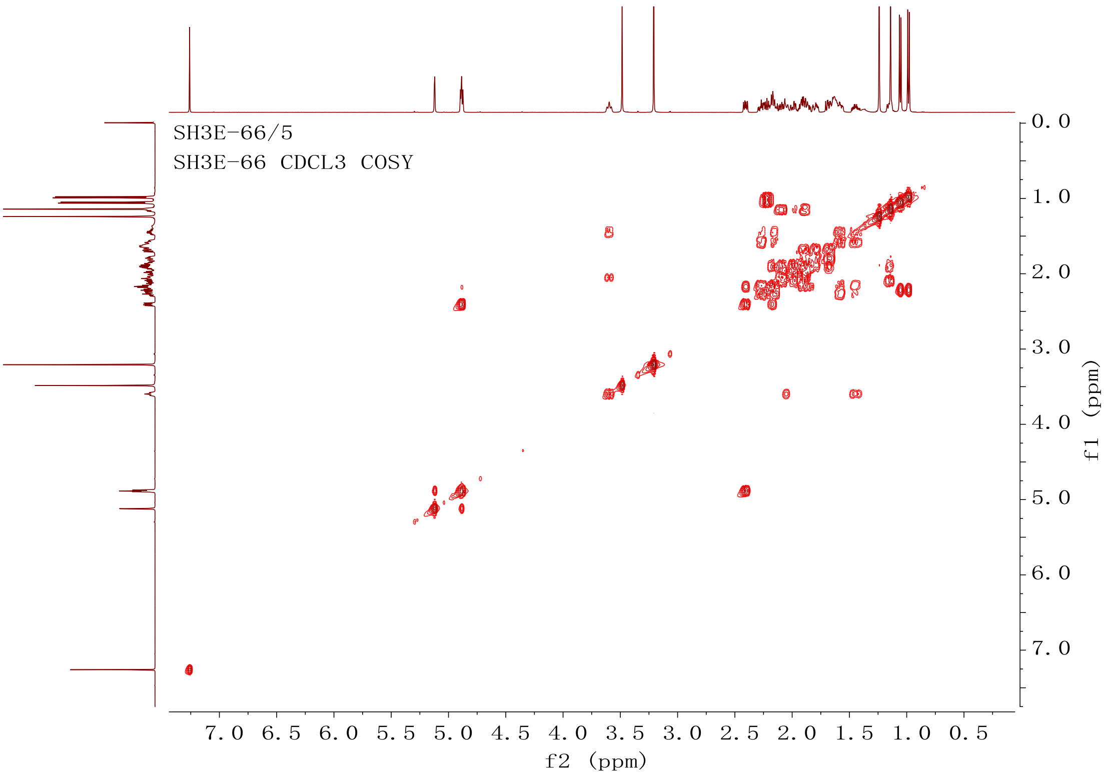

Figure S49. ^{1}H-^{1}H COSY spectrum (500 MHz) of compound **6** in $CDCl_3$

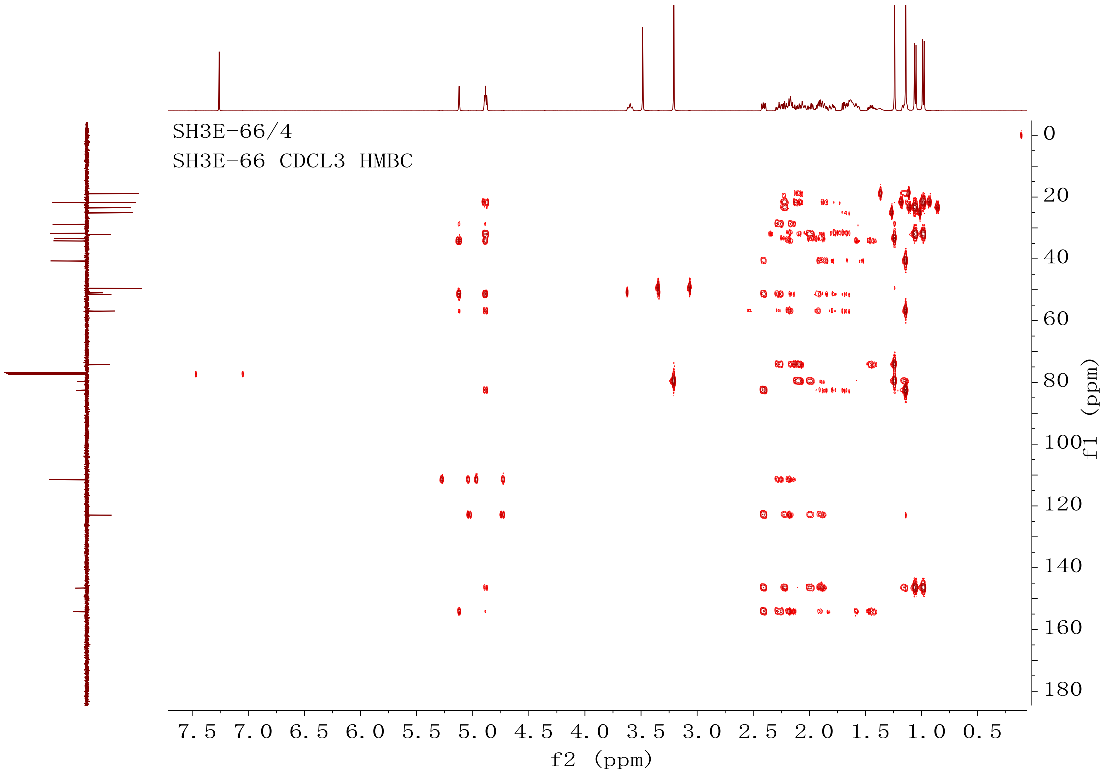

Figure S50. HMBC spectrum (500 MHz) of compound **6** in $CDCl_3$

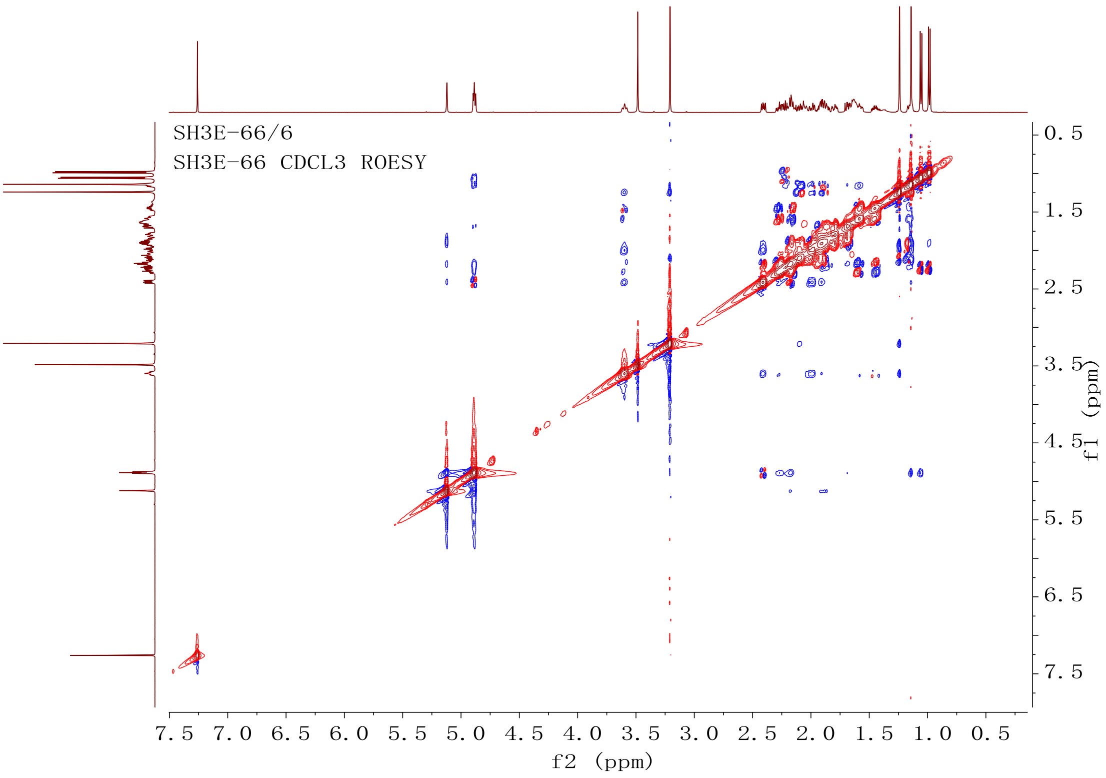

Figure S51. ROESY spectrum (500 MHz) of compound **6** in $CDCl_3$

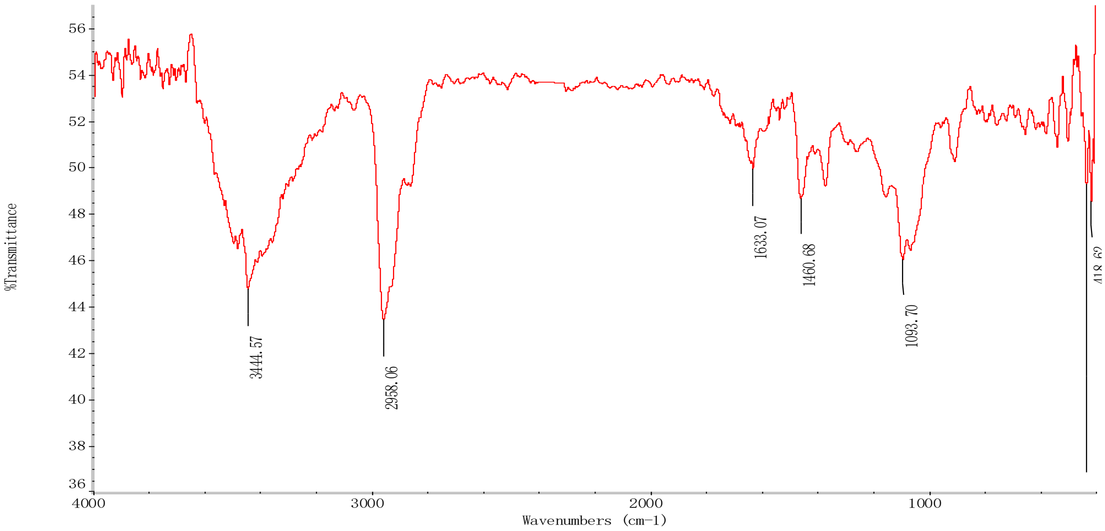

Figure S52. IR spectrum of compound **6**

Mass Spectrum SmartFormula Report

Analysis Info

Analysis Name	D:\Data\A501\WYL\2022\20221017\B502-SH3E-66_BE3_01_5119.d	Acquisition Date	2022-10-17 17:50:45
Method	lc-ms_as_ms-0.5MIN.m	Operator	Demo User
Sample Name	B502-SH3E-66	Instrument	compact 8255754.20156
Comment			

Acquisition Parameter

Source Type	ESI	Ion Polarity	Positive	Set Nebulizer	1.8 Bar
Focus	Not active	Set Capillary	4500 V	Set Dry Heater	220 °C
Scan Begin	50 m/z	Set End Plate Offset	-500 V	Set Dry Gas	4.0 l/min
Scan End	2500 m/z	Set Charging Voltage	2000 V	Set Divert Valve	Waste
		Set Corona	0 nA	Set APCI Heater	0 °C

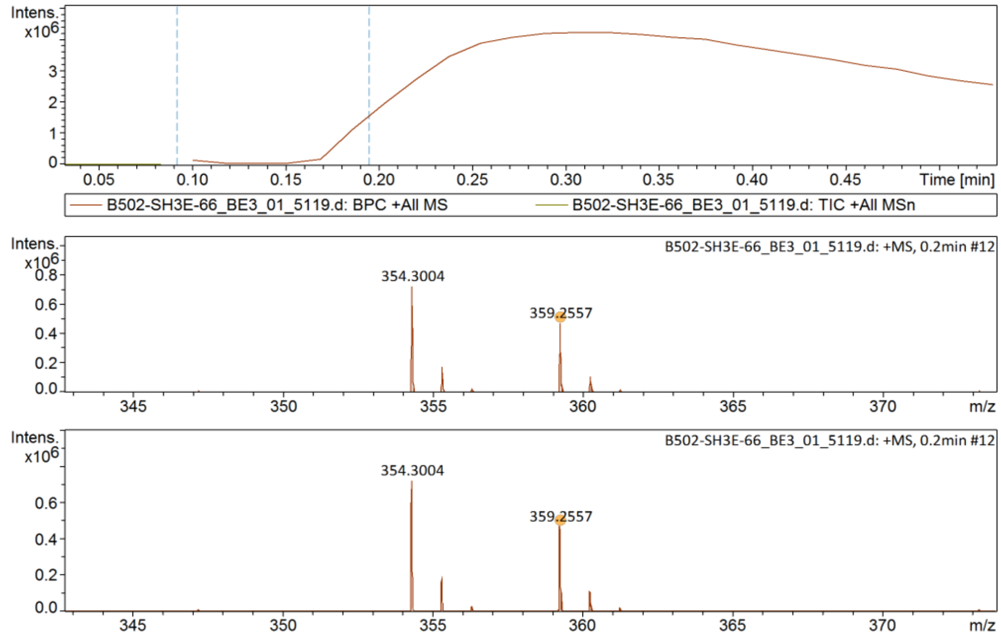

Meas. m/z	#	Ion Formula	m/z	err [ppm]	mSigma	# mSigma	Score	rdb	e⁻ Conf	N-Rule	Adduct
359.2557	1	C21H36NaO3	359.2557	-0.2	2.6	1	100.00	4.0	even	ok	M+Na

Figure S53. HR-ESIMS spectrum of compound **6**

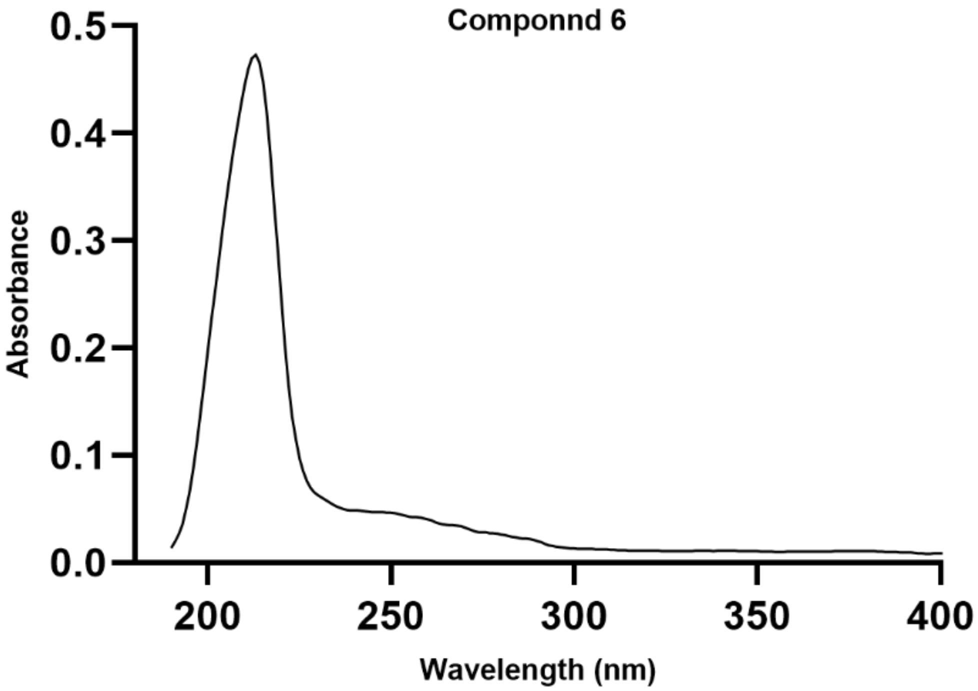

Figure S54. UV spectrum of compound **6**

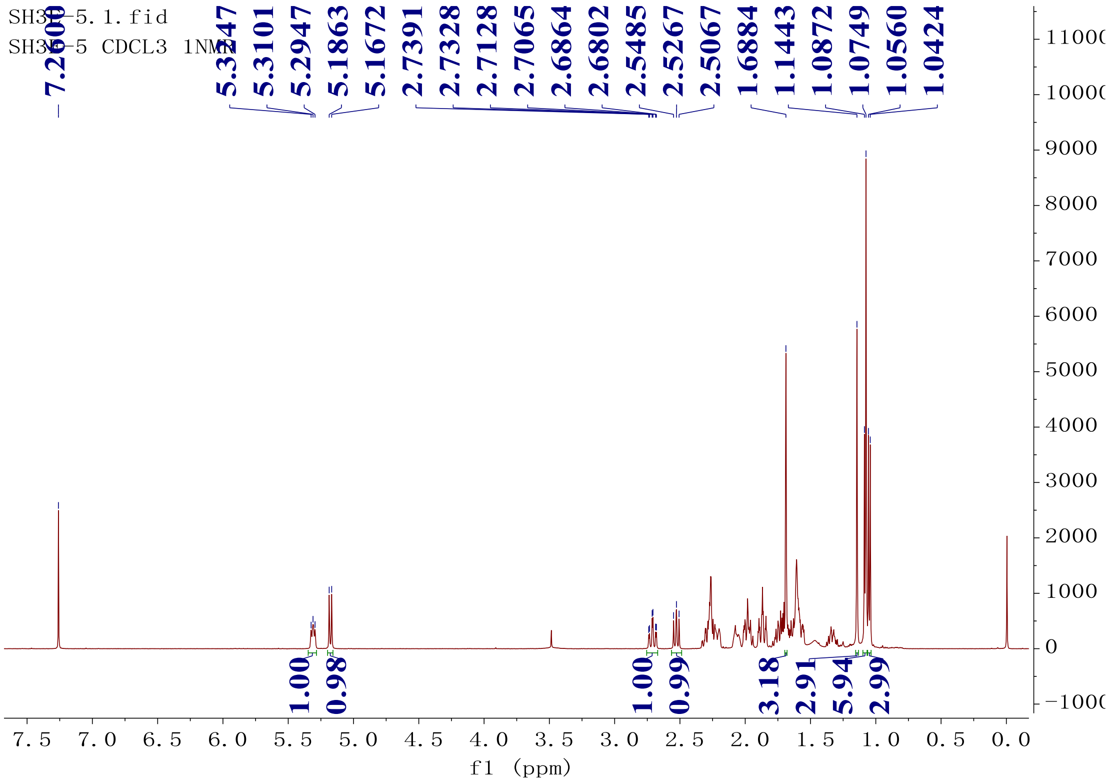

Figure S55. ^{1}H NMR spectrum (500 MHz) of compound **7** in $CDCl_3$

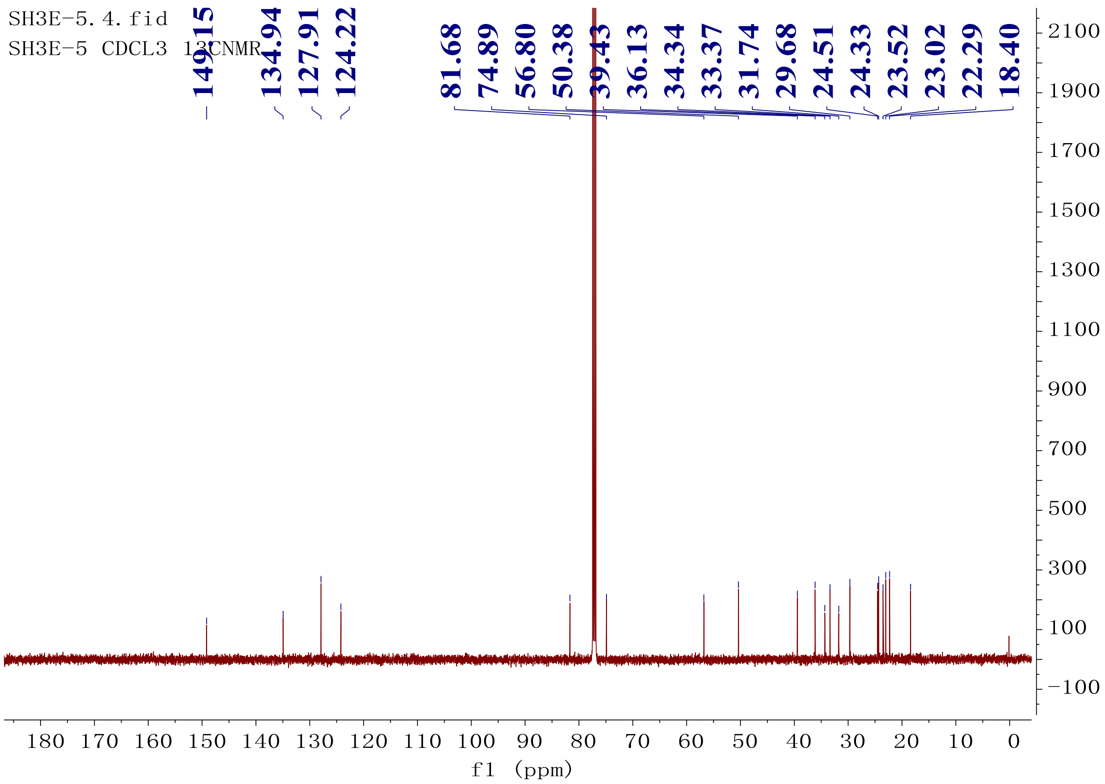

Figure S56. ^{13}C NMR spectrum (125 MHz) of compound **7** in $CDCl_3$

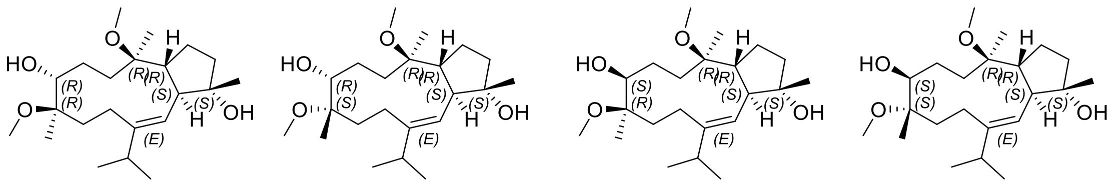

3a 3b 3c 3d

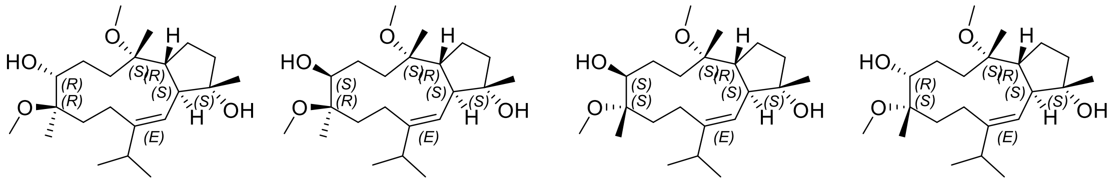

3e 3f 3g 3h

Figure S57. Structures of isomers of compound **3**

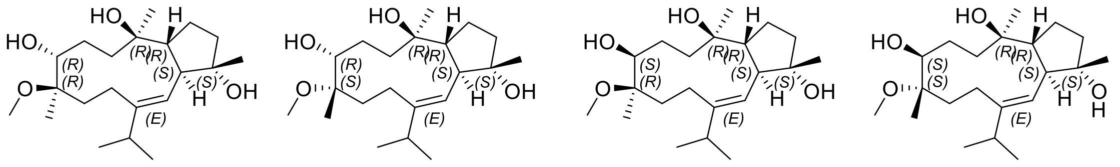

4a 4b 4c 4d

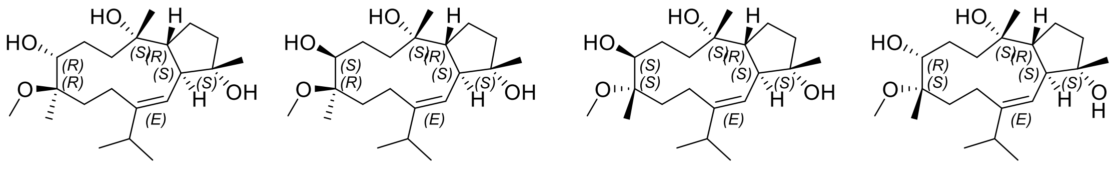

4e 4f 4g 4h

Figure S58. Structures of isomers of compound **4**

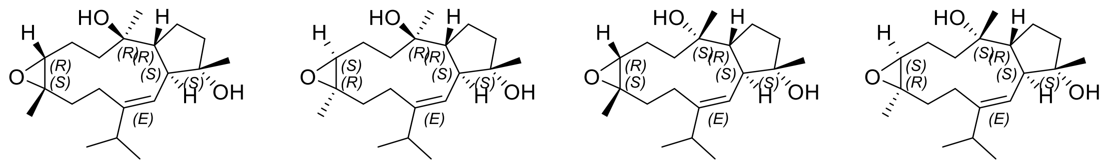

5a 5b 5c 5d

Figure S59. Structures of isomers of compound **5**

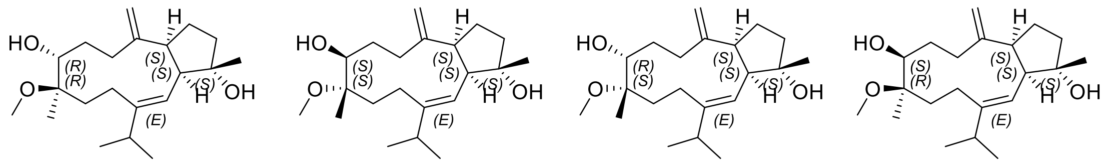

6a 6b 6c 6d

Figure S60. Structures of isomers of compound **6**

Isotropic shielding constants								
**Nuclei**	**1a**	**1b**	**1c**	**1d**	**1e**	**1f**	**1g**	**1h**
C 1	37.63	33.52	30.47	34.12	32.46	31.77	29.26	29.49
C 2	52.77	54.73	55.88	56.26	55.41	54.43	59.12	58.44
C 3	129.05	131.43	131.21	130.88	129.85	133.59	131.63	132.77
C 4	97.65	99.30	100.91	99.01	97.48	101.62	99.22	99.62
C 5	149.80	148.38	145.73	148.62	149.17	145.04	148.20	145.59
C 6	160.04	160.75	158.89	161.14	159.32	158.76	161.20	158.37
C 7	131.73	129.49	130.41	128.62	132.36	127.65	132.42	133.37
C 8	103.18	104.59	103.06	105.02	102.87	102.47	103.45	104.77
C 9	152.78	152.97	155.17	149.92	148.21	151.35	150.54	153.58
C 10	155.43	159.25	160.96	161.66	156.51	157.43	160.53	157.35
C 11	113.46	108.75	105.82	106.88	112.52	108.72	105.20	105.51
C 12	101.23	102.14	102.73	101.65	101.17	103.08	101.57	104.09
C 13	154.44	152.86	152.08	152.93	154.24	151.09	153.01	153.64
C 14	156.09	163.34	163.31	163.92	156.19	162.91	163.99	164.24
C 15	144.00	150.77	150.46	150.49	144.17	151.16	150.09	150.84
C 16	164.13	163.91	163.40	163.89	164.22	164.13	163.81	164.06
C 17	165.83	165.95	166.06	166.21	166.25	166.12	166.33	166.58
C 18	161.45	160.27	161.64	160.75	161.19	162.34	160.06	160.12
C 19	159.47	161.39	162.00	160.97	166.91	163.29	168.26	165.42
C 20	171.14	164.00	168.22	168.37	171.66	167.72	169.48	163.04
C 21	137.64	136.59	137.26	136.66	138.97	137.32	138.81	138.72
C 22	136.58	136.35	137.30	136.57	136.55	136.87	136.55	135.67

Table S1. Boltzmann averaged GIAO isotropic magnetic shielding constants (σ) of compound **1** calculated at the PCM/mPW1PW91/6-311G(d,p) //B3LYP/6-31G (d) level of theory.

Functional	Solvent?	Basis Set	Type of Data
mPW1PW91	PCM	6-311+G(d,p)	Shielding Tensors

	Isomer 1	Isomer 2	Isomer 3	Isomer 4	Isomer 5	Isomer 6	Isomer 7	Isomer 8
sDP4+ (H data)	0.00%	0.16%	0.02%	6.94%	0.04%	0.07%	74.29%	18.47%
sDP4+ (C data)	0.00%	0.00%	0.00%	0.00%	0.00%	0.00%	99.99%	0.01%
sDP4+ (all data)	0.00%	0.00%	0.00%	0.00%	0.00%	0.00%	100.00%	0.00%
uDP4+ (H data)	0.00%	26.77%	1.39%	2.14%	0.01%	0.07%	14.14%	55.49%
uDP4+ (C data)	0.00%	3.85%	0.09%	0.30%	0.00%	0.03%	2.39%	93.34%
uDP4+ (all data)	0.00%	1.94%	0.00%	0.01%	0.00%	0.00%	0.64%	97.41%
DP4+ (H data)	0.00%	0.20%	0.00%	0.71%	0.00%	0.00%	50.15%	48.94%
DP4+ (C data)	0.00%	0.00%	0.00%	0.00%	0.00%	0.00%	99.80%	0.20%
DP4+ (all data)	0.00%	0.00%	0.00%	0.00%	0.00%	0.00%	99.81%	0.19%

Table S2. DP4+ results obtained using experimental data of compound **1** *versus* isomers **1a**−**1h**.

Isotropic shielding constants								
**Nuclei**	**3a**	**3b**	**3c**	**3d**	**3e**	**3f**	**3g**	**3h**
C 1	33.58	26.51	30.47	33.85	32.04	31.65	33.14	28.68
C 2	53.06	59.06	55.88	53.79	51.29	54.10	54.53	53.65
C 3	130.92	131.96	131.21	133.91	133.34	133.92	133.68	132.08
C 4	99.41	102.05	100.91	102.34	100.04	101.53	101.94	100.29
C 5	146.04	148.39	145.73	144.55	145.28	144.91	144.87	144.94
C 6	159.61	160.61	158.89	160.44	157.99	158.43	159.65	158.87
C 7	132.13	140.04	130.41	128.59	122.31	127.37	127.65	133.78
C 8	102.89	104.08	103.06	103.27	105.64	102.39	102.96	104.04
C 9	154.79	151.77	155.17	153.52	150.10	151.59	149.56	153.12
C 10	154.82	157.13	160.96	155.92	159.87	156.61	156.26	156.13
C 11	113.80	103.07	105.82	106.60	108.03	109.88	106.53	112.01
C 12	101.61	102.69	102.73	102.11	101.87	101.41	102.61	101.63
C 13	154.44	144.76	152.08	152.07	153.87	152.22	149.32	154.95
C 14	155.35	163.30	163.31	164.47	156.46	163.33	161.55	159.60
C 15	144.19	150.17	150.46	151.73	147.85	151.12	152.18	149.97
C 16	163.49	162.85	163.40	164.00	164.23	163.97	164.48	162.45
C 17	165.68	166.16	166.06	166.29	165.85	166.09	166.38	164.25
C 18	162.24	163.42	161.64	163.49	161.59	162.33	162.88	161.16
C 19	159.64	161.32	162.00	167.66	162.60	163.38	164.99	160.24
C 20	170.93	169.96	168.22	163.00	168.50	168.35	166.66	168.75
C 21	137.81	138.00	137.26	137.80	136.35	137.10	137.61	137.61
C 22	136.40	133.12	137.30	135.57	136.61	136.20	135.81	137.66

Table S3. Boltzmann averaged GIAO isotropic magnetic shielding constants (σ) of compound **3** calculated at the PCM/mPW1PW91/6-311G(d,p) //B3LYP/6-31G (d) level of theory.

Functional	Solvent?	Basis Set	Type of Data
mPW1PW91	PCM	6-311+G(d, p)	Shielding Tensors

	Isomer 1	Isomer 2	Isomer 3	Isomer 4	Isomer 5	Isomer 6	Isomer 7	Isomer 8
sDP4+ (H data)	95.80%	0.00%	4.20%	0.00%	0.00%	0.00%	0.00%	0.00%
sDP4+ (C data)	0.00%	0.00%	100.00%	0.00%	0.00%	0.00%	0.00%	0.00%
sDP4+ (all data)	0.00%	0.00%	100.00%	0.00%	0.00%	0.00%	0.00%	0.00%
uDP4+ (H data)	0.00%	0.00%	100.00%	0.00%	0.00%	0.00%	0.00%	0.00%
uDP4+ (C data)	0.00%	0.00%	99.88%	0.01%	0.00%	0.01%	0.09%	0.00%
uDP4+ (all data)	0.00%	0.00%	100.00%	0.00%	0.00%	0.00%	0.00%	0.00%
DP4+ (H data)	0.02%	0.00%	99.98%	0.00%	0.00%	0.00%	0.00%	0.00%
DP4+ (C data)	0.00%	0.00%	100.00%	0.00%	0.00%	0.00%	0.00%	0.00%
DP4+ (all data)	0.00%	0.00%	100.00%	0.00%	0.00%	0.00%	0.00%	0.00%

Table S4. DP4+ results obtained using experimental data of compound **3** *versus* isomers **3a**–**3h**.

Isotropic shielding constants								
Nuclei	4a	4b	4c	4d	4e	4f	4g	4h
C 1	7.93	5.84	6.58	7.32	14.04	9.56	6.64	7.92
C 2	13.86	11.70	11.49	13.09	22.00	15.96	13.39	13.84
C 3	33.31	26.94	27.87	32.29	57.00	38.56	30.98	33.13
C 4	25.18	20.68	21.08	24.54	42.56	29.32	23.71	24.83
C 5	36.51	29.92	30.30	35.52	62.84	41.90	34.18	36.10
C 6	40.36	32.55	33.40	38.94	67.98	46.37	37.35	39.95
C 7	32.28	26.10	26.67	31.07	52.80	36.62	29.71	32.05
C 8	27.37	22.11	22.88	26.29	46.61	31.78	25.35	27.03
C 9	35.97	31.44	30.48	37.06	61.45	42.70	36.02	35.39
C 10	39.72	33.09	32.59	38.16	67.33	45.61	37.97	39.28
C 11	27.01	21.59	22.33	27.35	46.94	31.50	24.87	26.21
C 12	25.74	20.86	21.62	24.98	44.03	29.43	24.39	25.72
C 13	38.12	31.32	31.58	37.78	66.23	44.03	35.65	37.04
C 14	41.06	33.58	34.20	39.28	67.49	46.43	38.36	40.28
C 15	37.96	30.80	31.66	36.81	63.96	44.25	35.36	37.71
C 16	41.21	33.49	34.26	39.81	70.62	47.61	38.48	40.85
C 17	41.76	34.06	34.73	40.35	71.21	48.17	39.10	41.36
C 18	40.79	33.14	34.05	39.45	69.55	47.23	38.03	40.39
C 19	39.95	31.51	33.18	38.00	71.55	45.89	36.27	39.54
C 20	42.30	34.80	34.11	41.14	73.08	48.77	39.58	41.63
C 21	34.36	28.06	28.39	33.61	58.99	39.78	32.38	33.96
C 22	7.93	5.84	6.58	7.32	14.04	9.56	6.64	7.92

Table S5. Boltzmann averaged GIAO isotropic magnetic shielding constants (σ) of compound **4** calculated at the PCM/mPW1PW91/6-311G(d,p) //B3LYP/6-31G (d) level of theory.

Functional	Solvent?	Basis Set	Type of Data
mPW1PW91	PCM	6-311+G(d, p)	Shielding Tensors

	Isomer 1	Isomer 2	Isomer 3	Isomer 4	Isomer 5	Isomer 6	Isomer 7	Isomer 8
sDP4+ (H data)	-	-	-	-	-	-	-	-
sDP4+ (C data)	0.00%	32.09%	0.00%	0.00%	0.00%	0.00%	67.91%	0.00%
sDP4+ (all data)	0.00%	32.09%	0.00%	0.00%	0.00%	0.00%	67.91%	0.00%
uDP4+ (H data)	-	-	-	-	-	-	-	-
uDP4+ (C data)	0.00%	0.00%	0.00%	0.00%	100.00%	0.00%	0.00%	0.00%
uDP4+ (all data)	0.00%	0.00%	0.00%	0.00%	100.00%	0.00%	0.00%	0.00%
DP4+ (H data)	-	-	-	-	-	-	-	-
DP4+ (C data)	0.00%	0.00%	0.00%	0.00%	100.00%	0.00%	0.00%	0.00%
DP4+ (all data)	0.00%	0.00%	0.00%	0.00%	100.00%	0.00%	0.00%	0.00%

Table S6. DP4+ results obtained using experimental data of compound **4** *versus* isomers **4a–4h**.

Isotropic shielding constants				
Nuclei	5a	5b	5c	5d
C 1	31.54	30.54	34.06	32.03
C 2	53.43	53.93	49.30	55.63
C 3	131.96	132.11	133.44	133.37
C 4	100.43	99.70	99.01	100.19
C 5	144.26	145.59	145.33	145.07
C 6	159.22	157.58	159.17	160.55
C 7	124.95	126.86	122.52	129.88
C 8	109.58	107.19	111.86	109.18
C 9	153.65	141.92	149.46	144.88
C 10	158.09	160.34	161.50	160.41
C 11	121.73	119.03	118.65	119.70
C 12	123.64	119.60	122.90	120.49
C 13	146.14	151.66	155.42	150.12
C 14	158.77	160.66	154.20	159.69
C 15	151.03	150.44	143.62	151.11
C 16	166.09	166.01	165.87	166.27
C 17	162.49	163.80	163.57	164.19
C 18	161.16	160.75	162.34	162.71
C 19	153.40	161.75	155.89	160.80
C 20	31.54	30.54	34.06	32.03

Table S7. Boltzmann averaged GIAO isotropic magnetic shielding constants (σ) of compound **5** calculated at the PCM/mPW1PW91/6-311G(d,p) //B3LYP/6-31G (d) level of theory.

Functional	Solvent?		Basis Set		Type of Data
mPW1PW91	PCM		6-311G(d,p)		Shielding Tensors

	Isomer 1	Isomer 2	Isomer 3	Isomer 4	Isomer 5	Isomer 6
sDP4+ (H data)	-	-	-	-	-	-
sDP4+ (C data)	0.00%	0.26%	0.00%	99.74%	-	-
sDP4+ (all data)	0.00%	0.26%	0.00%	99.74%	-	-
uDP4+ (H data)	-	-	-	-	-	-
uDP4+ (C data)	0.00%	0.00%	0.00%	100.00%	-	-
uDP4+ (all data)	0.00%	0.00%	0.00%	100.00%	-	-
DP4+ (H data)	-	-	-	-	-	-
DP4+ (C data)	0.00%	0.00%	0.00%	100.00%	-	-
DP4+ (all data)	0.00%	0.00%	0.00%	100.00%	-	-

Table S8. DP4+ results obtained using experimental data of compound **5** *versus* isomers **5a**–**5d**.

Isotropic shielding constants				
Nuclei	6a	6b	6c	6d
C 1	6.10	6.70	8.30	9.50
C 2	9.70	10.40	12.80	14.10
C 3	19.20	19.10	24.40	26.40
C 4	15.00	15.10	19.40	21.00
C 5	21.10	21.30	27.40	29.50
C 6	22.50	22.60	29.10	31.10
C 7	19.30	19.40	25.20	27.00
C 8	5.40	6.20	8.20	8.50
C 9	21.50	21.40	27.60	29.70
C 10	22.10	21.90	28.50	30.40
C 11	16.20	16.20	21.70	22.30
C 12	15.27	15.34	19.80	21.34
C 13	22.41	22.17	28.54	30.64
C 14	23.25	23.30	29.07	32.20
C 15	21.74	21.77	27.78	30.24
C 16	23.49	23.58	29.78	32.36
C 17	23.43	23.31	29.76	32.74
C 18	23.04	22.93	29.48	31.87
C 19	11.20	11.58	15.07	16.04
C 20	26.17	24.03	30.94	33.10
C 21	20.01	19.89	25.57	27.60

Table S9. Boltzmann averaged GIAO isotropic magnetic shielding constants (σ) of compound **6** calculated at the PCM/mPW1PW91/6-31G(d,p) //B3LYP/6-31G (d) level of theory.

Functional	Solvent?		Basis Set		Type of Data
mPW1PW91	PCM		6-31G(d,p)		Shielding Tensors

	Isomer 1	Isomer 2	Isomer 3	Isomer 4	Isomer 5	Isomer 6
sDP4+ (H data)	-	-	-	-	-	-
sDP4+ (C data)	0.00%	2.04%	0.00%	97.95%	-	-
sDP4+ (all data)	0.00%	2.04%	0.00%	97.95%	-	-
uDP4+ (H data)	-	-	-	-	-	-
uDP4+ (C data)	0.05%	0.06%	12.29%	87.61%	-	-
uDP4+ (all data)	0.05%	0.06%	12.29%	87.61%	-	-
DP4+ (H data)	-	-	-	-	-	-
DP4+ (C data)	0.00%	0.00%	0.00%	100.00%	-	-
DP4+ (all data)	0.00%	0.00%	0.00%	100.00%	-	-

Table S10. DP4+ results obtained using experimental data of compound **6** *versus* isomers **6a–6d**.

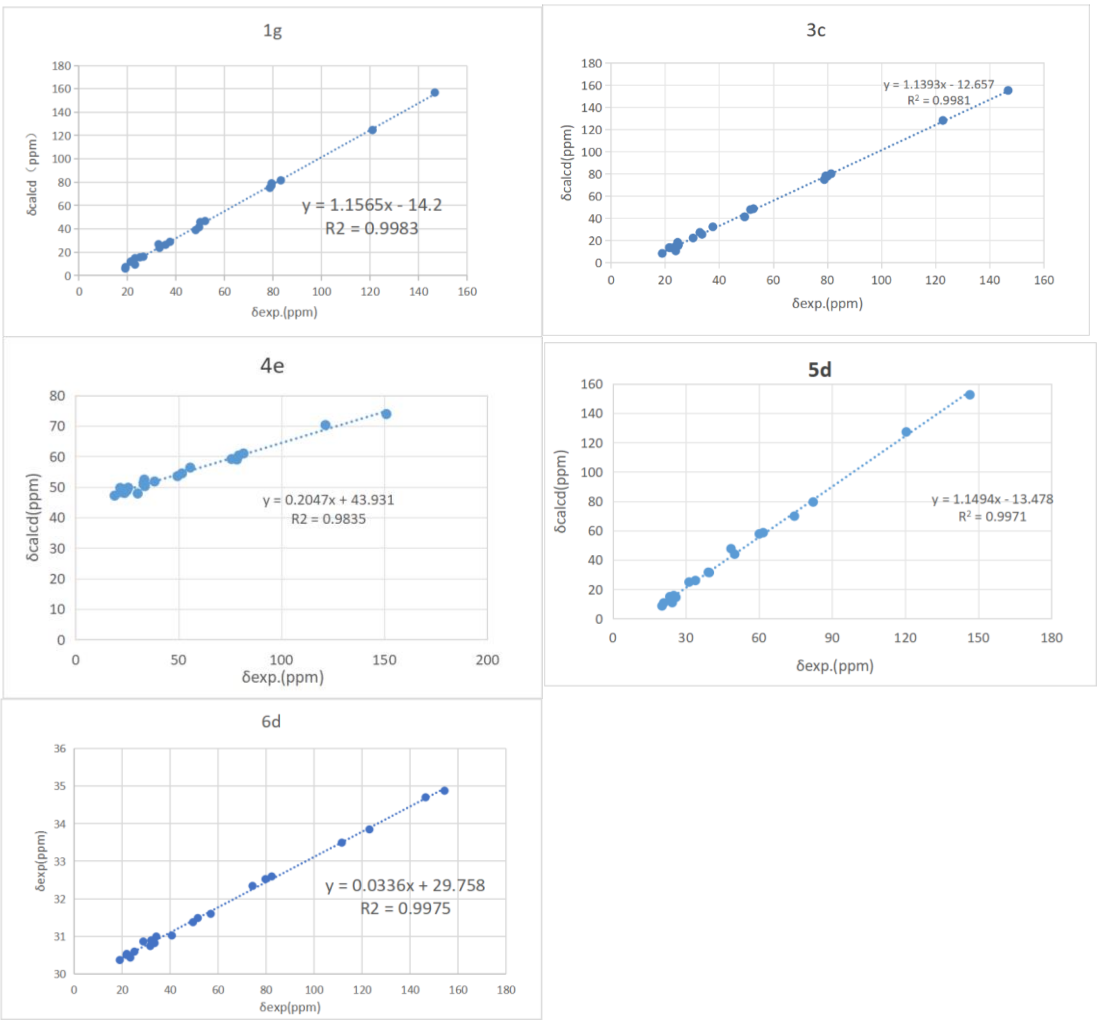

Figure S61. Correlation plots of compounds **1**, **3**−**6**

Table S11. Crystal data and structure refinement for **2**

Identification code	3e-83
Empirical formula	$C_{21}H_{37}O_{3.5}$
Formula weight	345.50
Temperature/K	169.99(10)
Crystal system	orthorhombic
Space group	$P2_12_12_1$
a/Å	9.5531(6)
b/Å	15.2772(15)
c/Å	28.949(5)
α/°	90
β/°	90
γ/°	90
Volume/Å^3	4224.9(8)
Z	8
ρ_{calc}g/cm^3	1.086
μ/mm^{-1}	0.564
F(000)	1528.0
Crystal size/mm^3	0.15 × 0.12 × 0.1
Radiation	Cu Kα (λ = 1.54184)
2Θ range for data collection/°	8.416 to 147.908
Index ranges	$-11 \le h \le 7$, $-18 \le k \le 18$, $-33 \le l \le 35$
Reflections collected	16223
Independent reflections	8320 [R_{int} = 0.1057, R_{sigma} = 0.2540]
Data/restraints/parameters	8320/391/471
Goodness-of-fit on F^2	1.021
Final R indexes [I>=2σ (I)]	R_1 = 0.0784, wR_2 = 0.1767
Final R indexes [all data]	R_1 = 0.2369, wR_2 = 0.2254
Largest diff. peak/hole / e Å^{-3}	0.50/-0.24
Flack parameter	-0.3(4)
